# Teixobactin analogues reveal enduracididine to be non-essential for highly potent antibacterial activity and lipid II binding†
†Electronic supplementary information (ESI) available: Peptide synthesis, HPLC, LC-MS analysis, NMR spectra, microbiological data (MIC, MBC, time kill kinetics), lipid II and geranyl pyrophosphate binding, cytotoxicity assay. See DOI: 10.1039/c7sc03241b


**DOI:** 10.1039/c7sc03241b

**Published:** 2017-10-05

**Authors:** Anish Parmar, Abhishek Iyer, Stephen H. Prior, Daniel G. Lloyd, Eunice Tze Leng Goh, Charlotte S. Vincent, Timea Palmai-Pallag, Csanad Z. Bachrati, Eefjan Breukink, Annemieke Madder, Rajamani Lakshminarayanan, Edward J. Taylor, Ishwar Singh

**Affiliations:** a School of Pharmacy , University of Lincoln , JBL Building, Beevor St. , Lincoln LN67DL , UK . Email: isingh@lincoln.ac.uk; b Organic and Biomimetic Chemistry Research Group , Department of Organic and Macromolecular Chemistry , Ghent University , Krijgslaan 281 (S4) , B-9000 Ghent , Belgium; c School of Chemistry , University of Lincoln , JBL Building, Beevor St. , Lincoln LN67DL , UK; d School of Life Sciences , University of Lincoln , JBL Building, Beevor St. , Lincoln LN67DL , UK; e Singapore Eye Research Institute , The Academia, Discovery Tower Level 6, 20 College Road , Singapore 169857; f Department of Membrane Biochemistry and Biophysics , Institute of Biomembranes , Utrecht University , Padualaan 8 , 3584 CH Utrecht , The Netherlands

## Abstract

Teixobactin is a highly promising antibacterial depsipeptide consisting of four d-amino acids and a rare l-*allo*-enduracididine amino acid. l-*allo*-Enduracididine is reported to be important for the highly potent antibacterial activity of teixobactin. However, it is also a key limiting factor in the development of potent teixobactin analogues due to several synthetic challenges such as it is not commercially available, requires a multistep synthesis, long and repetitive couplings (16–30 hours). Due to all these challenges, the total synthesis of teixobactin is laborious and low yielding (3.3%). In this work, we have identified a unique design and developed a rapid synthesis (10 min μwave assisted coupling per amino acid, 30 min cyclisation) of several highly potent analogues of teixobactin with yields of 10–24% by replacing the l-*allo*-enduracididine with commercially available non-polar residues such as leucine and isoleucine. Most importantly, the Leu_10_-teixobactin and Ile_10_-teixobactin analogues have shown highly potent antibacterial activity against a broader panel of MRSA and *Enterococcus faecalis* (VRE). Furthermore, these synthetic analogues displayed identical antibacterial activity to natural teixobactin (MIC 0.25 μg mL^–1^) against MRSA ATCC 33591 despite their simpler design and ease of synthesis. We have confirmed lipid II binding and measured the binding affinities of individual amino acid residues of Ala_10_-teixobactin towards geranyl pyrophosphate by NMR to understand the nature and strength of binding interactions. Contrary to current understanding, we have shown that a cationic amino acid at position 10 is not essential for target (lipid II) binding and potent antibacterial activity of teixobactin. We thus provide strong evidence contrary to the many assumptions made about the mechanism of action of this exciting new antibiotic. Introduction of a non-cationic residue at position 10 allows for tremendous diversification in the design and synthesis of highly potent teixobactin analogues and lays the foundations for the development of teixobactin analogues as new drug-like molecules to target MRSA and *Mycobacterium tuberculosis*.

## Abstract 2

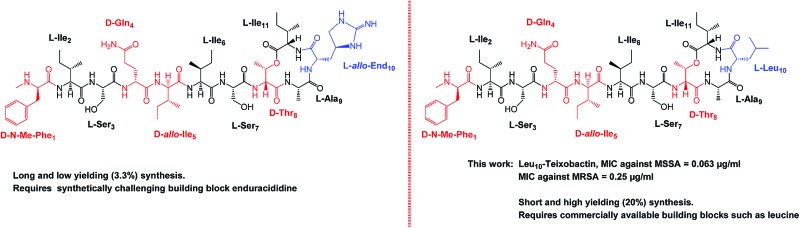
Leu_10_-teixobactin and Ile_10_-teixobactin have shown comparable activity to natural teixobactin.

## Introduction

By 2050, antimicrobial resistance (AMR) is predicted to cause more mortality than cancer.^1^ To address the challenges of AMR, there is a continual need to develop new antibacterial compounds. The recently discovered molecule teixobactin is one such very promising antibacterial.^2^ Teixobactin kills bacteria such as Methicillin-resistant *Staphylococcus aureus* (MRSA) and *Mycobacterium tuberculosis* without detectable resistance.^2^ In 2015, *Mycobacterium tuberculosis* infected 10.4 million individuals and was responsible for 1.5 million deaths worldwide.^3^ Teixobactin kills bacteria through the inhibition of cell wall synthesis.^2^ The development of resistance against teixobactin is predicted to be low because it targets cell wall substrates from multiple biosynthetic pathways.^4^ Teixobactin, thus, does not target enzymes, but multiple bactoprenol-coupled cell wall building blocks such as lipid II (needed for peptidoglycan synthesis) and lipid III (a starting material for cell wall teichoic acid synthesis) leading to inhibition of cell wall synthesis.^5^ The targeting of building blocks (lipid II and lipid III) rather than enzymes makes it inherently more difficult for bacteria to develop resistance. The inhibition of cell wall synthesis *via* multiple pathways results in cell wall damage, lysis (due to delocalisation of autolysins, lytic enzymes) and cell death. The aforementioned reasons make teixobactin an outstanding molecule for antibiotic development.^6^

**Fig. 1:**
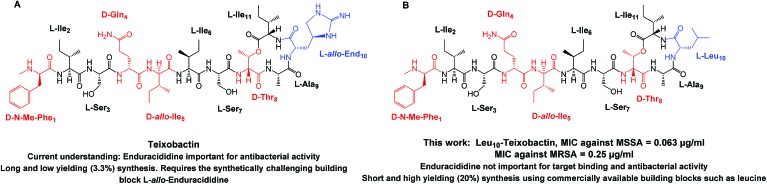
Structure of (A) teixobactin (**16**) and (B) Leu_10_-teixobactin (**13**) with the d amino acids highlighted in red and the l-*allo*-end and replaced l-Leu residue highlighted in blue. MRSA ATCC 33591, *Staphylococcus aureus* ATCC 29213.

Teixobactin is an undecapeptide containing four d amino acids namely *N*-Me-d-Phe_1_, d-Gln_4_, d-*allo*-Ile_5_ and d-Thr_8_ (Fig. 1, marked in red) and the rare l-*allo*-enduracididine amino acid^7^ (Fig. 1A, marked in blue). In the past year, articles have been published which describe the total synthesis of teixobactin,^8^,^9^ and the syntheses and biological activities of teixobactin analogues.^10^–^12^ The synthesis of Arg_10_-teixobactin analogues was reported by us^11^ and others.^10^,^12^ Arg_10_-teixobactin was obtained by replacing the l-*allo*-enduracididine amino acid at position 10 with the structurally similar, commercially available arginine. Arg_10_-teixobactin showed a similar trend in terms of antibacterial activity as teixobactin. Our previous work^13^ established the importance of d amino acids *via* the total syntheses and biological evaluation of the d and l analogues of Arg_10_-teixobactin. Changing the amino acid configuration of any one of the four d amino acids (*N*-Me-d-Phe_1_, d-Gln_4_, d-*allo*-Ile_5_ and d-Thr_8_) from d to l leads to significant loss in antibacterial activity. This work also defined the 3D molecular structure of seven teixobactin analogues whereby (1) the disordered structure of these analogues were found to be vital for biological activity, (2) d-Gln_4_ is essential and (3) d-*allo*-Ile_5_ is important to maintain the disordered structure.^13^

Nowick *et al.*^12^ reported a minimum pharmacophore of teixobactin coined lipobactin by replacing the N-terminal residues **1–5** with a dodecanoyl chain. They described that any change to d-Thr_8_ or modification of configuration of any of the residues in the core ring structure of teixobactin results in a significant decrease in activity. The enantiomeric Arg_10_-teixobactin reported by them shows similar biological activity as Arg_10_-teixobactin. These results suggest that, in order to maintain biological activity, only the relative configurations of amino acids are important and not their absolute configurations. Very recently, Nowick *et al.* also reported the X-ray crystallographic structure of a teixobactin analogue and described its hydrophobic interactions, as well as a hydrogen bonding cavity featuring a chloride ion.^14^ The Albericio group has reported a lysine scan of Arg_10_-teixobactin.^15^ They concluded that the replacement of any one of the four isoleucine residues with lysine led to complete loss of activity. However, replacement of the polar, non-charged residues Ser_3_, Gln_4_ and non-polar alanine by lysine resulted in analogues with comparable biological activities to that of Arg_10_-teixobactin. Wu C. *et al.* have reported that the guanidine of arginine or amine of lysine at position 10, Ser_7_ and the NH group of N terminal phenylalanine are all critical for the biological activity of teixobactin analogues.^16^ The replacement of the arginine or lysine at position 10 by histidine, Ser_7_ by alanine (including analogue **7**) and *N*-methyl phenylalanine_1_ by *N*,*N*-dimethyl phenylalanine leads to less active teixobactin analogues compared to the Arg_10_-teixobactin. Recently, we have reported the synthesis of teixobactin analogues by replacing l-*allo*-enduracididine with isosteres such as homoarginine_10_-teixobactin, norarginine_10_-teixobactin. These analogues showed promising activity against MRSA. However these were less active than natural teixobactin.^17^

**Fig. 2:**
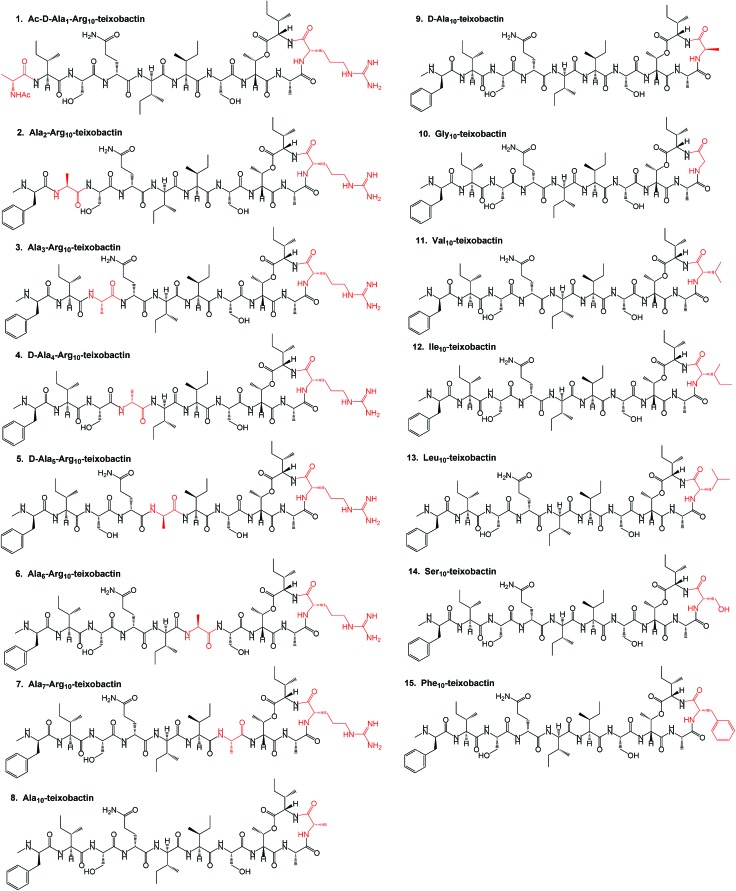
Structure of teixobactin analogues **1–15** synthesised with the replaced amino acids highlighted in red.

**Fig. 3:**
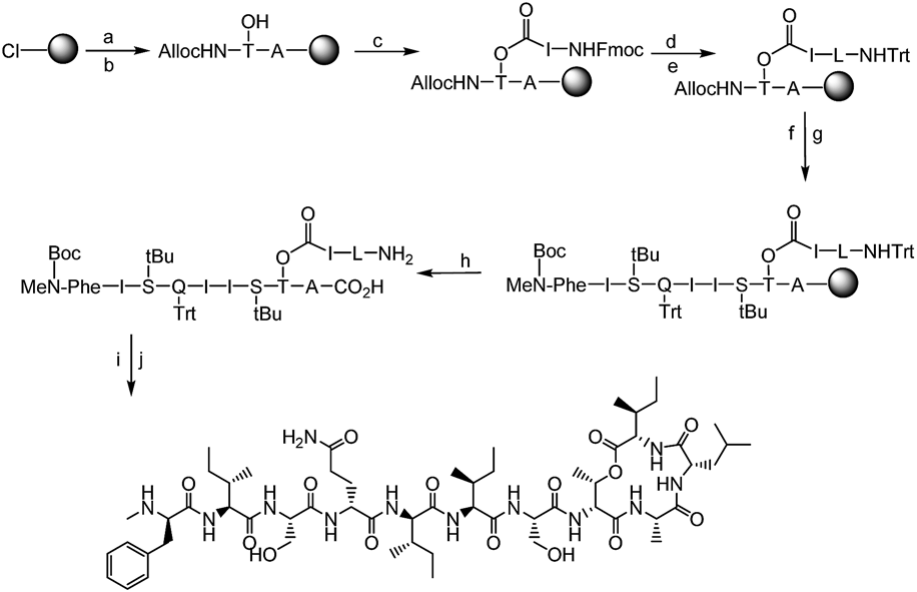
Synthesis of Leu_10_-teixobactin starting from 2-chlorotritylchloride resin: (a) 4 eq. Fmoc-Ala-OH/8 eq. DIPEA in DCM, 3 h. (b) 20% piperidine in DMF followed by 3 eq. AllocHN-d-Thr-OH, 3 eq. HATU/6 eq. DIPEA, 1.5 h. (c) 10 eq. Fmoc-Ile-OH, 10 eq. DIC, 5 mol% DMAP in DCM, 2 h followed by capping with Ac_2_O/DIPEA 10% in DMF, 20% piperidine in DMF. (d) 4 eq. Fmoc-Leu-OH, 4 eq. HATU/8 eq. DIPEA in DMF, 1 h followed by 20% piperidine in DMF. (e) 10 eq. Trt-Cl, 15% Et_3_N in DCM, 1 h. (f) 0.2 eq. [Pd(PPh_3_)_4_]^0^ + 24 eq. PhSiH_3_ in dry DCM, 1 × 20 min, 1 × 45 min. (g) 4 eq. Fmoc/Boc-AA(PG)-OH (AA = amino acid, PG = protecting group), 4 eq. DIC/Oxyma (μwave, 10 min) followed by 20% piperidine in DMF (3 min, 10 min). (h) TFA : TIS : DCM = 2 : 5 : 93, 1 h. (i) 1 eq. HATU/10 eq. DIPEA in DMF, 30 min. (j) TFA : TIS : H_2_O = 95 : 2.5 : 2.5, 1 h.

In this work, we describe a unique design and rapid synthesis of several highly potent analogues of teixobactin against *Staphylococcus aureus* (MSSA), methicillin-resistant *Staphylococcus aureus* (MRSA) and *Enterococcus faecalis* (vancomycin-resistant *Enterococci*, VRE) by replacing the synthetically challenging l-*allo*-enduracididine with commercially available non-polar residues such as alanine, leucine and isoleucine. This study aims to answer two important questions. Firstly, is it essential to incorporate a residue with a positively charged side chain at position 10 for maintaining target binding (lipid II) and biological activity of teixobactin and its analogues? Secondly, what are the key residues involved and what are the target binding contributions of the individual amino acid residues in the teixobactin analogues? To evaluate both these questions and identify the key residues particularly with respect to position 10, an alanine scan was performed on Arg_10_-teixobactin (Fig. 2, **1–8**). The alanine scanning technique has been used earlier on other antimicrobial peptides with success^18^ but has not yet been performed on teixobactin or its analogues. In order to further improve the antibacterial activity of Arg_10_-teixobactin by modifying the amino acid at position 10, new analogues of teixobactin were prepared by systematic replacement of Arg_10_ with d-Ala (d-Ala_10_-teixobactin, **9**), Gly (Gly_10_-teixobactin, **10**), Val (Val_10_-teixobactin **11**), Ile (Ile_10_-teixobactin, **12**), Leu (Leu_10_-teixobactin, **13**), Ser (Ser_10_-teixobactin, **14**) and Phe (Phe_10_-teixobactin, **15**) (Fig. 2, **9–15**). We thus synthesised **15** analogues (Fig. 2) of teixobactin using the conditions described in Fig. 3, Page S3.†

## Results and discussion

### Design and syntheses

In our ongoing research to develop potent teixobactin analogues against resistant bacteria, we are particularly interested in understanding the role of polar amino acid residues at position 10, namely; l-*allo*-enduracididine, arginine and lysine. l-*allo*-Enduracididine is important for the biological activity of teixobactin and its replacement by Arg results in decreased biological activity (Table 1). However, it is also a key limiting factor in the development of potent teixobactins due to several synthetic challenges such as (1) it is not commercially available, (2) it requires a multistep synthesis (7 steps, 17% yield),^8^ (3) a suitably protected l-*allo*-enduracididine is difficult to incorporate into teixobactin and (4) it requires long and multiple couplings (16–30 hours).^8^,^9^ Due to all of these challenges, the synthesis of teixobactin is laborious and low yielding (3.3%).

**Table 1:** List of teixobactin analogues (**1–15**)

Compound	Name	MIC^*a*^ (μg mL^–1^)
**1**	Ac-d-Ala_1_-Arg_10_-teixobactin	>128
**2**	Ala_2_-Arg_10_-teixobactin	>128
**3**	Ala_3_-Arg_10_-teixobactin	1–2
**4**	d-Ala_4_-Arg_10_-teixobactin	2–4
**5**	d-Ala_5_-Arg_10_-teixobactin	64–128
**6**	Ala_6_-Arg_10_-teixobactin	>128
**7**	Ala_7_-Arg_10_-teixobactin	16–32
**8**	Ala_10_-teixobactin	1–2
**9**	d-Ala_10_-teixobactin	32
**10**	Gly_10_-teixobactin	2
**11**	Val_10_-teixobactin	0.5
**12**	Ile_10_-teixobactin	0.25
**12a**	Ile_10_-teixobactin + 10% human serum^*b*^	0.25
**13**	Leu_10_-teixobactin	0.25
**13a**	Leu_10_-teixobactin + 10% human serum^*b*^	0.25
**14**	Ser_10_-teixobactin	16
**15**	Phe_10_-teixobactin	2
**16**	Arg_10_-teixobactin	2
**17**	Teixobactin	0.25
**18**	Vancomycin	2

^*a*^MIC: minimum inhibitory concentration. MRSA ATCC 33591 used. Culture media: Mueller Hinton broth (Oxoid).

^*b*^10% volume with human serum (SIGMA, H4522).

The synthesis of all our analogues involved loading Fmoc-alanine on the 2-chlorotritylchloride resin, followed by amide coupling with alloc-NH-d-Thr-OH, resin esterification with 10 eq. Fmoc-Ile-OH, and adding 10 eq. DIC and 5 mol% DMAP for 2 h. The next amino acid (AA) was then coupled using 4 eq. AA with 4 eq. HATU/8 eq. DIPEA in DMF for 1 h followed by Fmoc deprotection and trityl protection. Next, the N-terminal alloc protecting group was removed using Pd(PPh_3_)_4_ and phenylsilane (Fig. 3). All other amino acids were coupled using 4 eq. AA with 4 eq. DIC/Oxyma using an automated microwave peptide synthesiser (coupling time of 10 min each). Fmoc deprotection was performed using 20% piperidine in DMF (Fig. 3, Page S3†). Cyclisation was performed using 1 eq. HATU and 10 eq. DIPEA and was found to be complete within 30 min with complete conversion of the linear product into its cyclised counterpart (Pages S5–S32†). Yields after HPLC purification were found to be 10–24% (Table S1†). We have identified a unique design in which the introduction of hydrophobic residues such as leucine at position 10 (Fig. 1) has several advantages over the lengthy low-yielding (3.3%) synthesis of teixobactin, including overall yields of up to 24%, faster automated syntheses, and use of commercially available building blocks.

Teixobactin and its active analogues such as Arg_10_-teixobactin and Lys_10_-teixobactin contain two positive charges. However, the analogues **1**, **8–15** contain only one positive charge and were therefore found to be more hydrophobic than the analogues **2–7**. All the compounds were found to be completely soluble in DMSO. Therefore, stock solutions of these compounds were prepared in DMSO for MIC testing. Upon dilution in the Mueller Hinton broth (Oxoid) culture media in which bacteria were grown (concentration ∼256 μg mL^–1^), no turbidity or precipitation was observed indicating that the compounds were soluble in the culture media.

### Antibacterial studies

Analogues of teixobactin derived through an alanine scanning of teixobactin reveal that residues *N*-Me-Phe_1_, Ile_2_d-*allo*-Ile_5_, l-Ile_6_ and Ser_7_ are important for antibacterial activity and their replacement by l-Ala or d-Ala results in decrease in biological activity (Table 1). Interestingly, replacement of l-Ser_3_ and d-Gln_4_ by l-Ala and d-Ala has no effect on antibacterial activity. Thus, the two residues l-Ser_3_ & d-Gln_4_ are ideal candidates for replacement in the case of teixobactin due to their more facile synthesis and minimal impact on biological activity. It has been suggested that replacement of any of the residues in the core ring structure of teixobactin negates all biological activity of the molecule.^12^ In our case, however, the most interesting result was obtained through the replacement of l-Arg_10_ by l-Ala (**8**).

The design and syntheses of potent teixobactin analogues published in the literature has thus far been limited to the substitution of l-*allo*-enduracididine with amino acids such as Arg,^10^,^11^ Lys^12^ and Orn,^9^ all of which possess a cationic side chain. A positive charge is a common structural characteristic of depsipeptides which bind to lipid II.^4^l-*allo*-Enduracididine is thus reported to be important for potent antibacterial activity of teixobactin.^8^ Therefore, it was expected that replacement of this residue with alanine, which is non-polar and uncharged, would completely abolish the biological activity of the molecule. Contrary to this, we observed that Ala_10_-teixobactin was highly active against MRSA (Table 1) with an MIC of 1–2 μg mL^–1^.

A plausible explanation could be that Ala_10_-teixobactin binds to the pyrophosphate motif of lipid II using the amide backbone in a similar way to that proposed for the binding of nisin.^19^ Superior results were obtained with Ile_10_-teixobactin (**12**) and Leu_10_-teixobactin (**13**), which consistently gave identical MIC values of 0.25 μg mL^–1^ as compared to the teixobactin against MRSA (Table 1). Leu has a very similar hydrocarbon framework to l-*allo*-enduracididine (Fig. 1), followed very closely by Ile, which could explain the identical MIC value of these analogues (**12** & **13**) to teixobactin (**17**) against MRSA. In order to determine the effect of serum on antibacterial activity, the MIC of compounds **12** and **13** were measured in presence of 10% human serum (Page S53†). In both cases no change was observed in the MIC (Table 1) indicating that 10% human serum has no effect on the antibacterial activity.

The fact that a cationic residue at position 10 is not essential for antibacterial activity represents a significant breakthrough in teixobactin research given the earlier stated importance of the l-*allo*-enduracididine amino acid in the total synthesis of teixobactin.^8^ Our design has considerably improved not only the antibacterial activity of teixobactin analogues but also the ease of synthesis. Our findings are of particular importance as MRSA is responsible for many infections worldwide.^20^

d-Ala_10_-teixobactin shows 16-times lower antibacterial activity than Arg_10_-teixobactin which would be expected, as inversion of configuration of even a single amino acid in the core ring structure can significantly lower the MIC value of a teixobactin analogue.^12^ Surprisingly, Gly_10_-teixobactin (**10**) shows identical activity to Arg_10_-teixobactin (**16**) showing that complete removal of the chiral center at position 10 is tolerated provided the configuration of the remaining residues is intact. Val_10_-teixobactin (**11**) shows 4-times better antibacterial activity than Arg_10_-teixobactin but Ser_10_-teixobactin (**14**) shows 8-times lower activity, indicating that Ser at position 10 probably interferes with hydrogen bonding between the core ring structure of teixobactin and lipid II. Phe_10_-teixobactin (**15**) gave an MIC of 2 μg mL^–1^ against MRSA indicating that an aromatic amino acid such as phenylalanine at position 10 is also tolerated. Overall, from our work it appears that the claimed importance of a charged residue at position 10 in the form of an amine or guanidine group in teixobactin has been overstated in the literature given that the most potent analogues obtained thus far are the Leu_10_-teixobactin and Ile_10_-teixobactin both of which are non-polar and non-charged. This unexpected result facilitates the development of several highly potent teixobactin analogues against a broader panel of MRSA, MSSA and *Enterococcus faecalis* (VRE) including *Mycobacterium smegmatis* (Table 2) but with significantly higher yields compared to teixobactin. Although analogues of teixobactin with improved yields have been synthesised previously,^10^–^16^ none possess comparable activity to teixobactin and therefore the yields obtained for Ile_10_-teixobactin and Leu_10_-teixobactin (Table S1, page S4 and Table S4, page S54,† 10–20%) cannot be compared to those of the other less potent analogues of teixobactin described in literature.^10^–^16^ Based on the initial MIC results (Table 1), we identified Ala_10_-teixobactin (**8**), Val_10_-teixobactin (**11**), Ile_10_-teixobactin (**12**) and Leu_10_-teixobactin (**13**) as our lead compounds. These compounds along with Arg_10_-teixobactin (**16**), and vancomycin/daptomycin as controls, were tested against an extended panel of Gram positive bacteria (Table 2) to provide a more comprehensive overview of the biological activity of these molecules. A significant difference in MIC was observed in the presence and absence of polysorbate 80 (Table S4, page S54†).^2^ Leu_10_-teixobactin (**13**) showed potent activity against *M. smegmatis* (MIC ∼1 μg mL^–1^). Ala_10_-teixobactin (**8**) and Arg_10_-teixobactin (**16**) showed comparable activity against *M. smegmatis* with MICs in the range of 1–2 μg mL^–1^. In general, the MBCs of all compounds were found to be 2–4 times the MIC value. Ile_10_-teixobactin (**12**) and Leu_10_-teixobactin (**13**) were found to be the most potent compounds showing MICs ≤ 0.25 μg mL^–1^ in all strains. Ile_10_-teixobactin (**12**) in particular was found to be highly active against both VRE strains with MICs ≤ 0.0625 μg mL^–1^ and also an MIC 0.5 of μg mL^–1^ against *M. smegmatis*. We thus report, for the first time, two analogues of teixobactin showing highly potent antibacterial activity against a broader panel of resistant Gram positive bacteria. This is a very significant advancement in terms of teixobactin research and allows for the synthesis of a library of teixobactin derivatives based on Ile_10_-teixobactin and Leu_10_-teixobactin which can be simpler, highly potent and significantly more cost effective than the synthesis of teixobactin.

**Table 2:** MIC and MBC (in μg mL^–1^) of the teixobactin analogues **8**, **11–13**, **16** and daptomycin control against an extended panel of Gram positive bacteria. Strain information: MRSA 1: MRSA ATCC 700699, MRSA 2: MRSA DR 42412 (sputum), MRSA 3: MRSA DM21455 (eye). MRSA 2 and MRSA 3 are clinical isolates. *Staphylococcus aureus* ATCC 29213, *Enterococcus faecalis* (VRE 1: VRE ATCC 700802, VRE 2: VRE ATCC 29212). *M. smegmatis* ATCC 607. Culture media: Mueller Hinton broth

Strain		Compound						
		(**8**)	(**11**)	(**12**)	(**13**)	(**16**)	Vancomycin	Daptomycin
MRSA 1	MIC	4	1	0.25	0.25	1	2	0.5
	MBC	16	4	1	2	2	—	—
MRSA 2	MIC	1	0.5	≤0.0625	≤0.0625	0.125	2	0.5
	MBC	4	4	≤0.0625	≤0.0625	0.5	—	—
MRSA 3	MIC	1	0.25	≤0.0625	≤0.0625	0.5	2	0.5
	MBC	2	2	0.125	≤0.0625	1	—	—
*Staphylococcus aureus*	MIC	1	0.25	≤0.0625	≤0.0625	0.25	4	0.25
	MBC	2	1	0.125	0.125	1	—	
VRE 1	MIC	4	0.5	≤0.0625	0.25	2	>4	0.5
VRE 2	MIC	4	0.5	≤0.0625	0.25	2	>4	0.5
*M. smegmatis*	MIC	1–2	—	0.5	1	1–2	>64	—

### Time dependent killing of bacteria using teixobactin analogues **12** and **13**

**Fig. 4:**
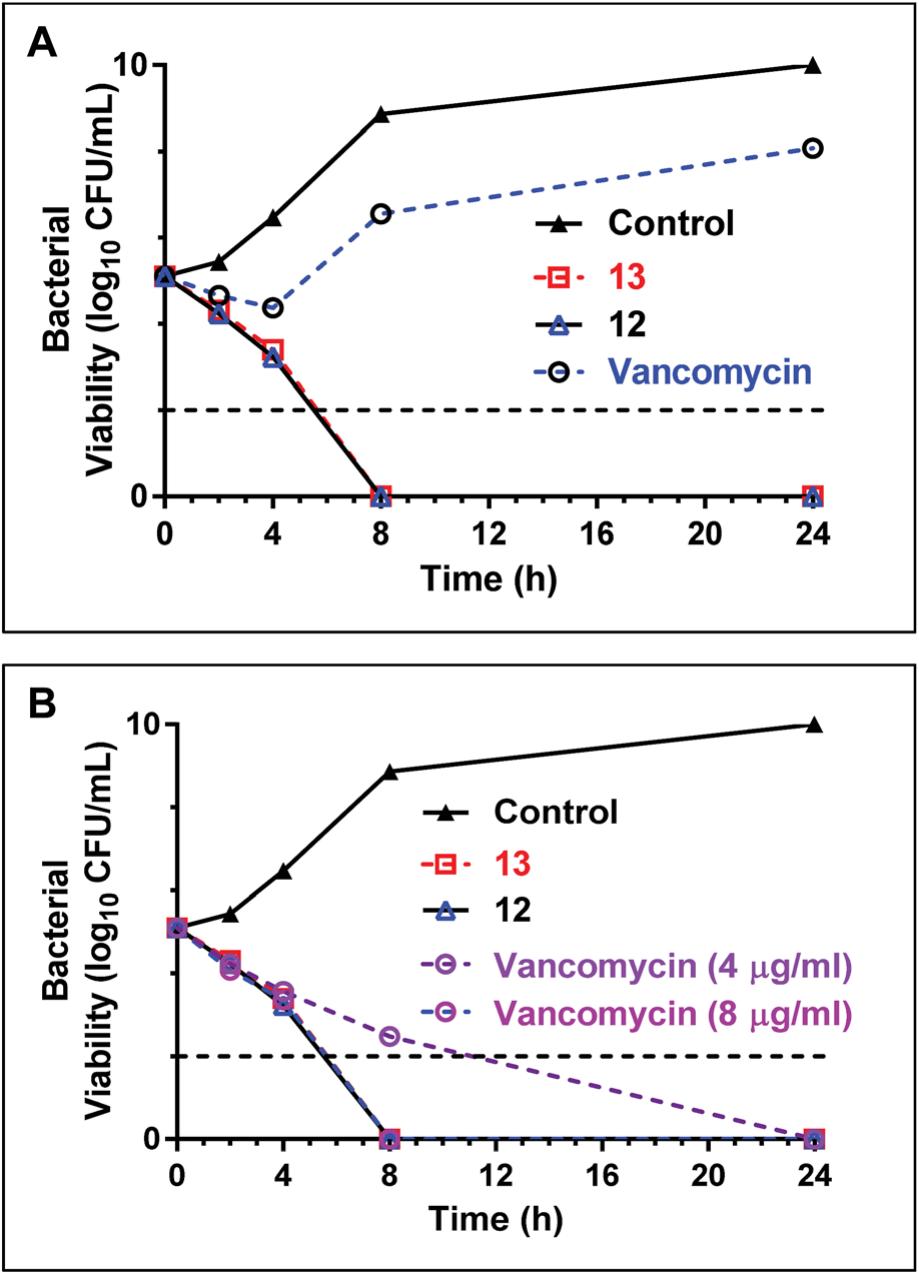
(A) Time-kill kinetics of teixobactin analogues **12** & **13** with a comparative antibiotic (vancomycin) against MRSA 21455. The concentration of teixobactin analogues and vancomycin are maintained at 0.5 μg mL^–1^. (B) Time-kill kinetics of teixobactin analogues with vancomycin against MRSA 21455 strains at elevated concentrations of the antibiotics. At 8 μg mL^–1^ concentration the kill kinetics profiles are similar for vancomycin & teixobactin analogues **12** & **13**. The horizontal dotted line represents the limit of detection.

Early stage time-kill kinetics for Ile_10_-teixobactin (**12**) and Leu_10_-teixobactin (**13**) against MRSA ATCC 21455 using vancomycin as a control were carried out as described (Page S53†).^2^ At 0.5 μg mL^–1^, both Ile_10_-teixobactin (**12**) and Leu_10_-teixobactin (**13**) were found to elicit complete bactericidal activity within 8 h whereas substantial growth was observed in the presence of vancomycin (0.5 μg mL^–1^, Fig. 4A). The concentration of vancomycin needs to be increased to 8 μg mL^–1^ in order to have similar effects as the teixobactin analogues **12** & **13** (Fig. 4B).

### Toxicity studies and haemolysis assay

**Fig. 5:**
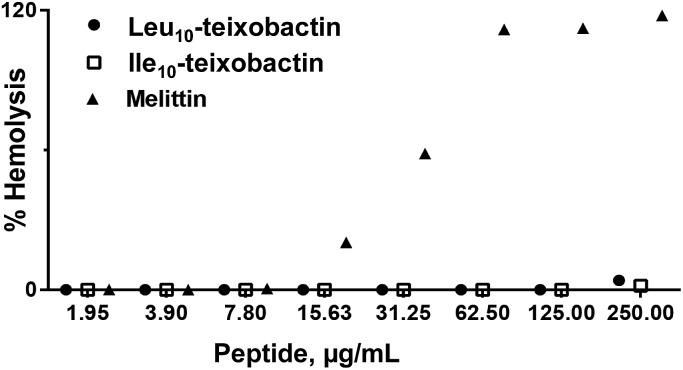
Hemolytic activity of teixobactin analogues **12** and **13** for rabbit erythrocytes. The blood cells were exposed to various concentrations of peptides for 1 h and the release of haemoglobin was determined spectrophotometrically. Each value represents an average of triplicate experiments.

The analogues Ala_10_-teixobactin (**8**), Val_10_-teixobactin (**11**) and Leu_10_-teixobactin (**13**) were tested on HeLa cell cultures and no significant toxicity was observed (relative survival 90–100%) up to a concentration of 100 μM (Fig. S79†) which is well above the MIC values (0.2–0.8 μM, 125–500 times). Additionally, a haemolytic assay using Leu_10_-teixobactin and Ile_10_-teixobactin against rabbit erythrocytes using melittin as a control (Fig. 5) indicated that peptides Leu_10_-teixobactin (**13**) and Ile_10_-teixobactin (**12**) did not show any discernible haemolytic activity, even at concentrations that exceed >500× the mean MIC values whereas substantial haemolytic activity was observed for melittin (Fig. 5). These results establish the non-haemolytic properties of the designed teixobactin analogues.

### Lipid II binding assay

To better understand the potent antibacterial activity of Ala_10_-teixobactin we have performed the lipid II TLC binding assay as reported earlier for teixobactin.^2^ Teixobactin and Arg_10_-teixobactin bind to lipid II in a 2 : 1 ratio resulting in the complete disappearance of the lipid II spot on TLC (Fig. S76†). Although Ala_10_-teixobactin also shows binding with lipid II in a 2 : 1 ratio, a small amount of lipid II was still visible on TLC. The lipid II spot, however, completely disappears by increasing the concentration of Ala_10_-teixobactin (Fig. S76†). TLC binding studies with Leu_10_-teixobactin also showed complete disappearance of the lipid II spot when a ratio of 2 : 1 was used (Fig. S78†). It is very interesting that Ala_10_-teixobactin and Leu_10_-teixobactin were able to bind to lipid II without having a cationic amino acid residue like l-*allo*-enduracididine/arginine off the cyclic peptide ring.

### Geranyl pyrophosphate (lipid II mimic) binding studies

**Fig. 6:**
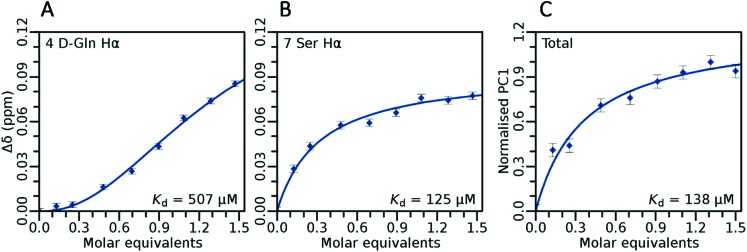
Selected binding isotherms obtained from titrations of geranyl pyrophosphate into Ala_10_-teixobactin demonstrating residue-specific binding behavior with cooperative characteristics. Error bars show RMS of function fit. PC: principal component.

In order to evaluate target binding, we have performed the lipid II TLC binding assay^2^ with Ala_10_-teixobactin. This assay provides qualitative binding data of Ala_10_-teixobactin with lipid II. Although the technique is fast and effective, the results obtained *via* this method do not necessarily reflect whole cell activities. This has been reported previously by us where both d and l derivatives of teixobactin were found to bind to lipid II but only the former was biologically active.^13^ Therefore, in order better understand the target binding of teixobactin analogues in a quantitative manner, extensive NMR studies (Fig. 6) on Ala_10_-teixobactin and geranyl pyrophosphate were performed. Geranyl pyrophosphate possesses a pyrophosphate and isoprenyl chain similar to lipid II making it suitable for solution phase NMR studies.

Both TLC (Fig. S77†) and NMR (Fig. 6, Table 3) suggest a 2 : 1 binding between Ala_10_-teixobactin and geranyl pyrophosphate. When titrating geranyl pyrophosphate into Ala_10_-teixobactin certain residues such as Ser_7_ were found to bind with classic Michaelis–Menton binding kinetics (Fig. 6B). However, some isotherms exhibited a sigmoidal shape (Fig. 6A). This can occur due to intermediate exchange on the NMR time-scale, and therefore cooperative binding is not an obvious choice. However, given our initial TLC data which shows a binding of 2 : 1 we have fitted the sigmoidal data using the Hill coefficient. It was found that all N-terminal residues weakly bound (*K*_D_ ∼ 0.5 mM) geranyl pyrophosphate in a highly cooperative (Hill coefficient ∼ 2) manner, whereas ring-proximal residues bound significantly tighter but less cooperatively. Tightest binding was observed for Ser_7_ (*K*_D_ ∼ 125 μM), which in a recently published teixobactin X-ray structure^12^ points its hydroxyl directly towards a bound anion. Analysing the overall binding using PCA (Fig. 6C), which removes any influence of intermediate exchange from the isotherms,^21^ gave a net *K*_D_ of ∼138 μM.

**Table 3:** Dissociation constants between Ala_10_-teixobactin and geranyl pyrophosphate at residue resolution, as determined by NMR titration. A blank Hill coefficient indicates Michaelis–Menton binding kinetics was sufficient to satisfactorily describe the titration data

Entry	*K*_D_ (μM)	Hill coefficient
1 Me-d-Phe Hα	n.d.	n.d.
2 Ile Hα	348 ± 18	2.1
3 Ser Hα	503 ± 8	2.0
4 d-Gln Hα	507 ± 2	2.2
5 d-Ile Hα	483 ± 4	1.7
6 Ile Hα	n.d.	n.d.
7 Ser Hα	125 ± 3	
8 d-Thr Hα	204 ± 3	
9 Ala Hα	394 ± 4	2.1
10 Ala Hα	314 ± 3	
11 Ile Hα	391 ± 4	1.5
**Net**	**138 ± 5**	

**Fig. 7:**
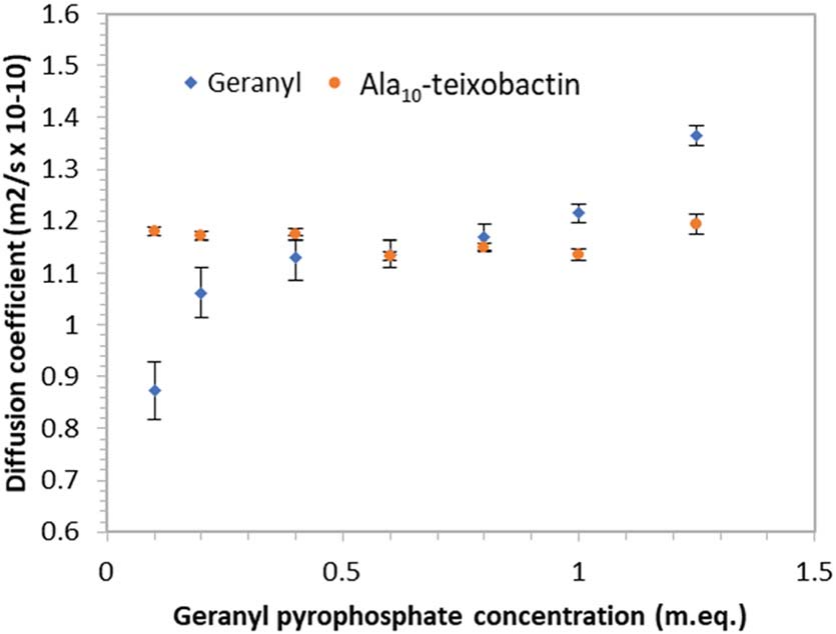
Graph of diffusion co-efficient *vs.* concentration of geranyl pyrophosphate indicating teixobactin does not aggregate when exposed to increasing geranyl pyrophosphate concentrations. Error bars indicate standard deviation of the fitting function.

In order to determine if teixobactin aggregates in the presence of geranyl pyrophosphate ^1^H DOSY (diffusion ordered spectroscopy) spectra were recorded at each titration point and the diffusion coefficients calculated for both geranyl pyrophosphate and teixobactin (Fig. 7). Over the course of the titration the diffusion coefficient obtained from Ala_10_-teixobactin remained constant, indicating no aggregation occurred. The diffusion coefficient observed for geranyl pyrophosphate increased slightly over the course of the titration, indicating that it may have adopted a more compact structure upon association with the teixobactin.

### Antagonization assay

In order to further prove a lipid II mediated mode of action, an antagonization assay was performed using Leu_10_-teixobactin (**13**) with lipid II as described in literature.^2^ The ratios of Leu_10_-teixobactin to lipid II tested were 1 : 0.5, 1 : 1, 1 : 2 and 1 : 5 and growth was observed. These results are consistent with the 2 : 1 binding ratio observed using the TLC assay (Table S3†). However in case of Leu_10_-teixobactin (control), no growth was observed.

### Structural studies using NMR

**Fig. 8:**
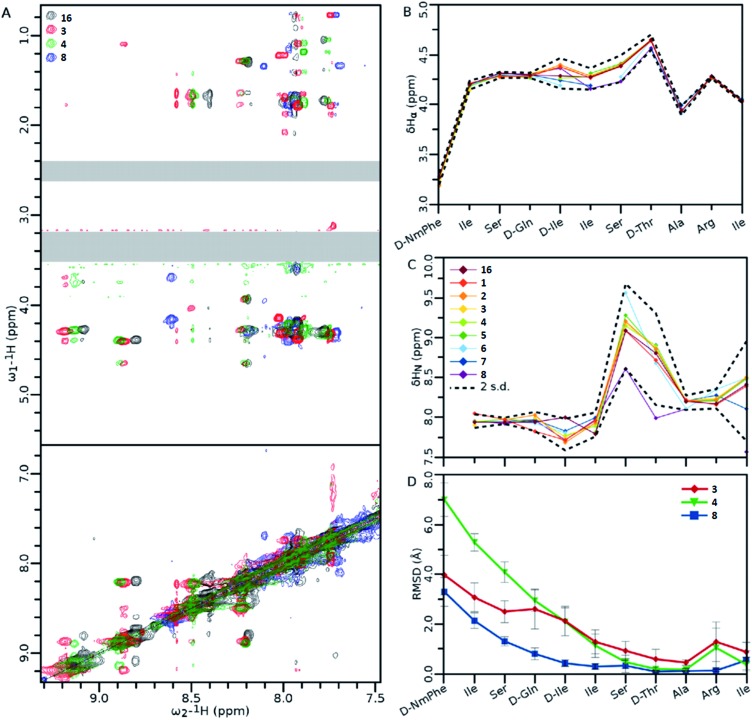
(A) Overlay of the amide fingerprint regions of the 1H–1H NOESY spectra of wild type (Arg_10_-teixobactin) and active teixobactin analogues. (B and C) Chemical shift data obtained from α (B) and amide (C) protons. (D) Statistics of structures calculated using the NOEs obtained from panel (A). For clarity, the DMSO (∼3.3 ppm) and residual water (∼2.5 ppm) signals have been obscured with grey boxes. Data show the average RMSD of each atom in the residue from all 20 members of the ensemble. Error bars are standard deviation in the RMSDs of each residue's atoms. Standard deviations were calculated including the chemical shifts for **16**.^11^ Ensembles of **20** structures generated by Cyana 2.1 22 and refined in Gromacs.^23^ Error bars indicate standard deviation amongst all the atoms of that residue. Spectra were recorded on 1 mM teixobactin samples dissolved in DMSO-d_6_ on a 500 MHz spectrometer at 300 K. Legends for 8B and C are the same, and are shown in panel (C).

NMR analysis of teixobactin analogues (Fig. 8) reveals common structural characteristics between those analogues which retain some residual antibacterial activity. Ala_10_-teixobactin was chosen for NMR studies as it provides the most direct comparison with other analogues. All analogues retain most of the NOEs observed in the Arg_10_-teixobactin, despite some differences in amide chemical shift (Fig. 8A). From Fig. 8B and C it can be observed that α proton chemical shifts show little variation between analogues at both termini: *N*-terminal similarities are likely due to these residues existing in a random coil environment; *C*-terminal similarities are likely due to the restraints placed upon these residues by the ring structure. Amide chemical shifts are more variable, particularly for residues **7** and **8**, in which the chemical shift of these protons is ∼1 ppm downfield in Ala_10_-teixobactin (**8**). This is likely due to the loss of the guanidinium group, and suggests proximity between these residues and Arg_10_. The *N*-terminus again shows little variation, characteristic of a random coil. The mutated residue chemical shifts were excluded from the statistics. Fig. 8D shows that in all three mutants Ala_3_-Arg_10_-teixobactin (**3**), Ala_4_-Arg_10_-teixobactin (**4**) and Ala_10_-teixobactin (**8**) the *N*-termini were unstructured, but were showing evidence of structure starting approximately from residue **5**, where in all cases the RMSD had dropped by ∼50% from that observed at their termini. The RMSDs observed at the C termini are low, as this area is highly constrained in structure by the ring.

## Conclusion

In conclusion, we have described a unique design and rapid synthesis of several highly potent teixobactin analogues by replacing the synthetically challenging amino acid l-*allo*-enduracididine with commercially available non-polar residues such as leucine (**13**) and isoleucine (**12**). The teixobactin analogues from this work have shown highly potent antibacterial activity against a broad panel of MRSA, MSSA and VRE, despite their simpler design. Early stage kill kinetics data suggests Leu_10_-teixobactin and Ile_10_-teixobactin to be superior to vancomycin against MRSA. An antagonization assay suggests a lipid II mediated mode of action for Leu_10_-teixobactin. Most importantly, contrary to the current understanding we have demonstrated that cationic amino acids such as L-*allo*-enduracididine, arginine or lysine at position 10 are not essential for target (lipid II) binding and antibacterial activity. This surprising finding opens the door to the design and synthesis of several highly simplified potent teixobactin analogues and challenges many of the current assumptions about the mechanism of action of teixobactin. Our design of highly potent teixobactin analogues has several advantages such as improved yields ∼10–20%, ease of synthesis (including 10 min μwave assisted coupling steps and a 30 min cyclisation step) and uses commercially available building blocks.

NMR studies reveal that the analogues Ala_3_-Arg_10_-teixobactin (**3**), Ala_4_-Arg_10_-teixobactin (**4**) and Ala_10_-teixobactin (**8**) are more unstructured towards the N-termini but highly structured towards the C termini due to the close-by ring. We have performed qualitative lipid II binding experiments and measured the binding affinities of individual amino acid residues of Ala_10_-teixobactin and geranyl pyrophosphate (lipid II mimic) by NMR to understand the role of amino acid residues in binding. Ser_7_ was found to have the tightest binding with an experimental *K*_D_ of 125 μM.

To the best of our knowledge, Ile_10_-teixobactin (**12**) and Leu_10_-teixobactin (**13**) are the only reported teixobactin analogues which have shown superior potency against resistant Gram positive bacteria. The results from this work represent a significant advancement in our current understanding of the residues critical to the biological activity of teixobactin and associated analogues. We anticipate that our design and relatively rapid synthesis will help overcome current challenges in the field. As it stands, our work herein provides ready access to highly potent teixobactin analogues and will enable the development of teixobactin analogues with drug like properties against resistant bacterial strains. The findings presented in this work have broad implications and are expected to facilitate the development of peptide based antibiotics for combatting the serious global challenges posed by AMR.

## Funding sources

Anish Parmar, Abhishek Iyer and Charlotte S. Vincent would like to thank the University of Lincoln for funding. Edward Taylor would like to thank the Royal Society for their kind support (grant number UF100116). Ishwar Singh would like to acknowledge the Royal Society for their kind support (grant number (RG130163) and Horizon 2020 (645684)). Daniel Lloyd would like to acknowledge the Rosetrees trust for their kind support (grant number JS16/M583). Timea Palmai-Pallag and Csanad Bachrati are funded by the BBSRC grant BB/K019597/1.

## Conflicts of interest

The authors declare no conflict of interest.

## Supplementary Material

Supplementary information
