# Supplementary material for: Teixobactin analogues reveal enduracididine to be non-essential for highly potent antibacterial activity and lipid II binding
Source: Chem Sci. 2017 Oct 5;8(12):8183–92. doi: 10.1039/c7sc03241b (PMC5855875; doi:10.1039/c7sc03241b)
Supplement: Supplementary file 1 [file SC-008-C7SC03241B-s001.pdf]

Electronic Supplementary Material (ESI) for Chemical Science.
This journal is © The Royal Society of Chemistry 2017

Supplementary Information

FOR

New teixobactin analogues reveal enduracididine to be non-essential for potent antibacterial activity and lipid II binding

Anish Parmar,[a ‡] Abhishek Iyer,[a,b ‡] Stephen H. Prior,[c] Daniel G. Lloyd,[d] Eunice Tze Leng Goh,[e] Charlotte S. Vincent,[d] Timea Palmai-Pallag,[d] Csanad Z. Bachrati,[d] Eefjan Breukink,[f] Annemieke Madder,[b] Rajamani Lakshminarayanan[f], Edward J. Taylor[d] and Ishwar Singh[a] *

[a.] School of Pharmacy, JBL Building, University of Lincoln, Beevor St. Lincoln LN67DL, UK, e-mail: isingh@lincoln.ac.uk

[b.] Organic and Biomimetic Chemistry Research Group, Department of Organic and Macromolecular Chemistry, Ghent University, Krijgslaan 281 (S4), B-9000 Ghent, Belgium

[c.] School of Chemistry, JBL Building, University of Lincoln, Beevor St. Lincoln LN67DL, UK

[d.] School of Life Sciences, JBL Building, University of Lincoln, Beevor St. Lincoln LN67DL, UK

[e.] Singapore Eye Research Institute, The Academia, Discovery Tower Level 6, 20 College Road Singapore 169857

[f.] Department of Membrane Biochemistry and Biophysics, Institute of Biomembranes, Utrecht University, Padualaan 8, 3584 CH Utrecht, The Netherlands

TABLE OF CONTENTS

Sr. No.	Contents	Page Number
I	Materials	S2
II	Equipment used for the analysis and purification of compounds	S2
III	Syntheses of teixobactin analogues	S3
IV	HPLC/LC-MS analysis	S5
V	NMR analysis	S34
VI	MIC testing (screening)	S53
VII	Antagonization assay	S53
VIII	MIC testing (extended panel)	S53
IX	Time dependent killing of bacteria by teixobactin analogues 12 and 13	S55
X	Complex formation of teixobactin with lipid II and geranyl pyrophosphate	S55
XI	Cytotoxicity Assay	S57
XII	Haemolytic assay protocol	S57
XIII	References	S58

I. Materials

All L amino acids, Fmoc-D-Ala-OH Fmoc-D-Gln(Trt)-OH, Boc-N-methyl-D-phenylalanine, 1-[Bis(dimethylamino)methylene]-1H-1,2,3-triazolo[4,5-b]pyridinium3-oxidhexafluorophosphate (HATU), Phenylsilane ($PhSiH_3$), Tetrakis(triphenylphosphine)palladium(0) [$Pd(PPh_3)$], Diisoproplycarbodiimide (DIC) and Triisopropylsilane (TIS) were purchased from Fluorochem, UK. Fmoc-D-*allo*-Ile-OH and oxyma pure were purchased from Merck Millipore. The side chain protecting groups for the amino acids are ^{t}Bu for Ser, Pbf for Arg and Trt for Gln and Thr unless specified otherwise. Diisopropylethylamine (DIPEA), supplied as extra dry, redistilled, 99.5 % pure, Acetic anhydride, allyl chloroformate, $CDCl_3$ and polysorbate 80 and were purchased from Sigma Aldrich. Tritylchloride and 4-(Dimethylamino)pyridine were purchased from Alfa Aesar. Dimmethylformamide (DMF) peptide synthesis grade was purchased from Rathburn chemicals. Triethylamine, Diethyl ether (Et_2O), Dimethylsulfoxide (DMSO), Dichloromethane (DCM), Tetrahydrofuran (extra dry with molecular sieves), Formic acid 98-100% purity and Acetonitrile (HPLC grade) were purchased from Fisher Scientific. Water with the Milli-Q grade standard was obtained in-house from an ELGA Purelab Flex system. 2-Chlorotritylchloride resin (manufacturer's loading: 1.20 mmol/g) was purchased from Fluorochem. All chemicals were used without further purification. Geranyl pyrophosphate ammonium salt, 1 mg/mL in MeOH was purchased from Sigma Aldrich.

II. Equipment used for the analysis and purification of compounds

All peptides were analysed on a Thermo Scientific Dionex Ultimate 3000 RP-HPLC equipped with a Phenomenex Gemini NX C18 110 Å (150 x 4.6 mm) column using the following buffer systems: A: 0.1% HCOOH in milliQ water. B: ACN using a flow rate of 1 ml/min. The column was flushed with 95% A for 5 min prior to an injection and was flushed for 5 min with 95% B and 5% A after the run was finished.

Peptides were dissolved in (1:1) 0.1% HCOOH buffer in water and acetonitrile (ACN) and analysed using the following gradient: 95% A for 2 min. 5-95% B in 25 min. 95% B for 5 min. 5% A for 4 min.

Peptides were dissolved in 0.1% HCOOH buffer in water and in ACN (10-30% ACN) and purified using the same gradient as mentioned above, on a Thermo Scientific Dionex Ultimate 3000 RP-HPLC with a flow rate of 5 mL/min using a Phenomenex Gemini NX C18 110 Å (150 x 10 mm) semi-prep column.

LC-MS data were collected on an Agilent 1100 Series instrument with a Phenomenex Kinetex C18 100Å column (150 x 4.6 mm, 5 µm at 35 °C) connected to an ESMSD type VL mass detector with a flow rate of 1.5 ml/min was used with the following solvent systems: (A): 0.1% HCOOH in H_2O and (B) MeCN. The column was flushed with 100% A for 2 min, then a gradient from 0 to 100% B over 6 min was used, followed by 2 min of flushing with 100% B.

NMR spectra were recorded on a Bruker AV 500 NMR. HRMS spectra were recorded on a Thermo Scientific Q Exactive Plus Orbitrap Mass Spectrometer in the positive ion mode.

III. Syntheses of teixobactin analogues

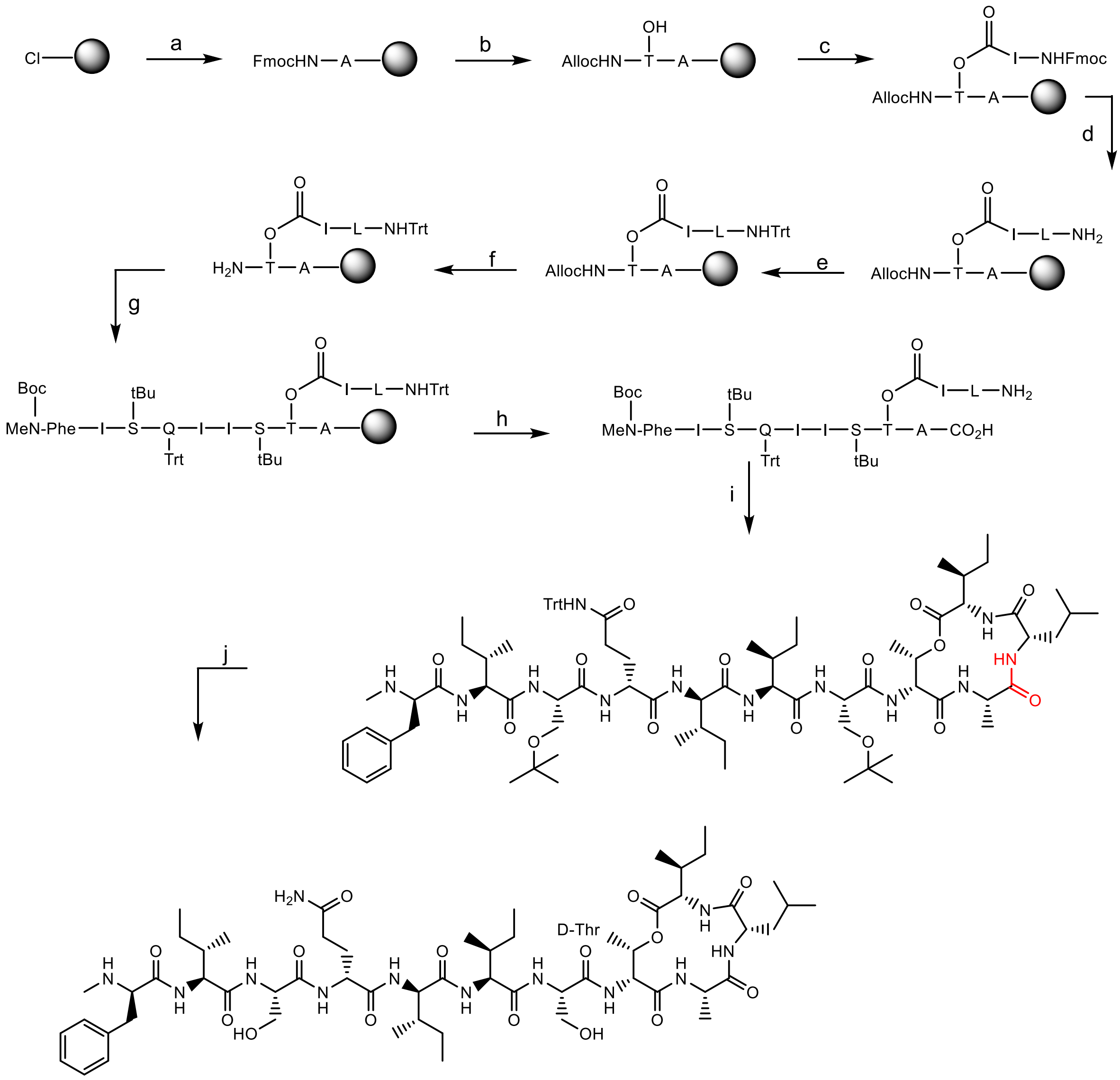

Fig. S1: Synthesis of Leu$_{10}$-teixobactin

(step a) Commercially available 2-Chlorotrityl chloride resin (manufacturer's loading = 1.2 mmol/g, 170 mg resin) was swelled in DCM in a reactor. To this resin was added 4 eq. Fmoc-Ala-OH/8 eq. DIPEA in DCM and the reactor was shaken for 3h. The loading determined by UV absorption of the piperidine-dibenzofulvene adduct was calculated to be 0.6 mmol/g, (170mg resin, 0.102 mmol). Any unreacted resin was capped with MeOH:DIPEA:DCM = 1:2:7 by shaking for 1h. (step b) The Fmoc protecting group was deprotected using 20% piperdine in DMF by shaking for 3 min, followed by draining and shaking again with 20% piperidine in DMF for 10 min. AllocHN-D-Thr-OH was then coupled to the resin by adding 3 eq. of the AA, 3 eq. HATU and 6 eq. DIPEA in DMF and shaking for 1.5h at room temperature. (step c) Esterification was performed using 10 eq. of Fmoc-Ile-OH, 10 eq. DIC and 5 mol% DMAP in DCM and shaking the reaction for 2h. This was followed by capping the unreacted alcohol using 10% Ac_2O/DIPEA in DMF shaking for 30 min and Fmoc was removed using protocol described earlier in step (b). (step d) Fmoc-Leu-OH was coupled using 4 eq. of AA, 4 eq. HATU and 8 eq. DIPEA in DMF and shaking for 1h followed by Fmoc deprotection using 20% piperidine in DMF as described earlier. (step e) The N terminus of Leu was protected using 10 eq. Trt-

Cl and 15% Et_3N in DCM and shaking for 1h. The protection was verified by the Ninhydrin colour test. (step f) The Alloc protecting group of D-Thr was removed using 0.2 eq. $[Pd(PPh^3)]^0$ and 24 eq. $PhSiH_3$ in dry DCM under argon for 20 min. This procedure was repeated again increasing the time to 45 min and the resin was washed thoroughly with DCM and DMF to remove any Pd stuck to the resin. (step g) All amino acids were coupled using 4 eq. Amino Acid, 4 eq. DIC/Oxyma using a microwave peptide synthesizer. Coupling time was 10 min. Deprotection cycles were performed as described earlier. (step h) The peptide was cleaved from the resin without cleaving off the protecting groups of the amino acid side chains using TFA:TIS:DCM = 2:5:93 and shaking for 1h. (step i) The solvent was evaporated and the peptide was redissolved in DMF to which 1 eq. HATU and 10 eq. DIPEA were added and the reaction was stirred for 30 min to perform the cyclization. (step j) The side-chain protecting groups were then cleaved off using TFA:TIS:H_2O = 95:2.5:2.5 by stirring for 1h. The peptide was precipitated using cold Et_2O (-20°C) and centrifuging at 7000 rpm to obtain a white solid. This solid was further purified using RP-HPLC using the protocols described previously[1].

All other teixobactin analogues were synthesised according to the above procedure.

**Sr No.**	**Compound Number**	**Name**	**Chemical formula**	**Exact Mass**	**Mass found [M + H⁺]**	**Overall yield [%]**
1	1	Ac-D-Ala_1-Arg_{10}-texiobactin	$C_{53}H_{93}N_{15}O_{16}$	1195.69	1196.4	12
2	2	Ala_2-Arg_{10}-teixobactin	$C_{55}H_{91}N_{15}O_{15}$	1201.68	1202.4	10
3	3	Ala_3-Arg_{10}-teixobactin	$C_{58}H_{97}N_{15}O_{14}$	1227.73	1228.5	11
4	4	D-Ala_4-Arg_{10}-teixobactin	$C_{56}H_{94}N_{14}O_{14}$	1186.71	1187.4	12
5	5	D-Ala_5-Arg_{10}-teixobactin	$C_{55}H_{91}N_{15}O_{15}$	1201.68	1202.4	11
6	6	Ala_6-Arg_{10}-teixobactin	$C_{55}H_{91}N_{15}O_{15}$	1201.68	1202.4	12
7	7	Ala_7-Arg_{10}-teixobactin	$C_{58}H_{97}N_{15}O_{14}$	1227.73	1228.5	12
8	8	Ala_{10}-teixobactin	$C_{55}H_{90}N_{12}O_{15}$	1158.66	1159.5	13
9	9	D-Ala_{10}-teixobactin	$C_{55}H_{90}N_{12}O_{15}$	1158.66	1159.5	10
10	10	Gly_{10}-teixobactin	$C_{54}H_{88}N_{12}O_{15}$	1144.65	1145.6	18*
11	11	Val_{10}-teixobactin	$C_{57}H_{94}N_{12}O_{15}$	1186.70	1187.6	24
12	12	Ile_{10}-teixobactin	$C_{58}H_{96}N_{12}O_{15}$	1200.71	1201.5	10
13	13	Leu_{10}-teixobactin	$C_{58}H_{96}N_{12}O_{15}$	1200.71	1201.5	20
14	14	Ser_{10}-teixobactin	$C_{55}H_{90}N_{12}O_{16}$	1174.66	1175.6	21
15	15	Phe_{10}-teixobactin	$C_{61}H_{94}N_{12}O_{15}$	1235.6962	1235.7040 [M]	12

Table S1: Compound number, code, exact mass, chemical formula, mass found and overall yields for compounds **1-15**.

***** Gly_{10}-teixobactin afforded a yield of 2% when synthesised for the first time possibly due to deletion sequences. Since the yield was unusually low, therefore, the synthesis was repeated a second time thereby affording a yield of 18%.

IV. HPLC/LC-MS analysis

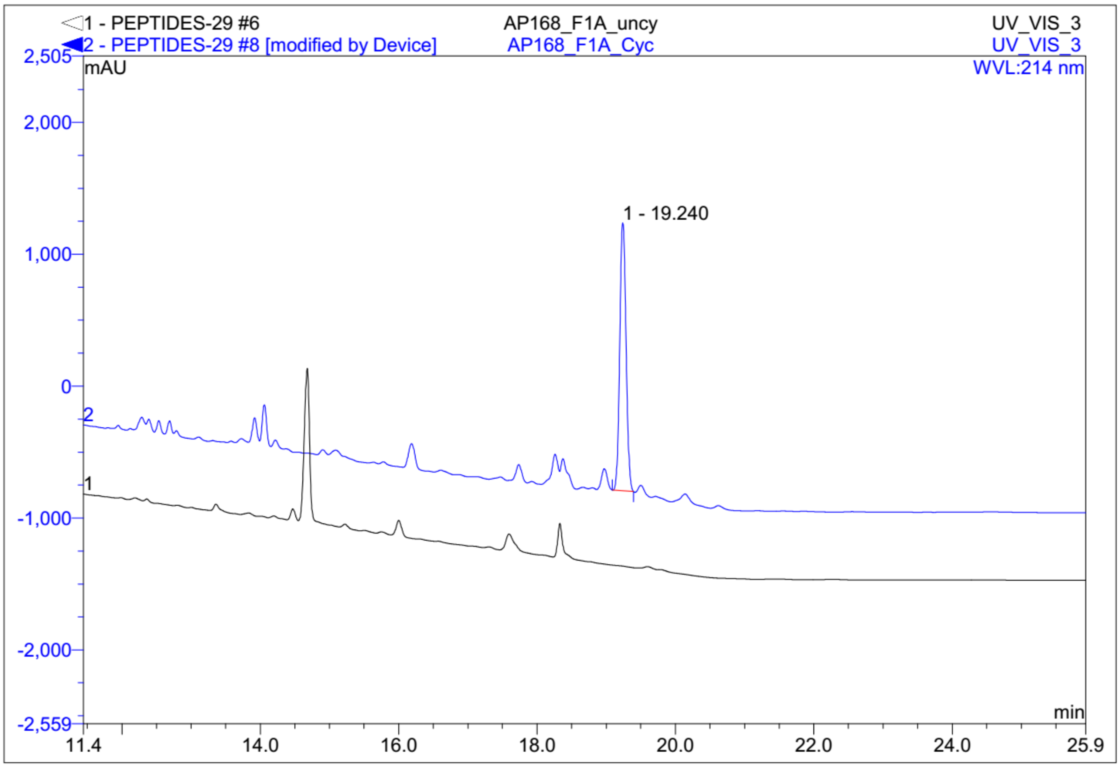

Fig. S2: HPLC trace showing the progress of cyclisation reaction for the synthesis of analogue **1** (i): conversion of the uncyclized protected teixobactin analogue t_R= 14.677 min (shown in black) to the cyclized protected teixobactin analogue t_R = 19.240 min (shown in blue) (Gradient: 5-95% in 25 min)

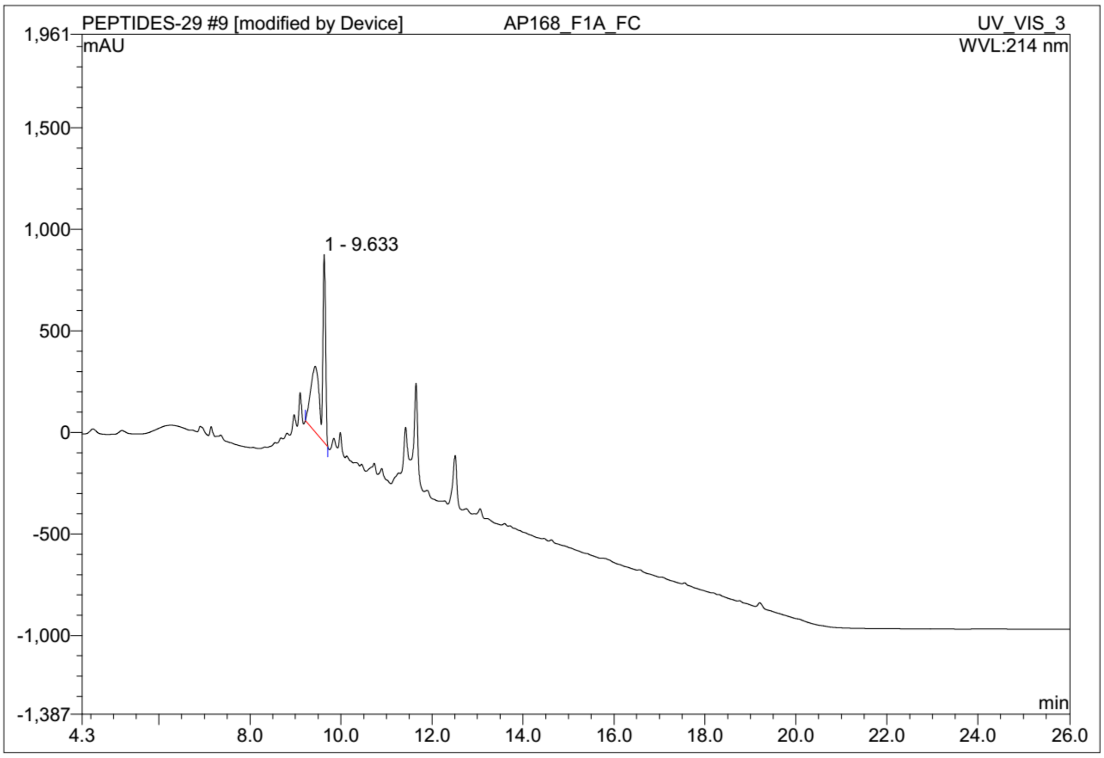

Fig. S3: HPLC trace of crude teixobactin analogue **1** t_R = 9.633 min (gradient: 5-95% ACN in 25 min using A: 0.1% HCOOH in water, B: ACN)

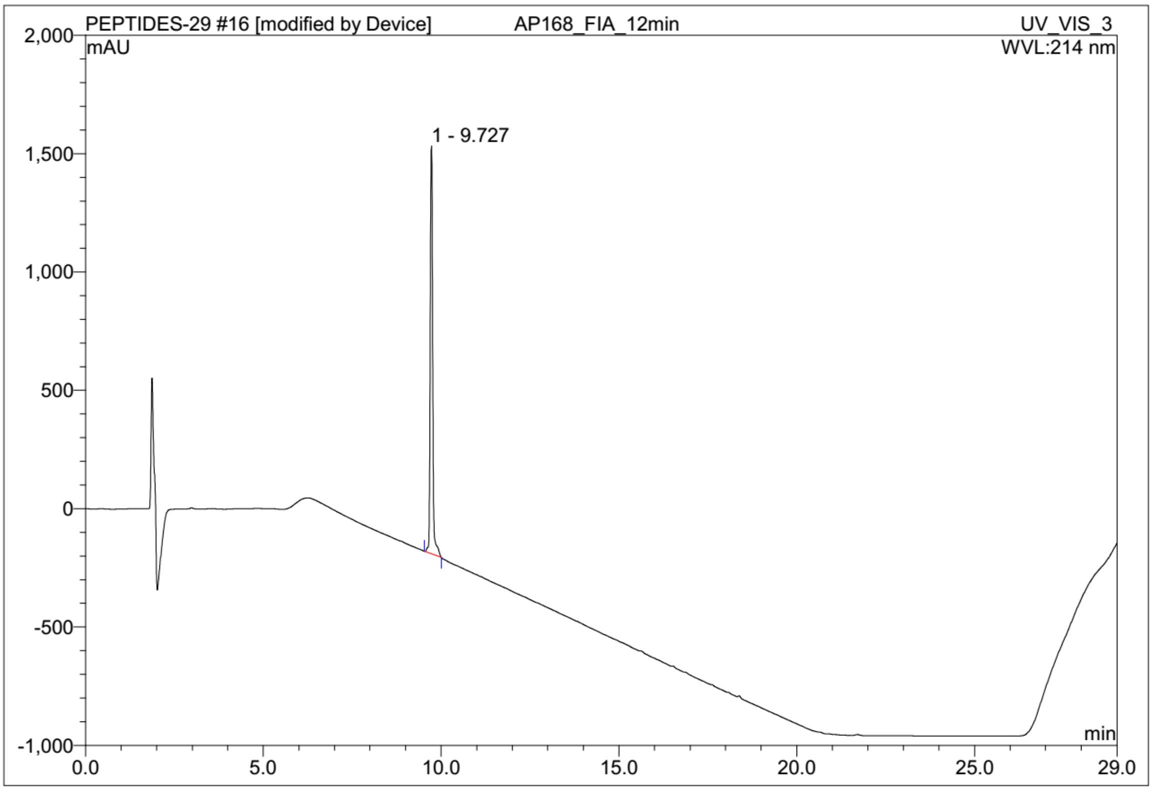

Fig. S4: HPLC trace of HPLC purified teixobactin analogue **1** (gradient: 5-95% ACN in 25 min using A: 0.1% HCOOH in water, B: ACN)

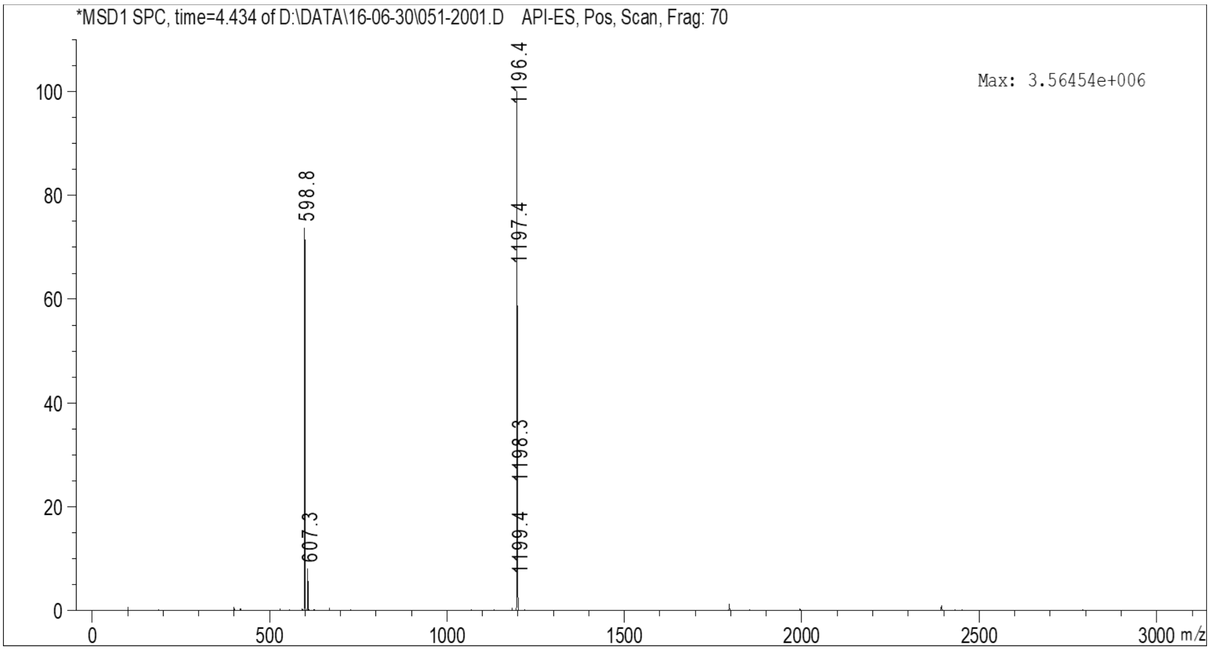

Fig. S5: ESI-MS from LC-MS of HPLC purified teixobactin analogue **1**. Exact mass calcd. for $C_{53}H_{93}N_{15}O_{16}$ = 1195.69, found M + H^+ = 1196.4, M/2 + H^+ = 598.8.

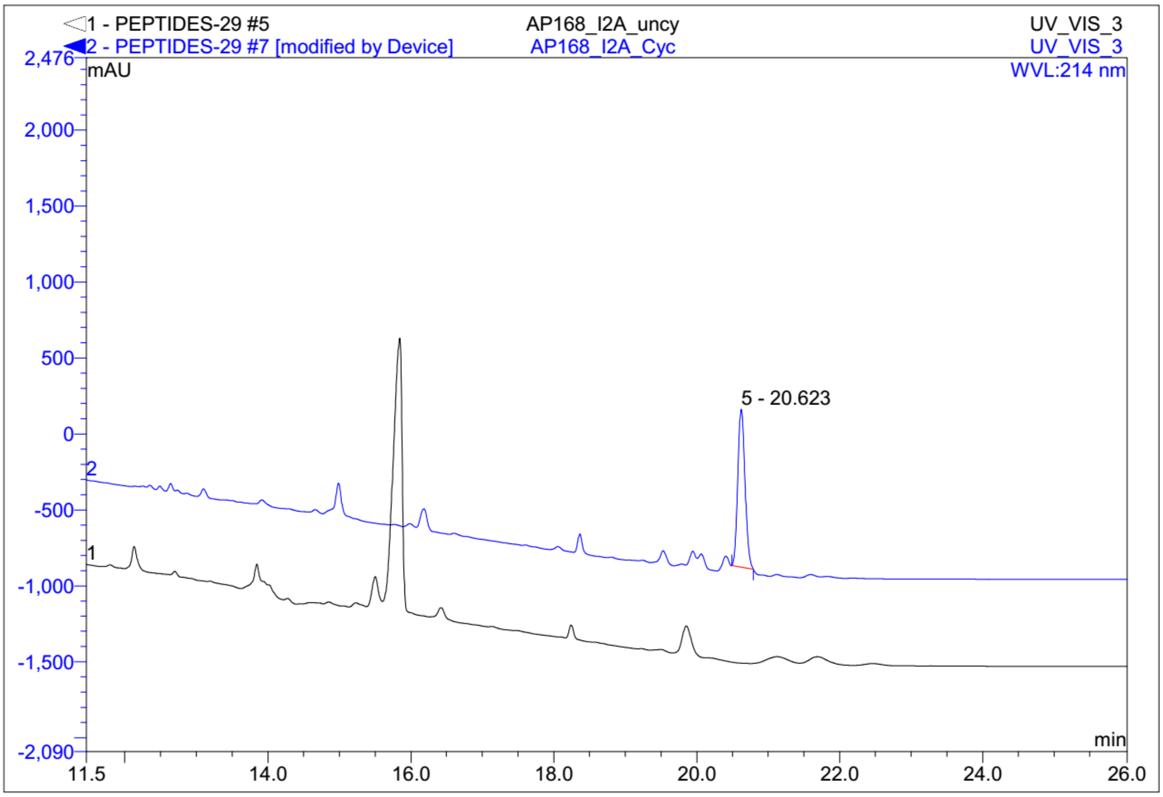

Fig. S6: HPLC trace showing the progress of cyclisation reaction for the synthesis of analogue **2** (i): conversion of the uncyclized protected teixobactin analogue t_R= 15.843 min (shown in black) to the cyclized protected teixobactin analogue t_R = 20.623 min (shown in blue) (Gradient: 5-95% in 25 min)

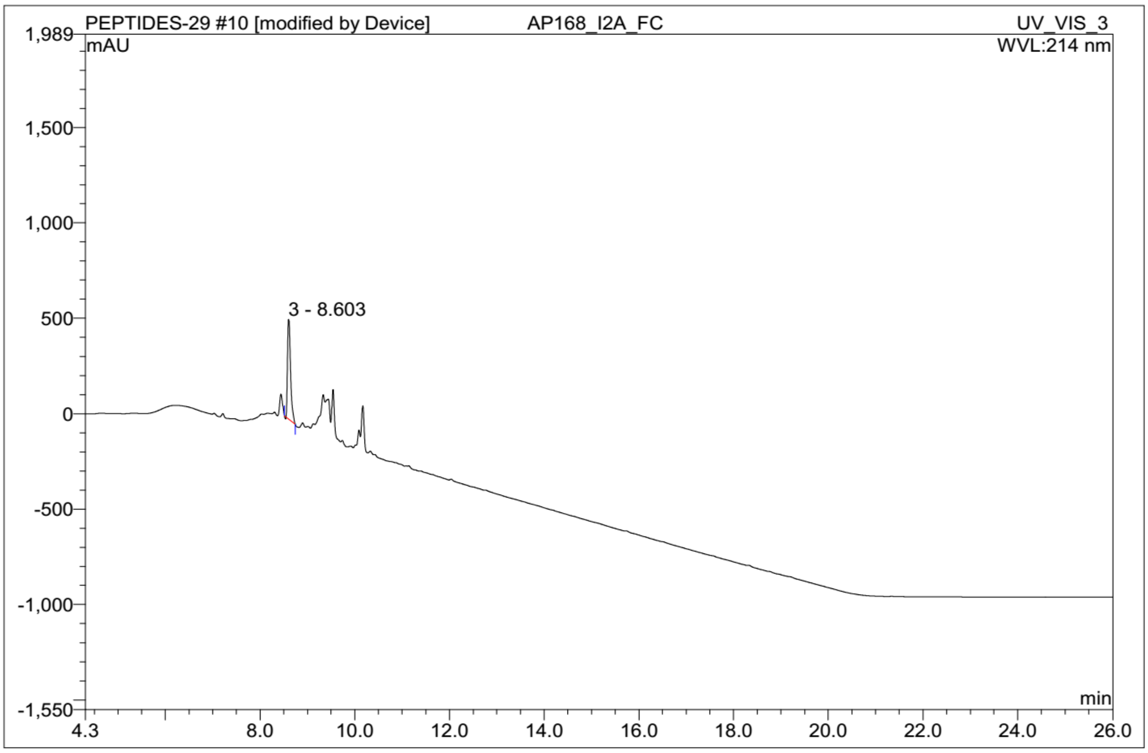

Fig. S7: HPLC trace of crude teixobactin analogue **2** t_R = 8.603 min (gradient: 5-95% ACN in 25 min using A: 0.1% HCOOH in water, B: ACN)

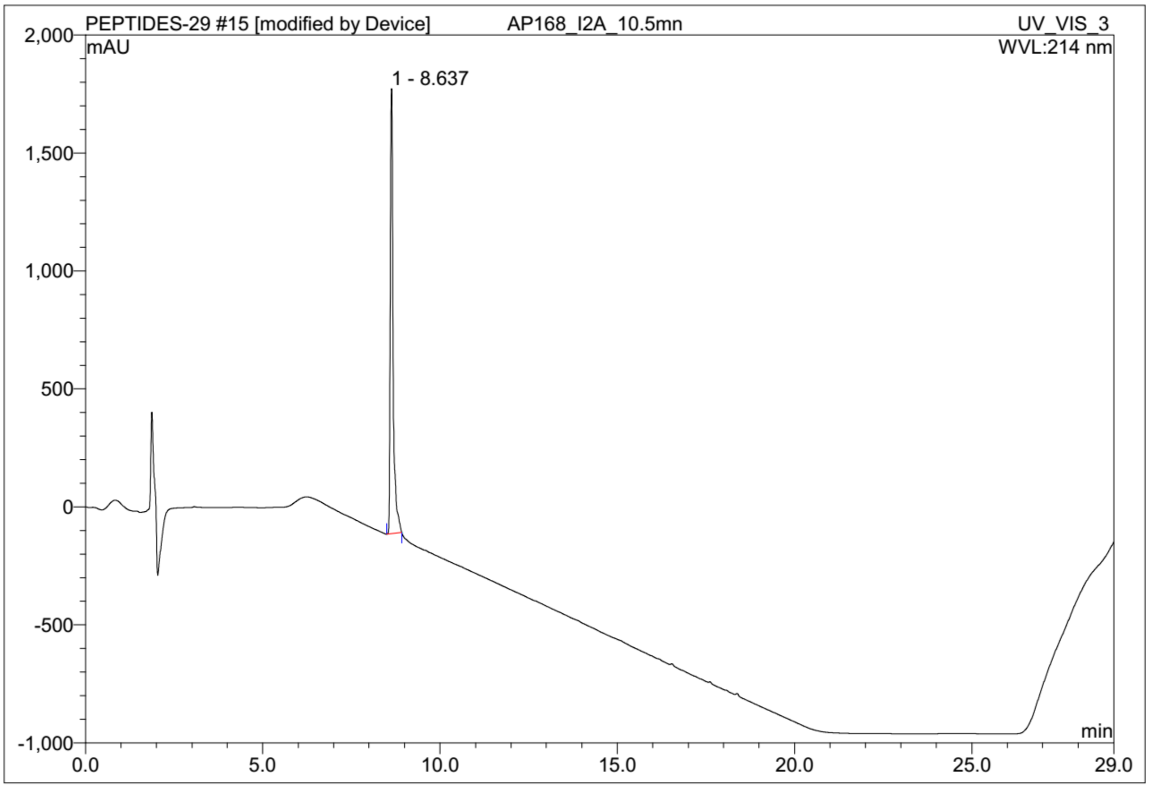

Fig. S8: HPLC trace of HPLC purified teixobactin analogue **2** (gradient: 5-95% ACN in 25 min using A: 0.1% HCOOH in water, B: ACN)

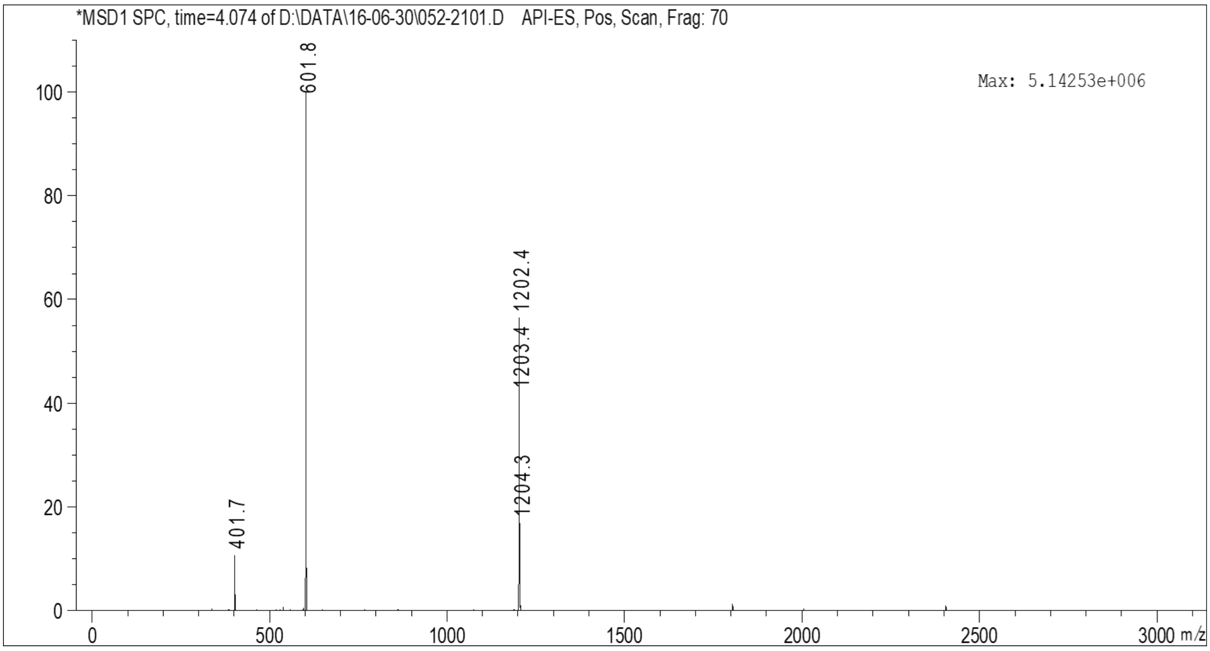

Fig. S9: ESI-MS from LC-MS of HPLC purified teixobactin analogue **2**. Exact mass calcd. for $C_{55}H_{91}N_{15}O_{15}$ = 1201.68, found M + H^+ = 1202.4, M/2 + H^+ = 601.8, M/3 + H^+ = 401.7.

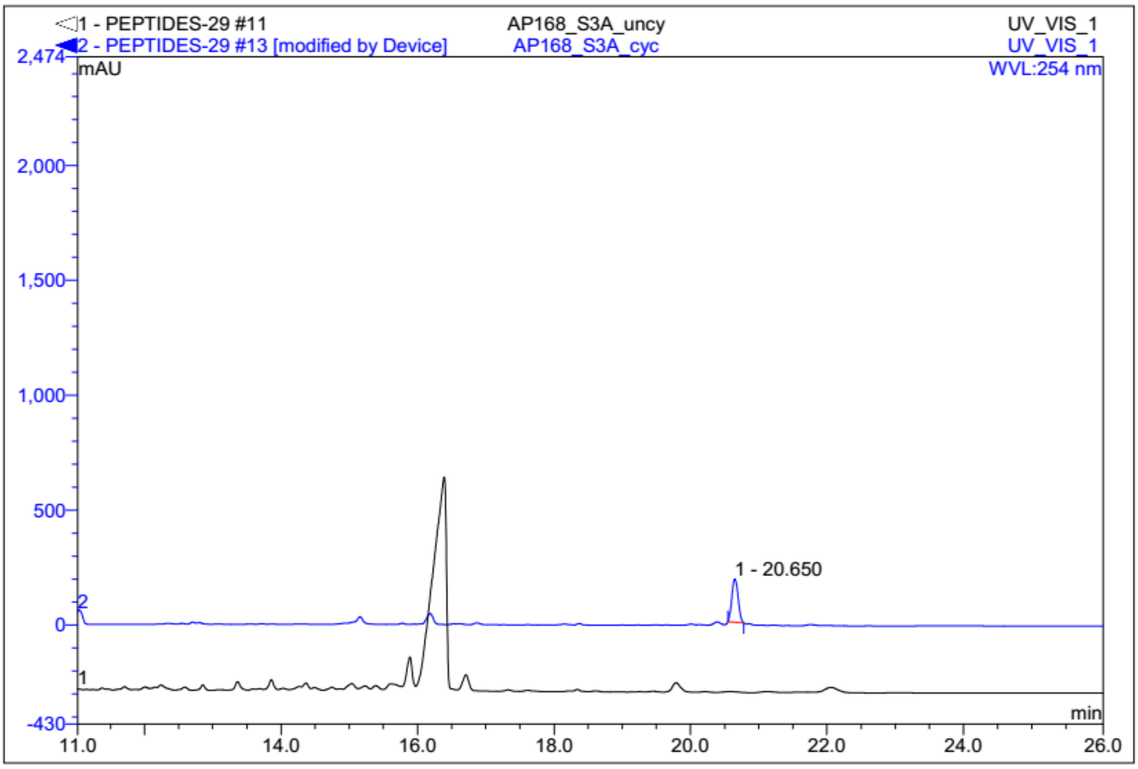

Fig. S10: HPLC trace showing the progress of cyclisation reaction for the synthesis of analogue **3** (i): conversion of the uncyclized protected teixobactin analogue t_R= 16.390 min (shown in black) to the cyclized protected teixobactin analogue t_R = 20.650 min (shown in blue) (Gradient: 5-95% in 25 min)

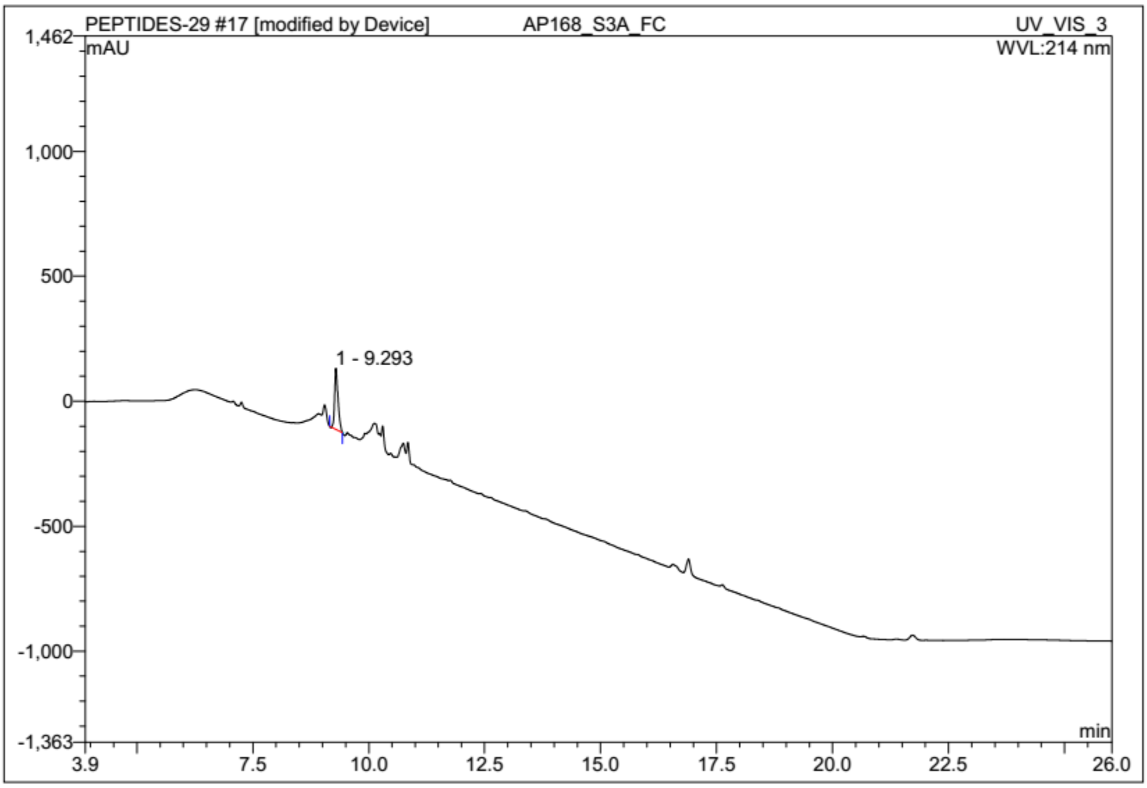

Fig. S11: HPLC trace of crude teixobactin analogue **3** t_R = 9.293 min (gradient: 5-95% ACN in 25 min using A: 0.1% HCOOH in water, B: ACN)

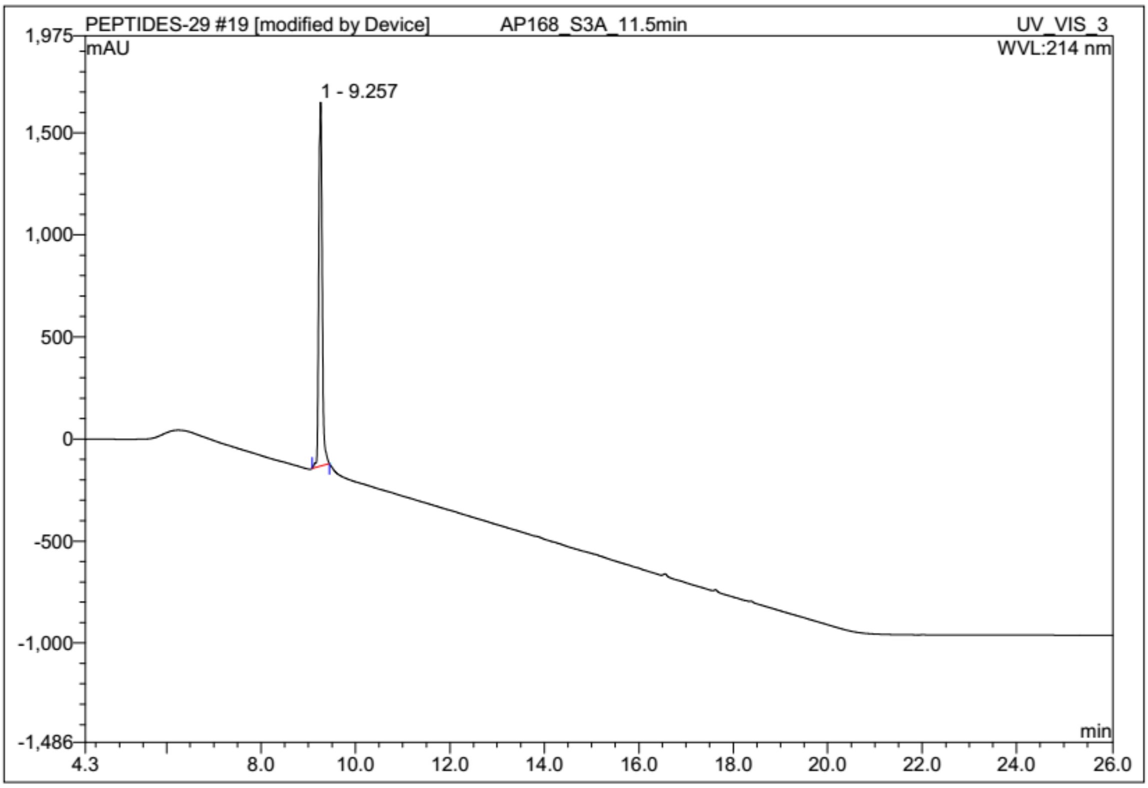

Fig. S12: HPLC trace of HPLC purified teixobactin analogue **3** (gradient: 5-95% ACN in 25 min using A: 0.1% HCOOH in water, B: ACN)

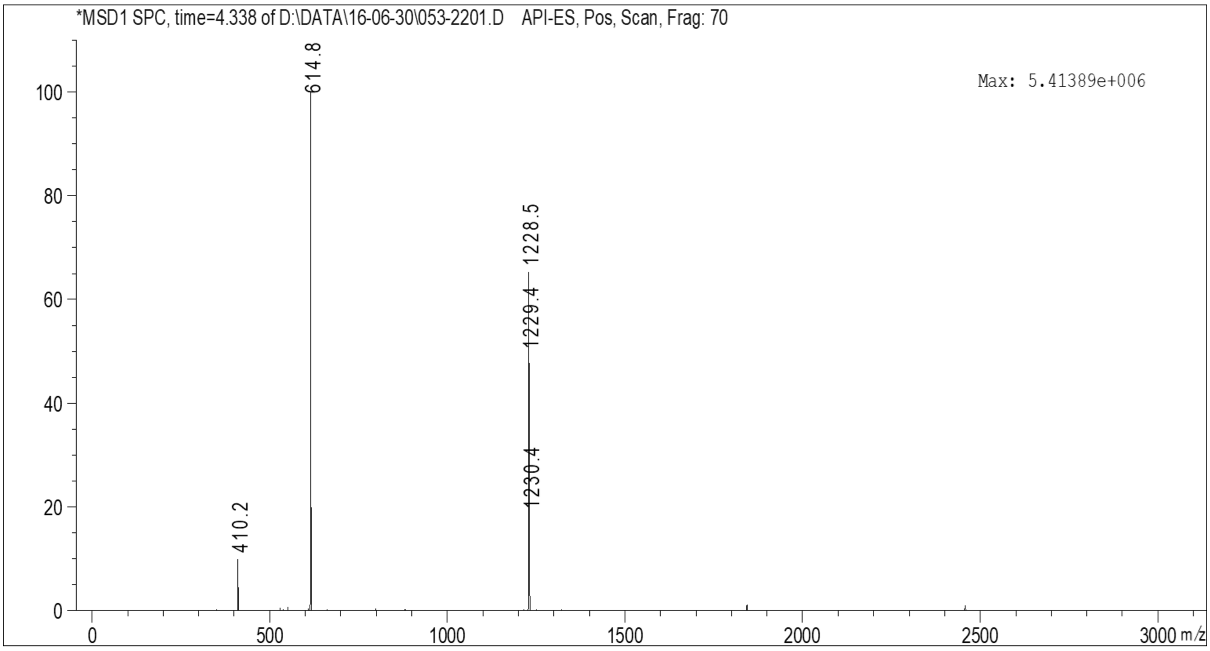

Fig. S13: ESI-MS from LC-MS of HPLC purified teixobactin analogue **3**. Exact mass calcd. for $C_{58}H_{97}N_{15}O_{14}$ = 1227.73, found M + H^+ = 1228.5, M/2 + H^+ = 614.8, M/3 + H^+ = 410.2.

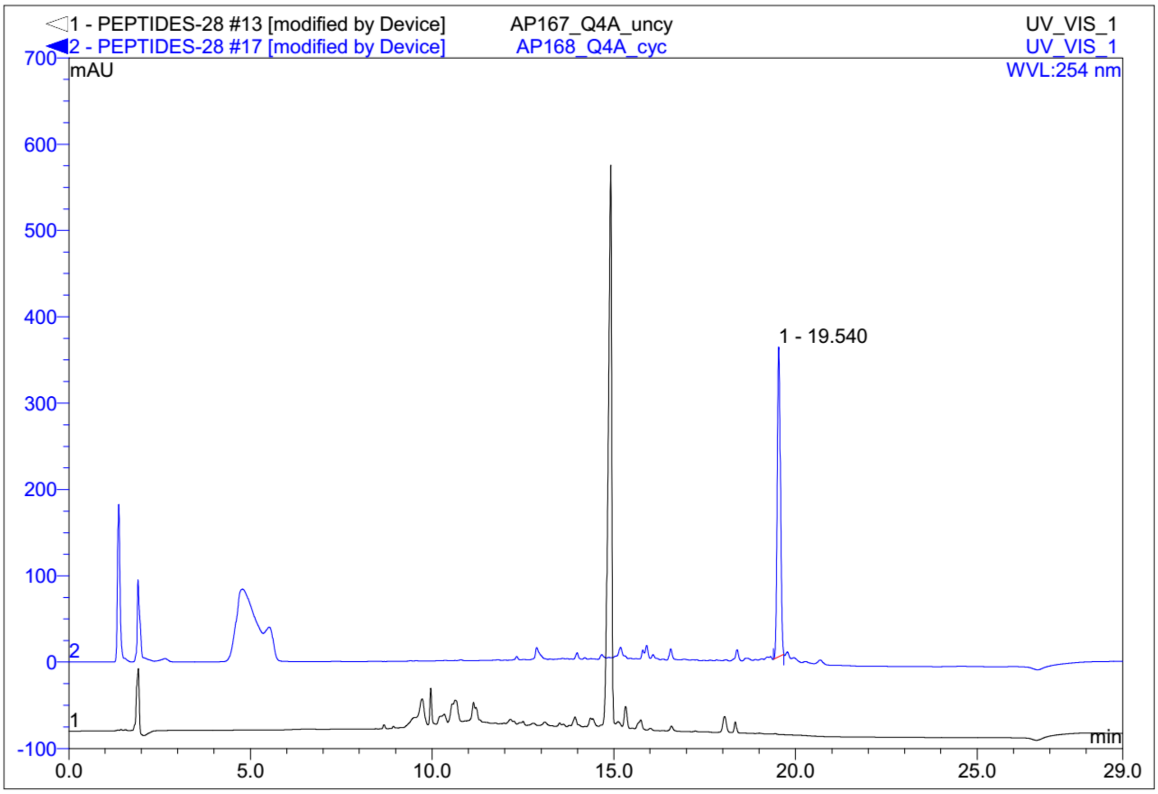

Fig. S14: HPLC trace showing the progress of cyclisation reaction for the synthesis of analogue **4** (i): conversion of the uncyclized protected teixobactin analogue t_R= 14.917 min (shown in black) to the cyclized protected teixobactin analogue t_R = 19.540 min (shown in blue) (Gradient: 5-95% in 25 min)

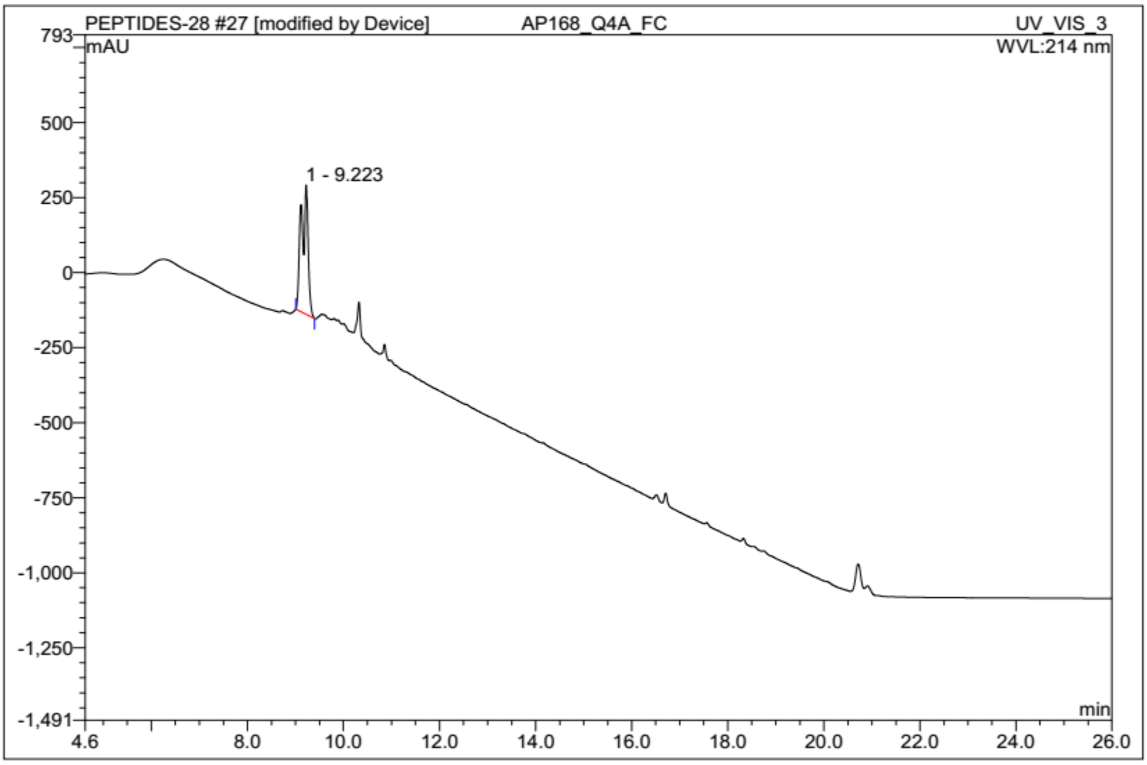

Fig. S15: HPLC trace of crude teixobactin analogue **4** t_R = 9.223 min (gradient: 5-95% ACN in 25 min using A: 0.1% HCOOH in water, B: ACN)

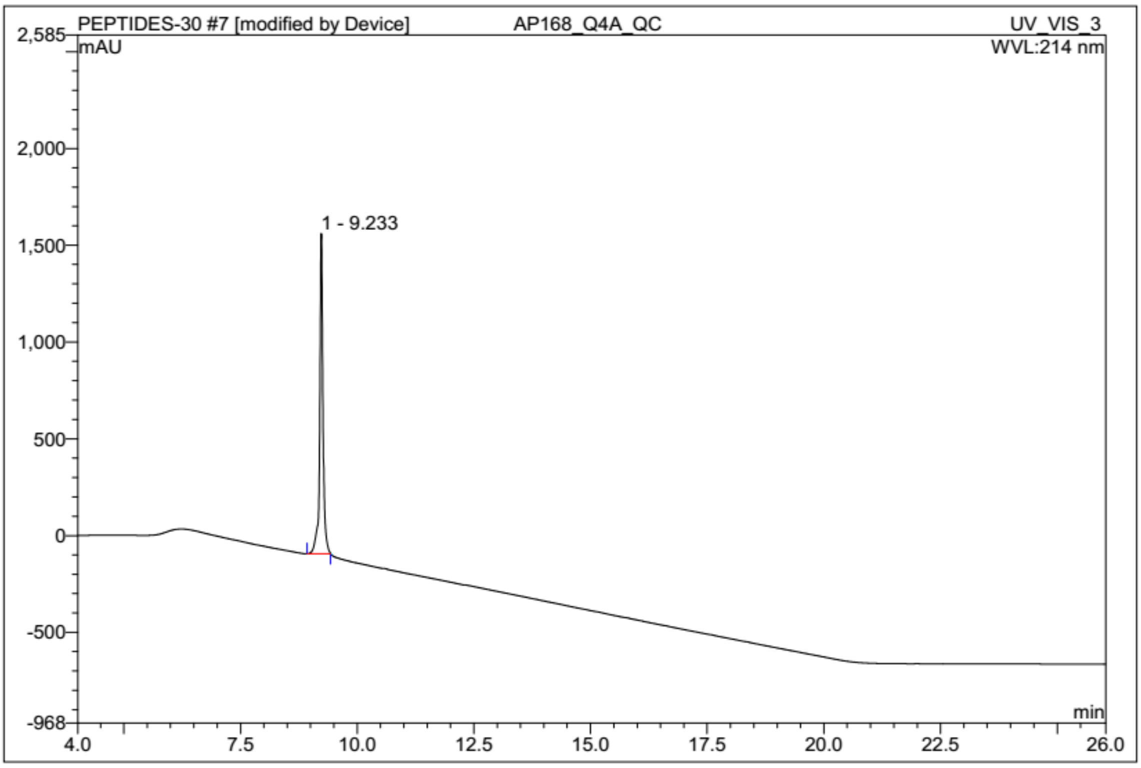

Fig. S16: HPLC trace of HPLC purified teixobactin analogue **4** (gradient: 5-95% ACN in 25 min using A: 0.1% HCOOH in water, B: ACN)

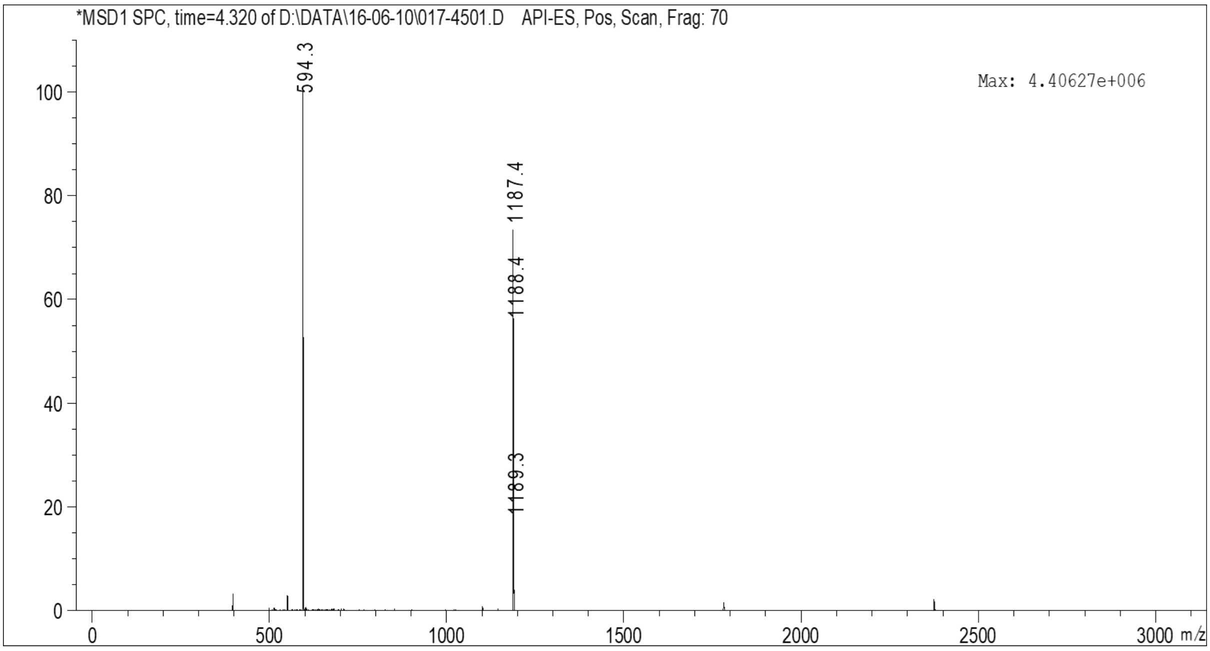

Fig. S17: ESI-MS from LC-MS of HPLC purified teixobactin analogue **4**. Exact mass calcd. for $C_{56}H_{94}N_{14}O_{14}$ = 1186.71, found M + H^+ = 1187.4, M/2 + H^+ = 594.3.

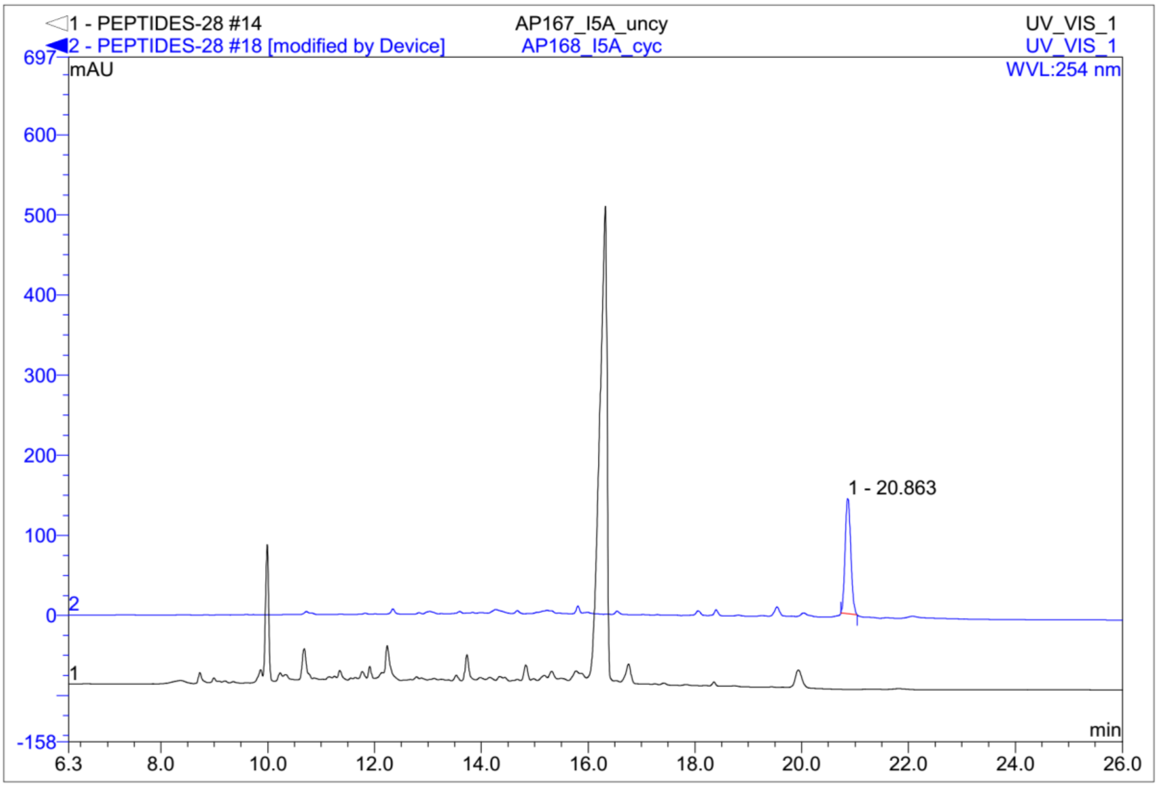

Fig. S18: HPLC trace showing the progress of cyclisation reaction for the synthesis of analogue **5** (i): conversion of the uncyclized protected teixobactin analogue t_R= 16.323 min (shown in black) to the cyclized protected teixobactin analogue t_R = 20.863 min (shown in blue) (Gradient: 5-95% in 25 min)

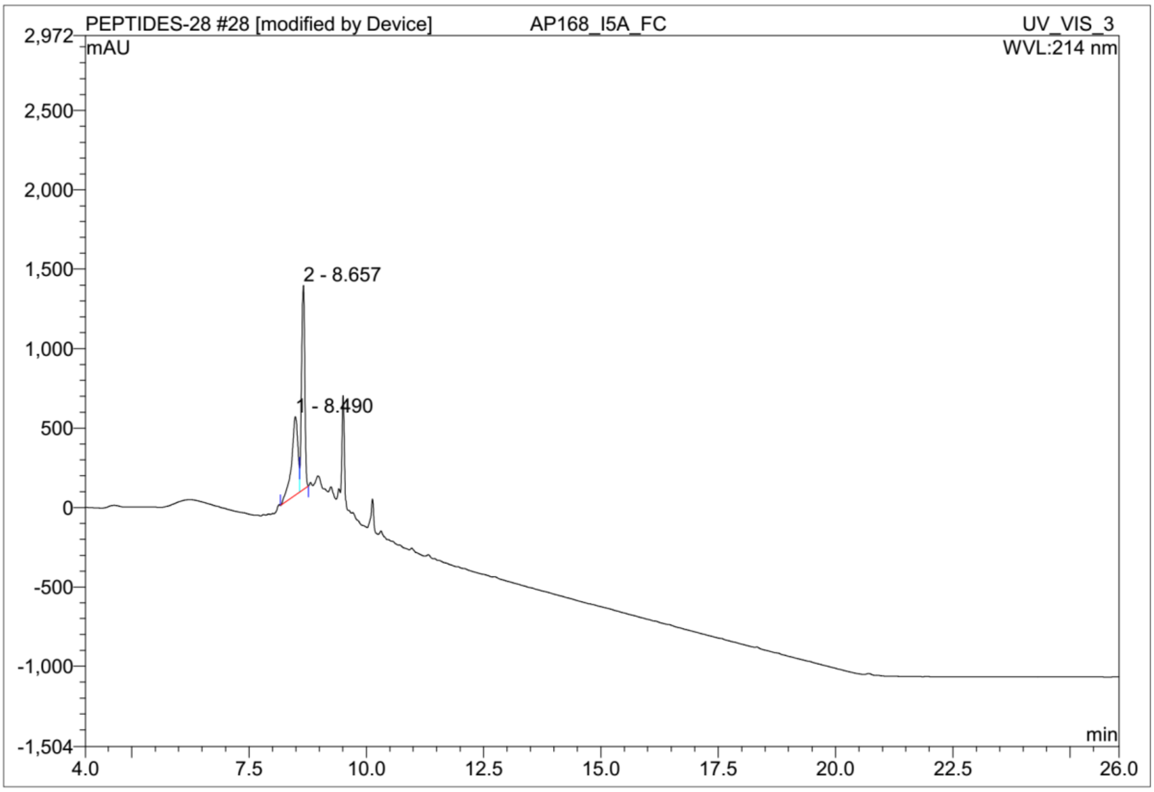

Fig. S19: HPLC trace of crude teixobactin analogue **5** t_R = 8.657 min (gradient: 5-95% ACN in 25 min using A: 0.1% HCOOH in water, B: ACN)

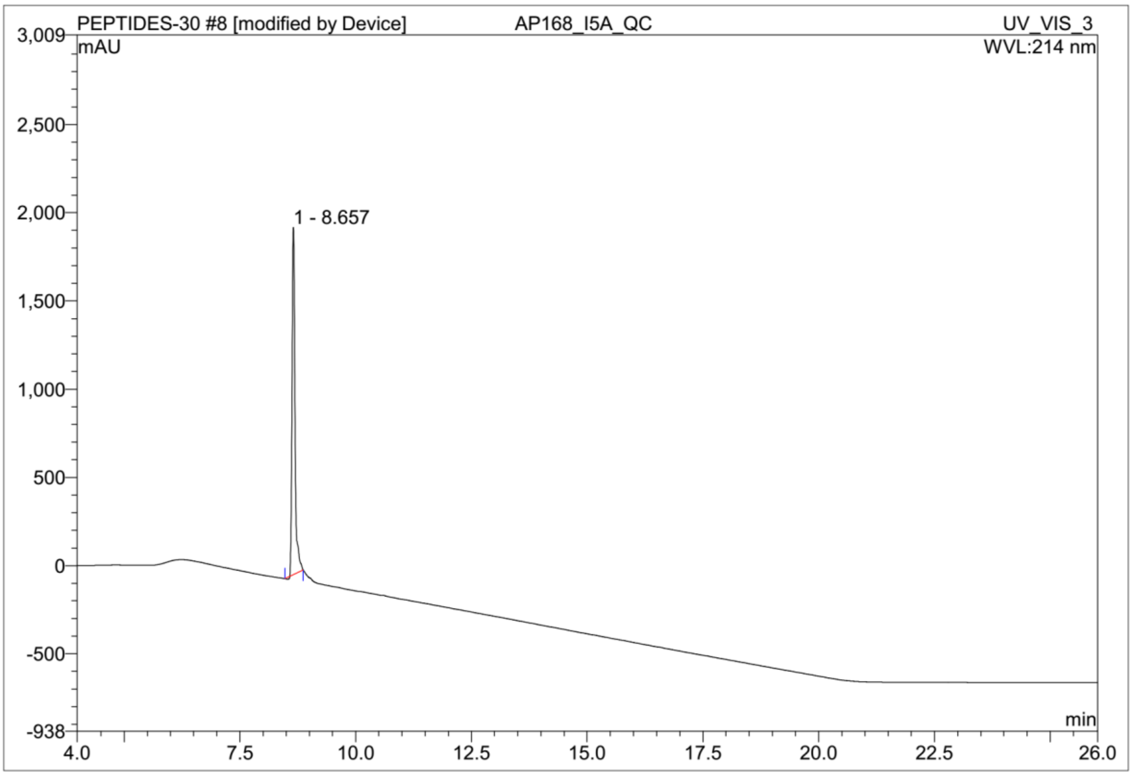

Fig. S20: HPLC trace of HPLC purified teixobactin analogue **5** (gradient: 5-95% ACN in 25 min using A: 0.1% HCOOH in water, B: ACN)

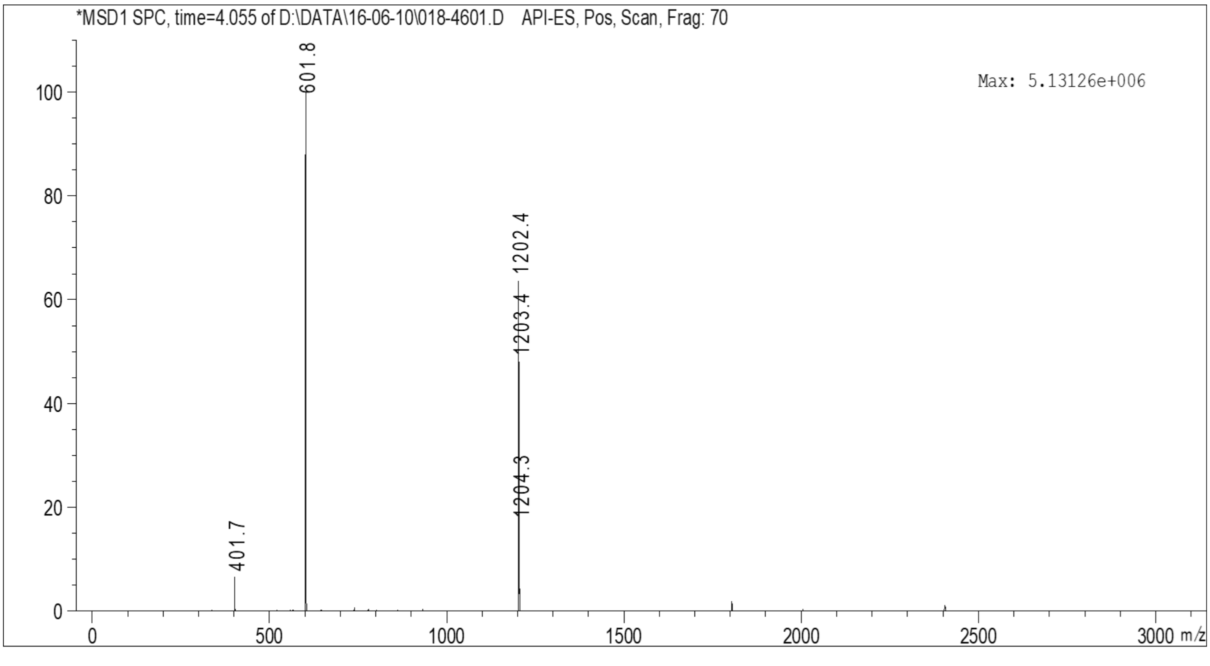

Fig. S21: ESI-MS from LC-MS of HPLC purified teixobactin analogue **5**. Exact mass calcd. for $C_{55}H_{91}N_{15}O_{15}$ = 1201.68, found M + H^+ = 1202.4, M/2 + H^+ = 601.8, M/3 + H^+ = 401.7.

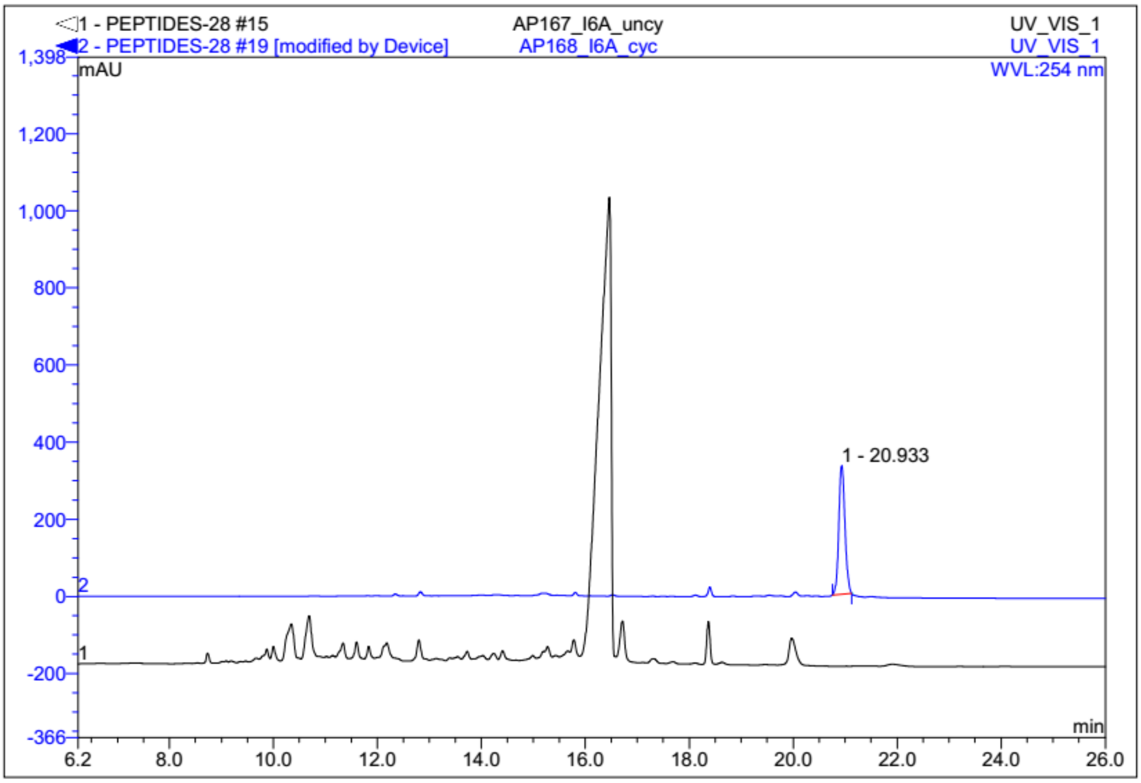

Fig. S22: HPLC trace showing the progress of cyclisation reaction for the synthesis of analogue **6** (i): conversion of the uncyclized protected teixobactin analogue t_R= 16.467 min (shown in black) to the cyclized protected teixobactin analogue t_R = 20.933 min (shown in blue) (Gradient: 5-95% in 25 min)

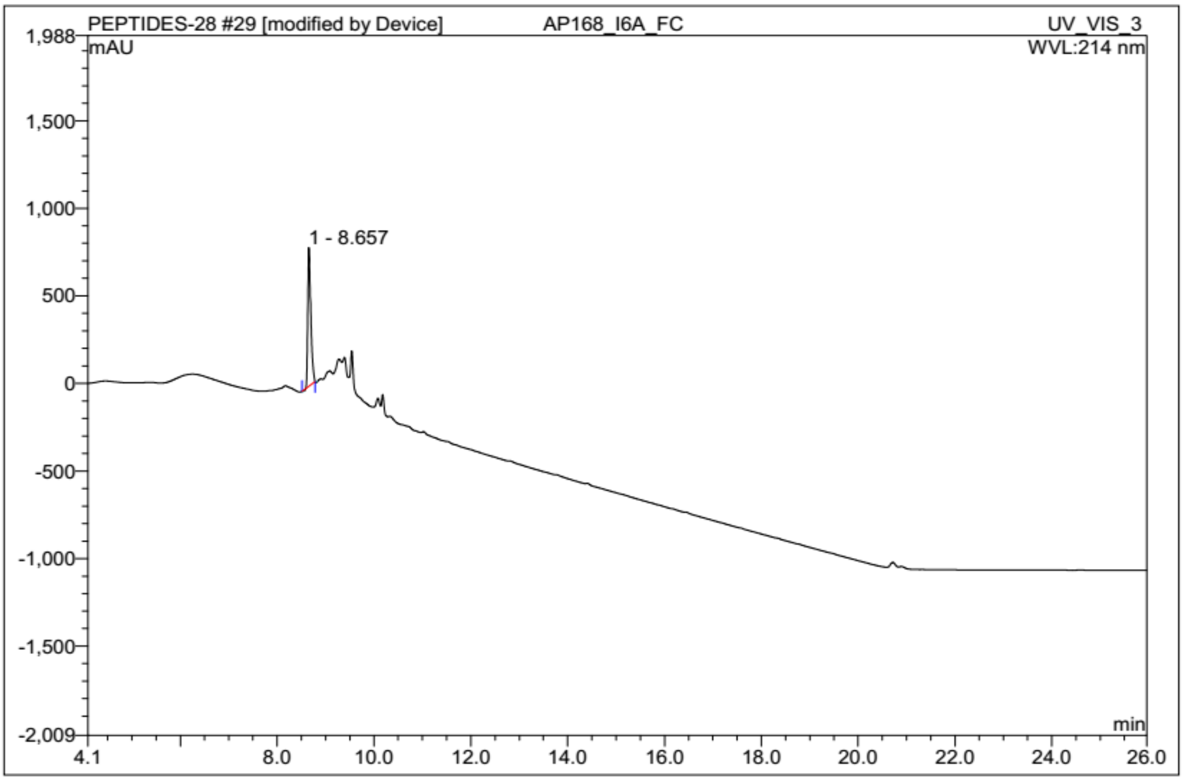

Fig. S23: HPLC trace of crude teixobactin analogue **6** t_R = 8.657 min (gradient: 5-95% ACN in 25 min using A: 0.1% HCOOH in water, B: ACN)

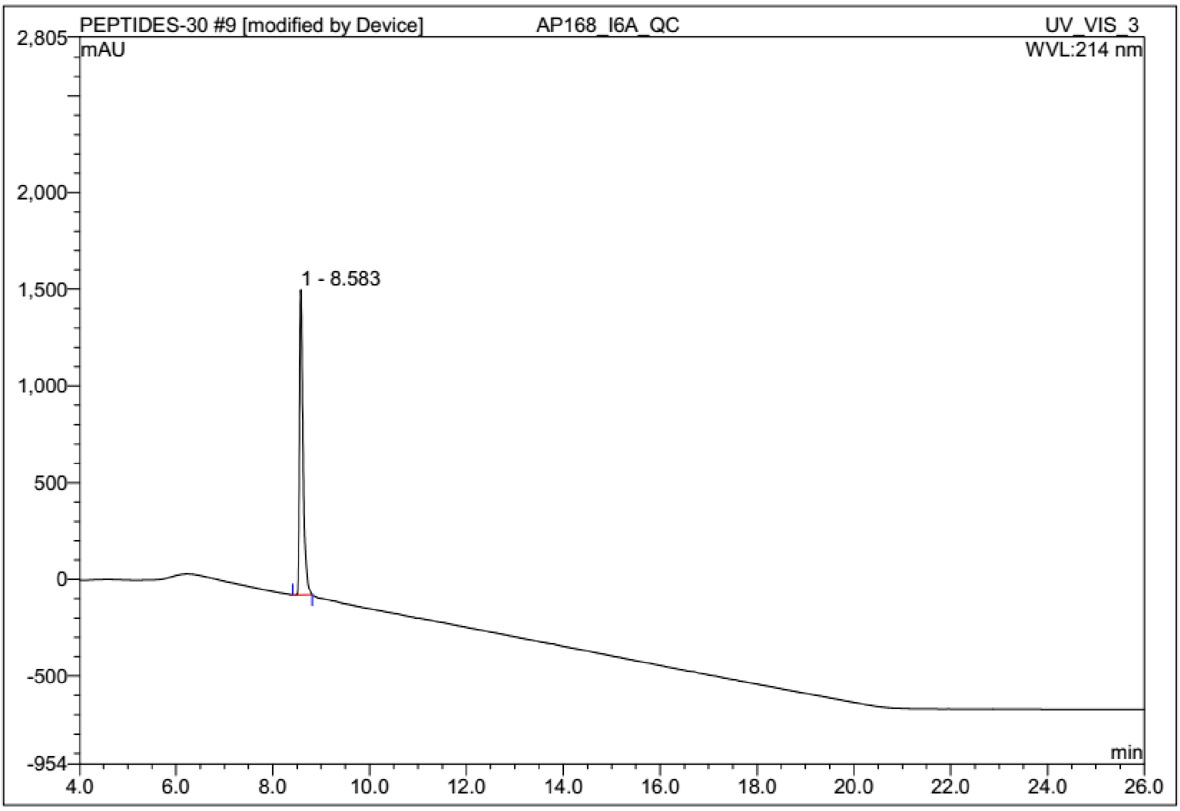

Fig. S24: HPLC trace of HPLC purified teixobactin analogue **6** (gradient: 5-95% ACN in 25 min using A: 0.1% HCOOH in water, B: ACN)

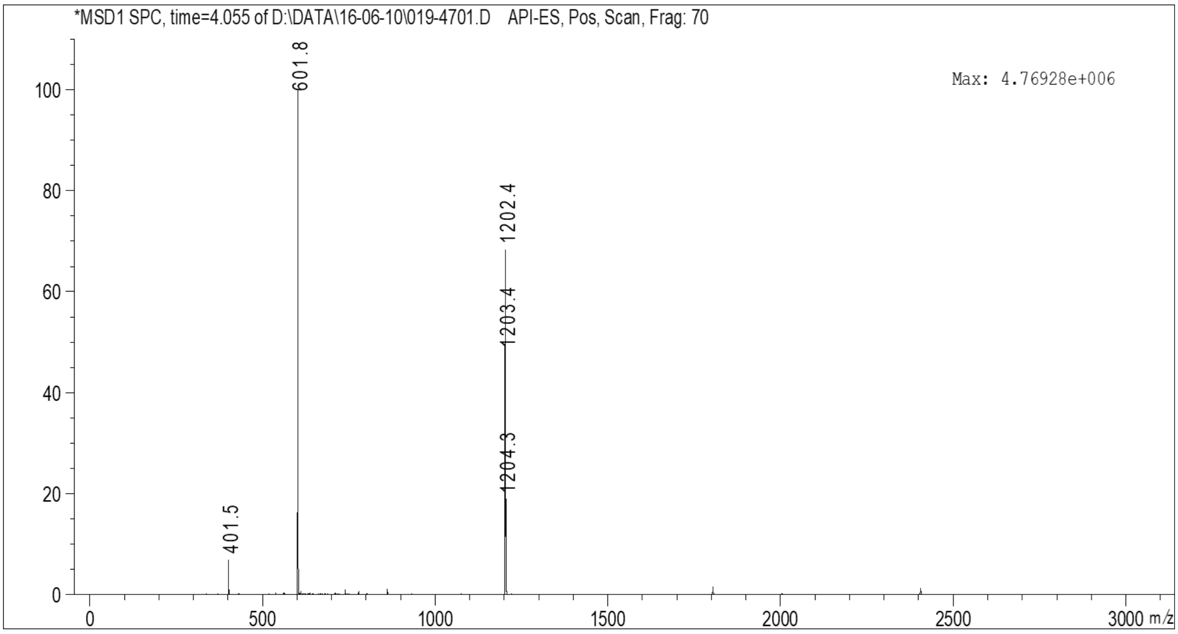

Fig. S25: ESI-MS from LC-MS of HPLC purified teixobactin analogue **6**. Exact mass calcd. for $C_{55}H_{91}N_{15}O_{15}$ = 1201.68, found M + H^+ = 1202.4, M/2 + H^+ = 601.8, M/3 + H^+ = 401.7.

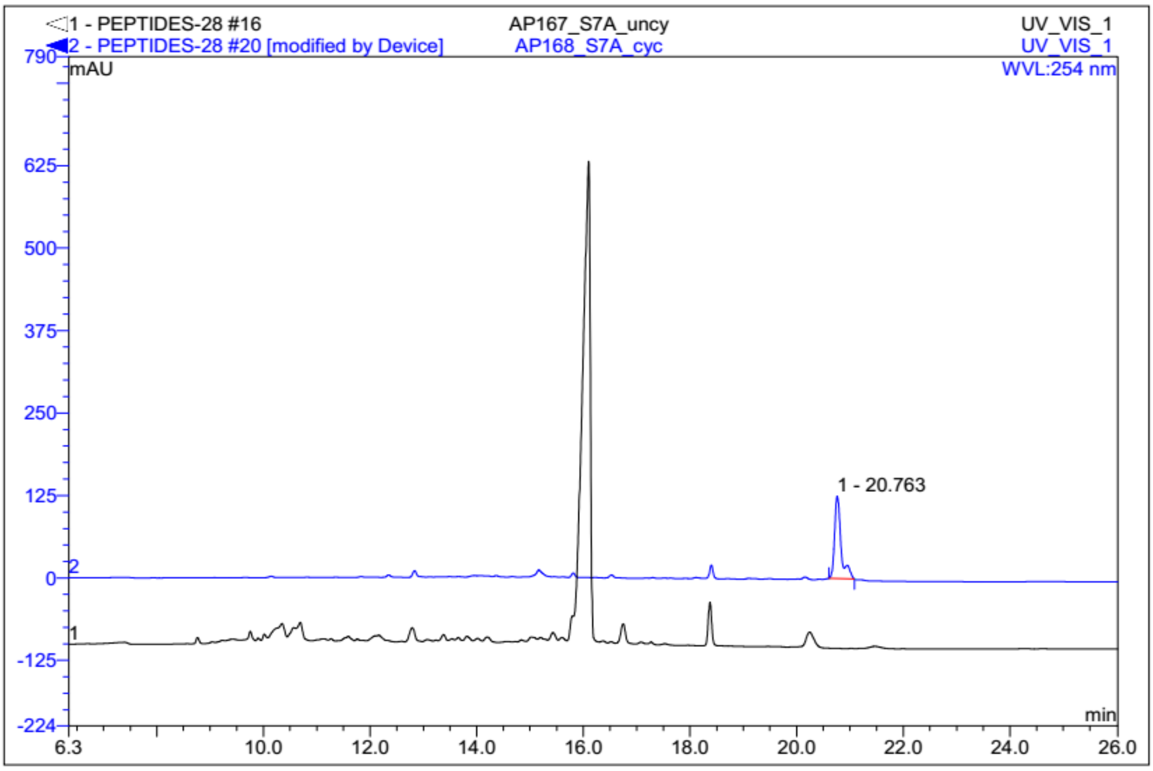

Fig. S26: HPLC trace showing the progress of cyclisation reaction for the synthesis of analogue **7** (i): conversion of the uncyclized protected teixobactin analogue t_R= 16.100 min (shown in black) to the cyclized protected teixobactin analogue t_R = 20.763 min (shown in blue) (Gradient: 5-95% in 25 min)

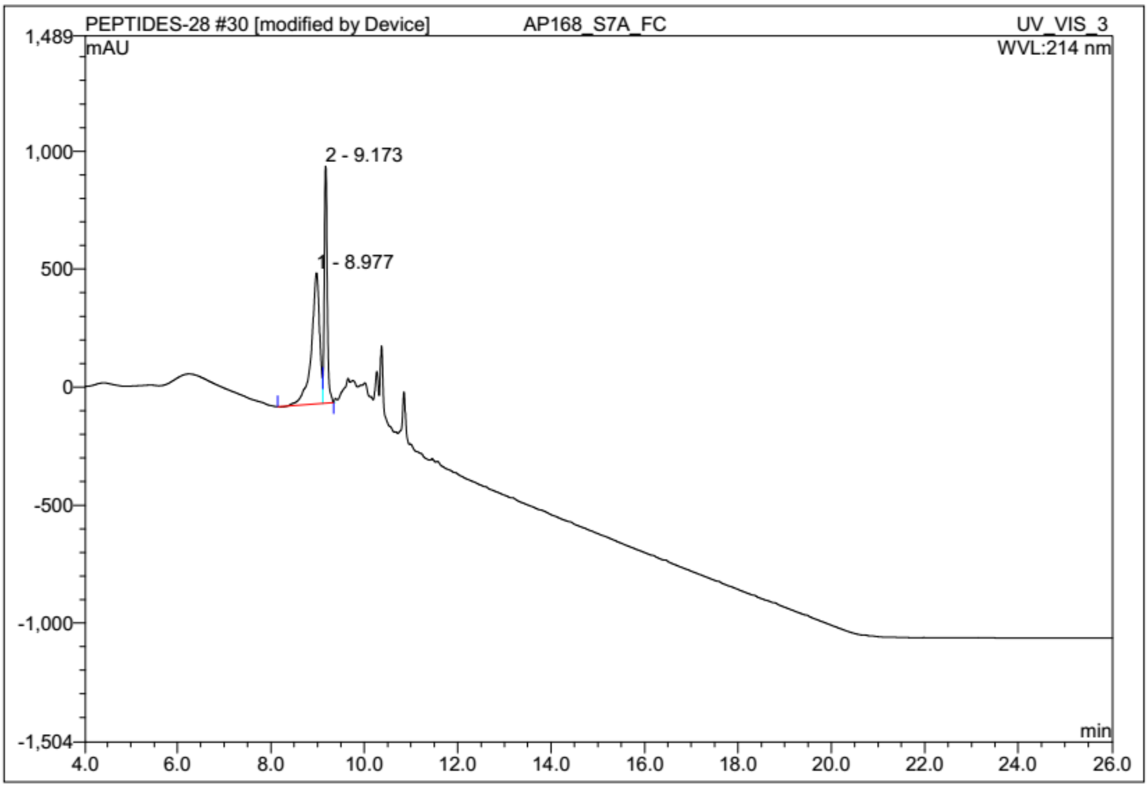

Fig. S27: HPLC trace of crude teixobactin analogue **7** t_R = 9.173 min (gradient: 5-95% ACN in 25 min using A: 0.1% HCOOH in water, B: ACN)

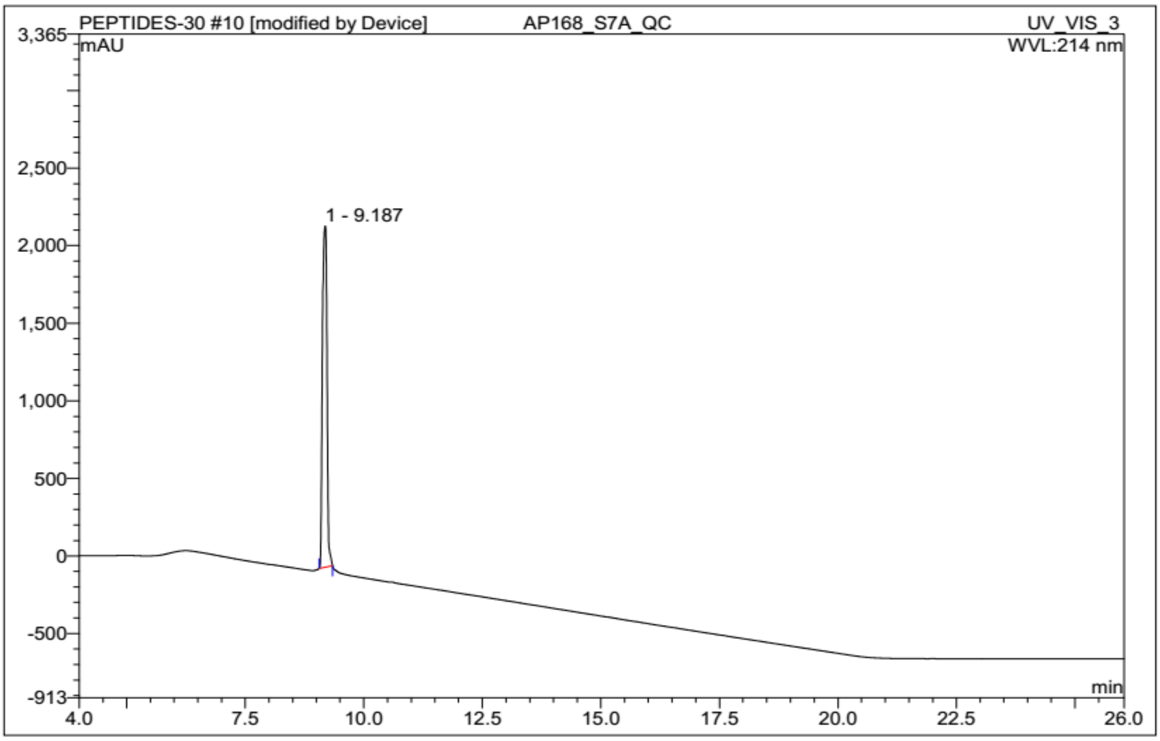

Fig. S28: HPLC trace of HPLC purified teixobactin analogue **7** (gradient: 5-95% ACN in 25 min using A: 0.1% HCOOH in water, B: ACN)

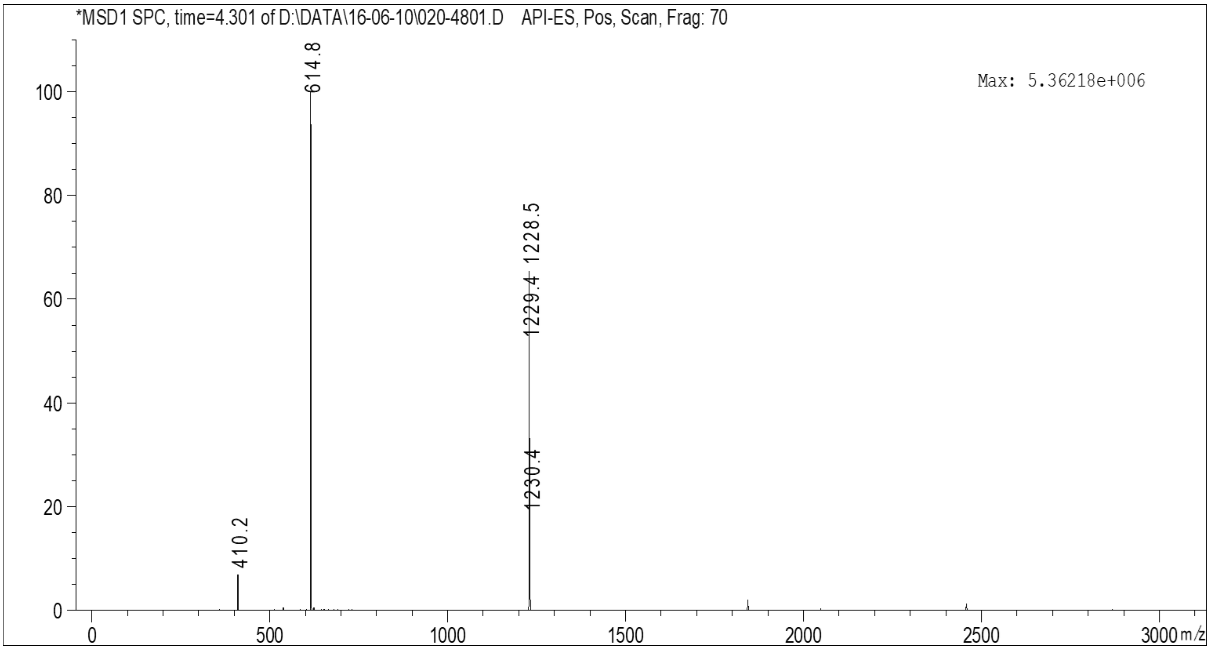

Fig. S29: ESI-MS from LC-MS of HPLC purified teixobactin analogue **7**. Exact mass calcd. for $C_{58}H_{97}N_{15}O_{14}$ = 1227.73, found M + H^+ = 1228.5, M/2 + H^+ = 614.8, M/3 + H^+ = 410.2.

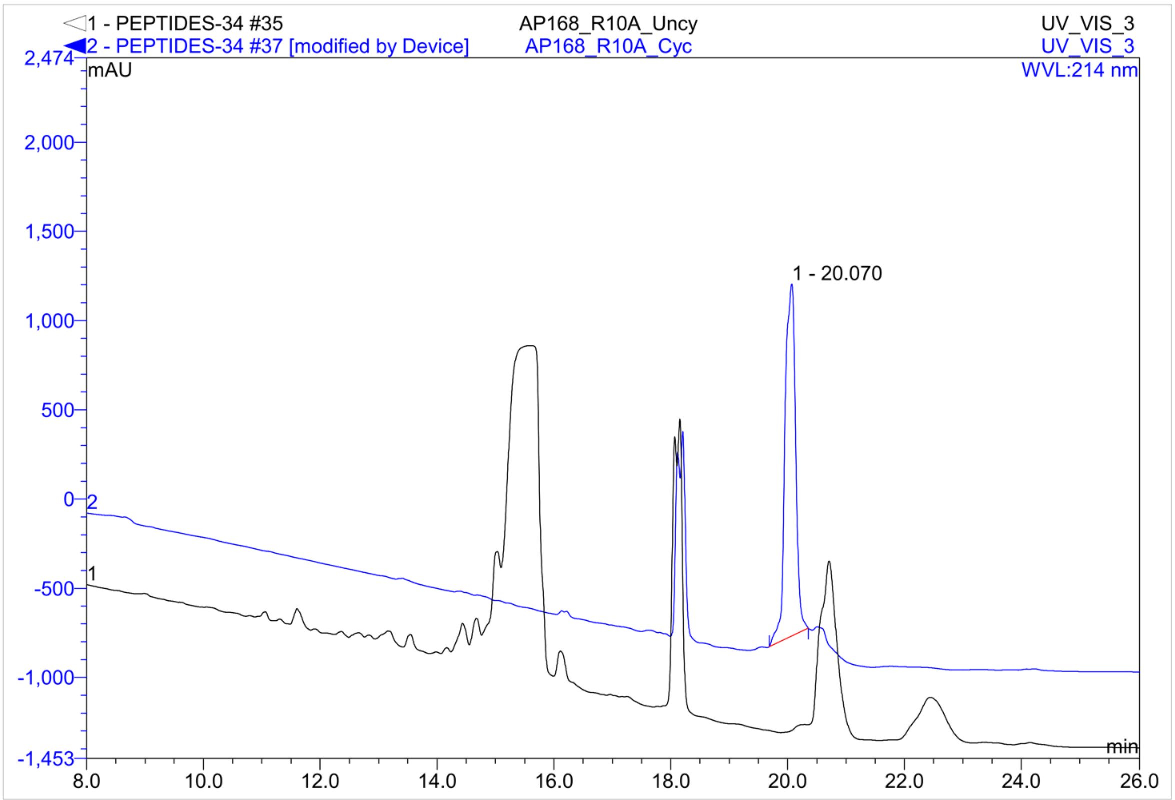

Fig. S30: HPLC trace showing the progress of cyclisation reaction for the synthesis of analogue **8** (i): conversion of the uncyclized protected teixobactin analogue t_R= 15.640 min (shown in black) to the cyclized protected teixobactin analogue t_R = 20.763 min (shown in blue) (Gradient: 5-95% in 25 min)

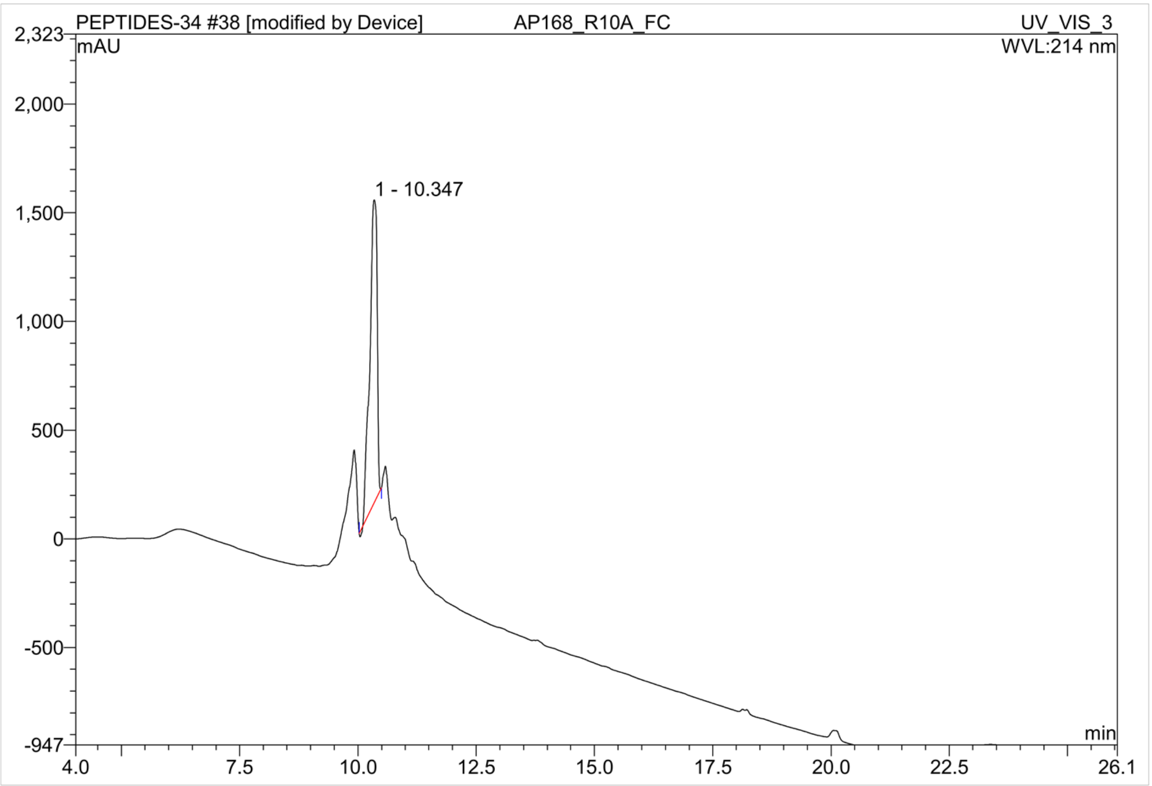

Fig. S31: HPLC trace of crude teixobactin analogue **8** t_R = 10.347 min (gradient: 5-95% ACN in 25 min using A: 0.1% HCOOH in water, B: ACN)

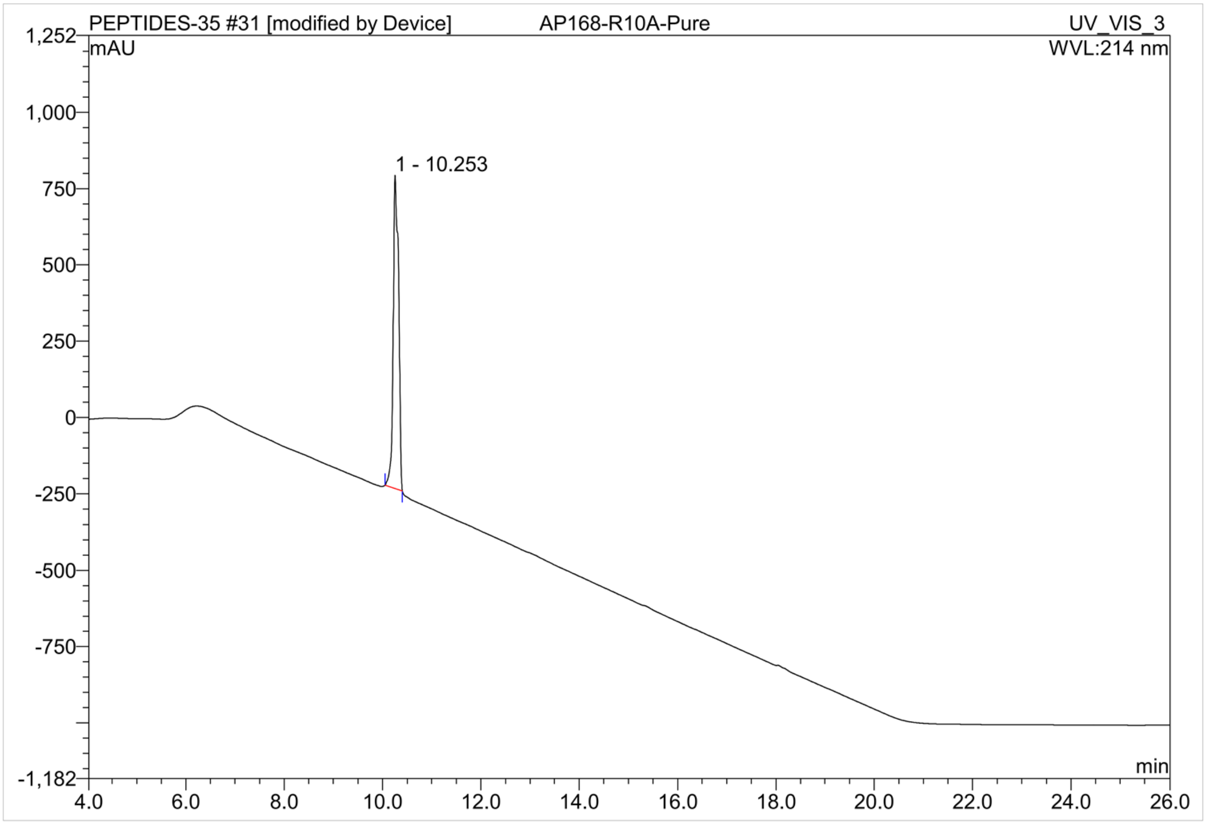

Fig. S32: HPLC trace of HPLC purified teixobactin analogue **8** (gradient: 5-95% ACN in 25 min using A: 0.1% HCOOH in water, B: ACN)

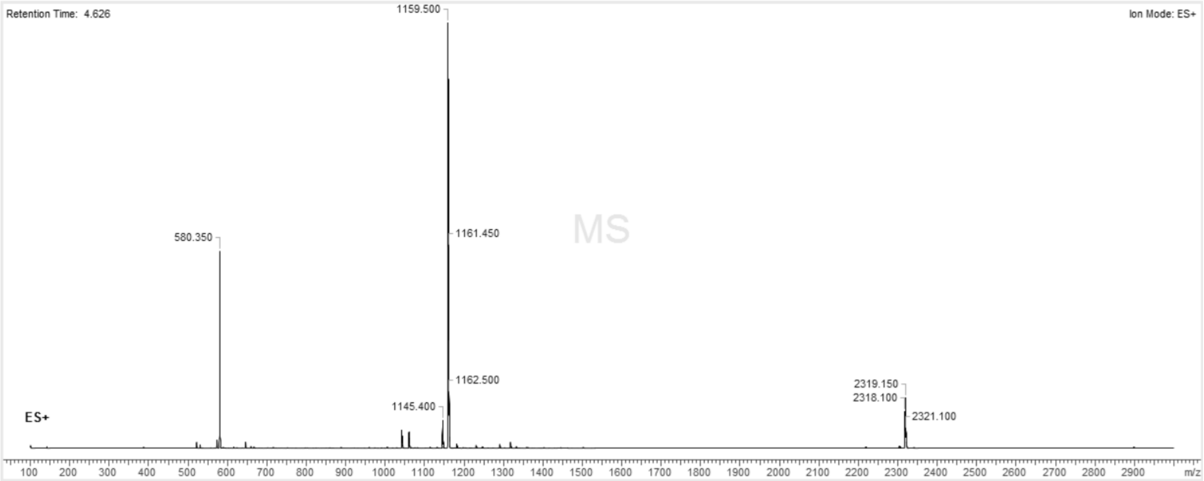

Fig. S33: ESI-MS from LC-MS of HPLC purified teixobactin analogue **8**. Exact mass calcd. for $C_{55}H_{90}N_{12}O_{15}$ = 1158.66, found M + H^+ = 1159.50, M/2 + H^+ = 580.35.

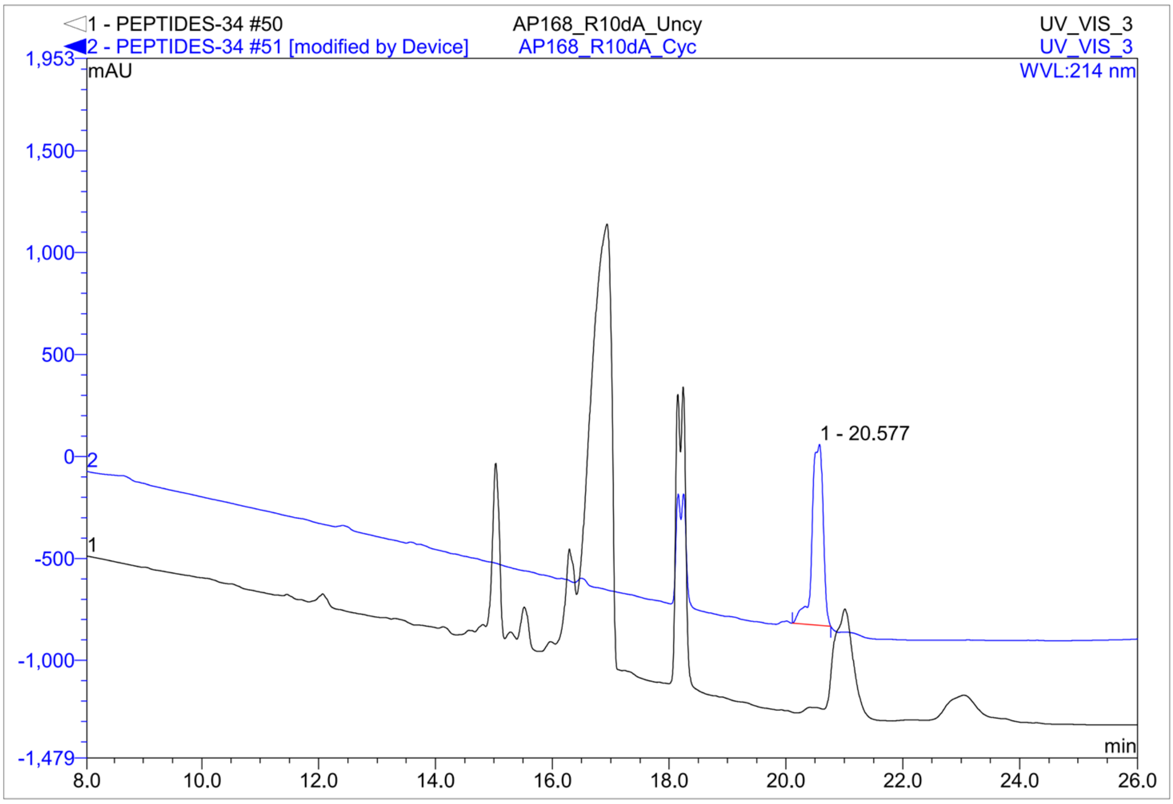

Fig. S34: HPLC trace showing the progress of cyclisation reaction for the synthesis of analogue **9** (i): conversion of the uncyclized protected teixobactin analogue t_R= 16.940 min (shown in black) to the cyclized protected teixobactin analogue t_R = 20.577 min (shown in blue) (Gradient: 5-95% in 25 min)

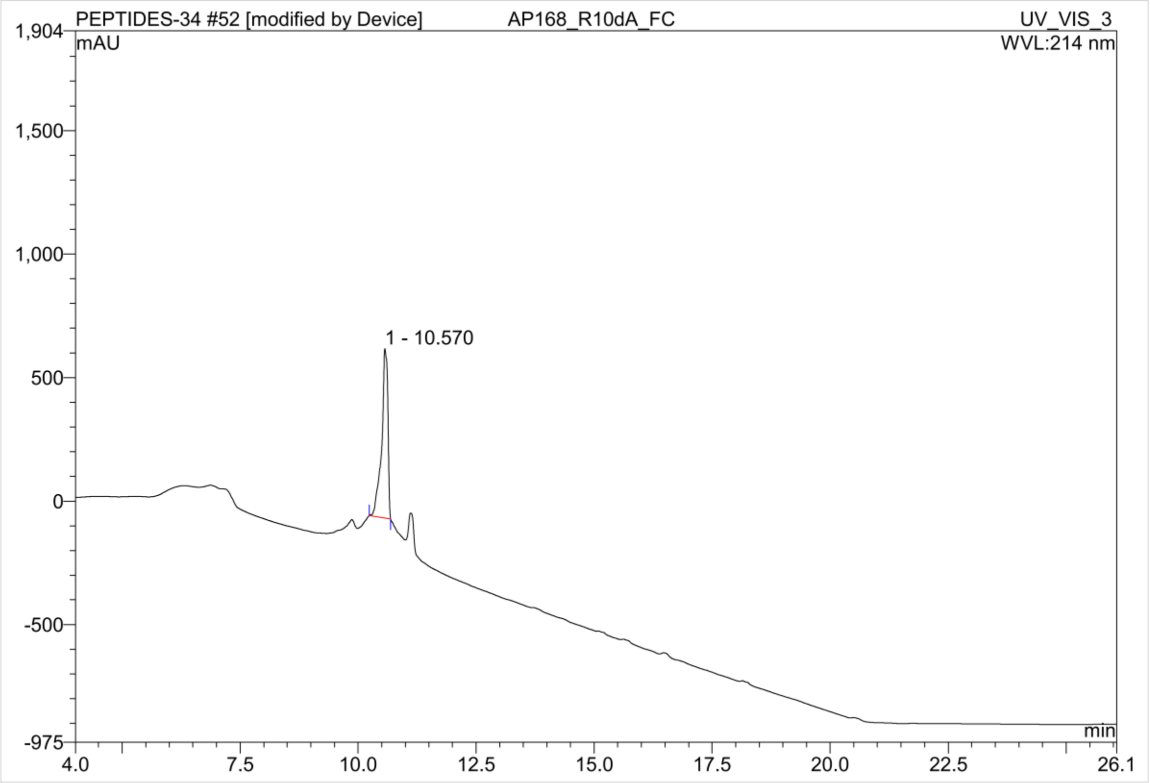

Fig. S35: HPLC trace of crude teixobactin analogue **9** t_R = 10.570 min (gradient: 5-95% ACN in 25 min using A: 0.1% HCOOH in water, B: ACN)

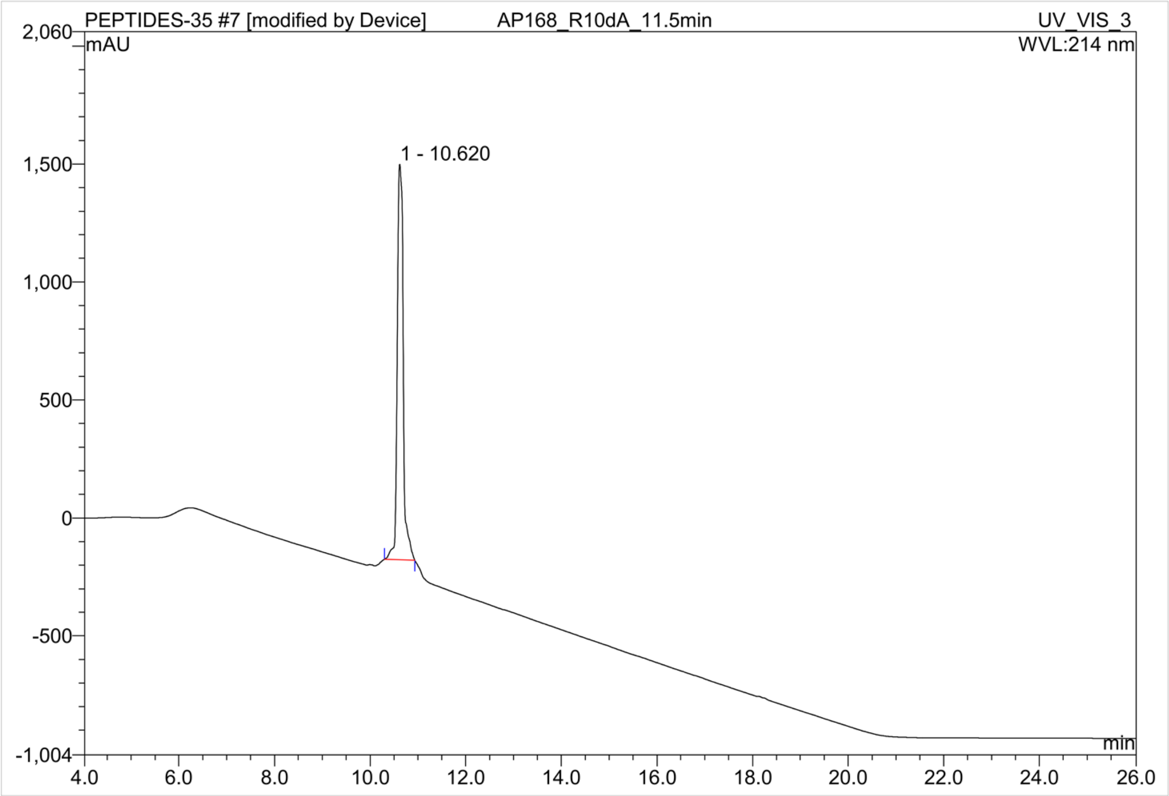

Fig. S36: HPLC trace of HPLC purified teixobactin analogue **9** (gradient: 5-95% ACN in 25 min using A: 0.1% HCOOH in water, B: ACN)

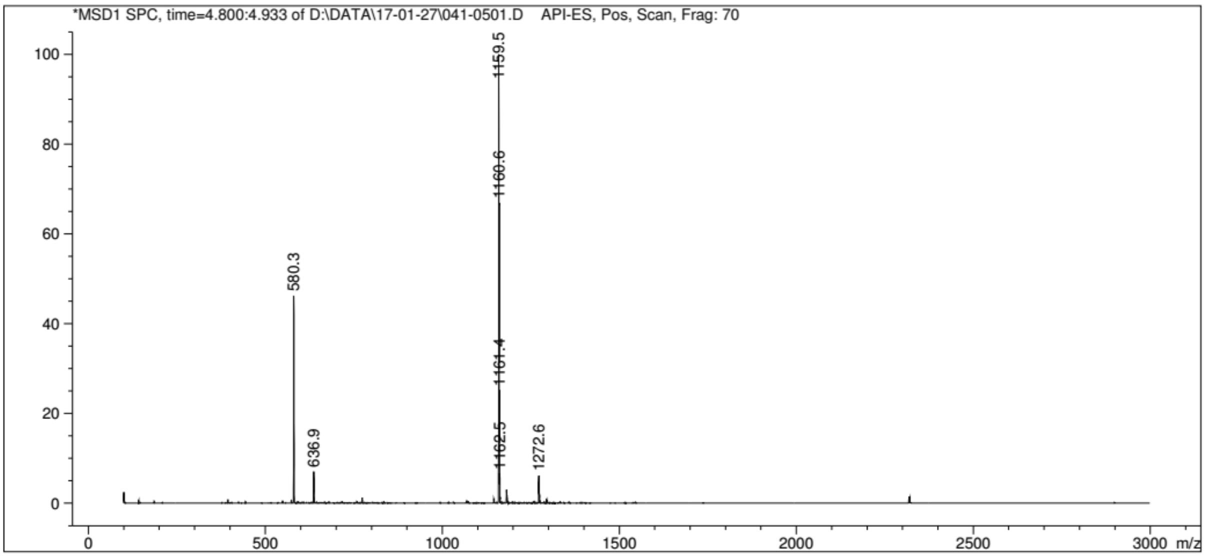

Fig. S37: ESI-MS from LC-MS of HPLC purified teixobactin analogue **9**. Exact mass calcd. for $C_{55}H_{90}N_{12}O_{15}$ = 1158.66, found M + H^+ = 1159.5, M/2 + H^+ = 580.3.

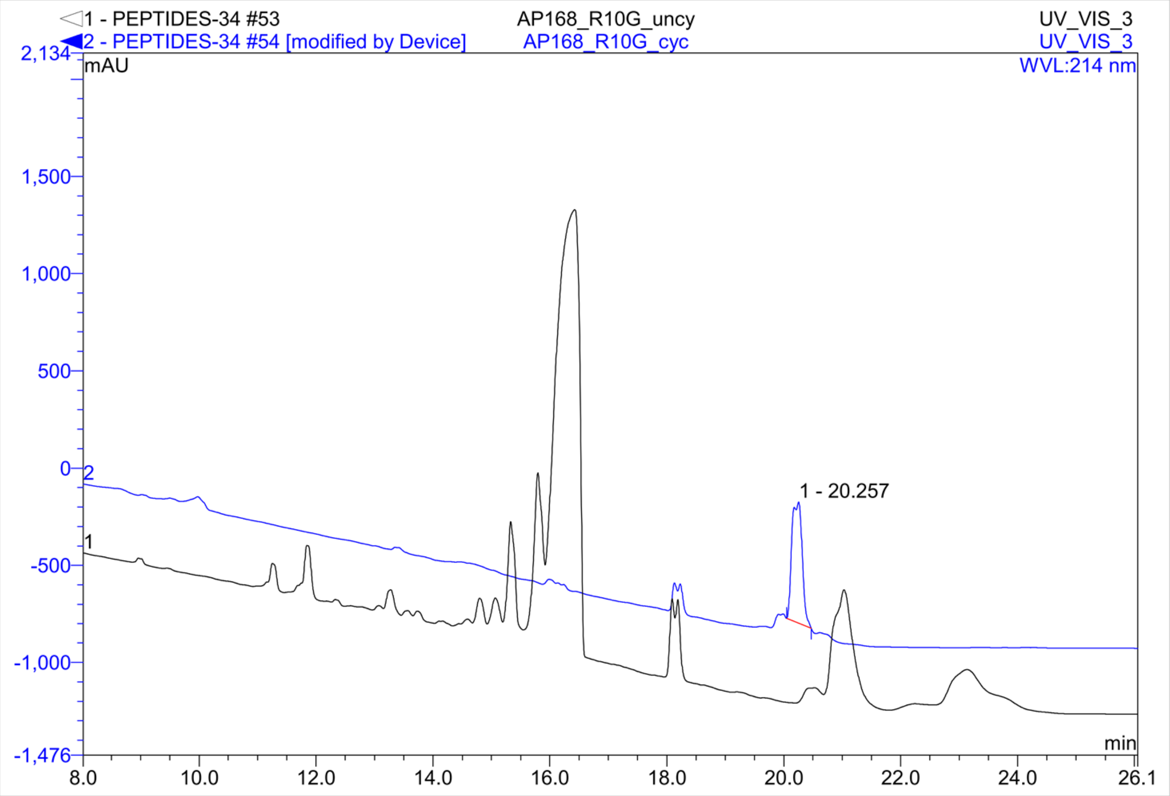

Fig. S38: HPLC trace showing the progress of cyclisation reaction for the synthesis of analogue **10** (i): conversion of the uncyclized protected teixobactin analogue t_R= 16.423 min (shown in black) to the cyclized protected teixobactin analogue t_R = 20.257 min (shown in blue) (Gradient: 5-95% in 25 min)

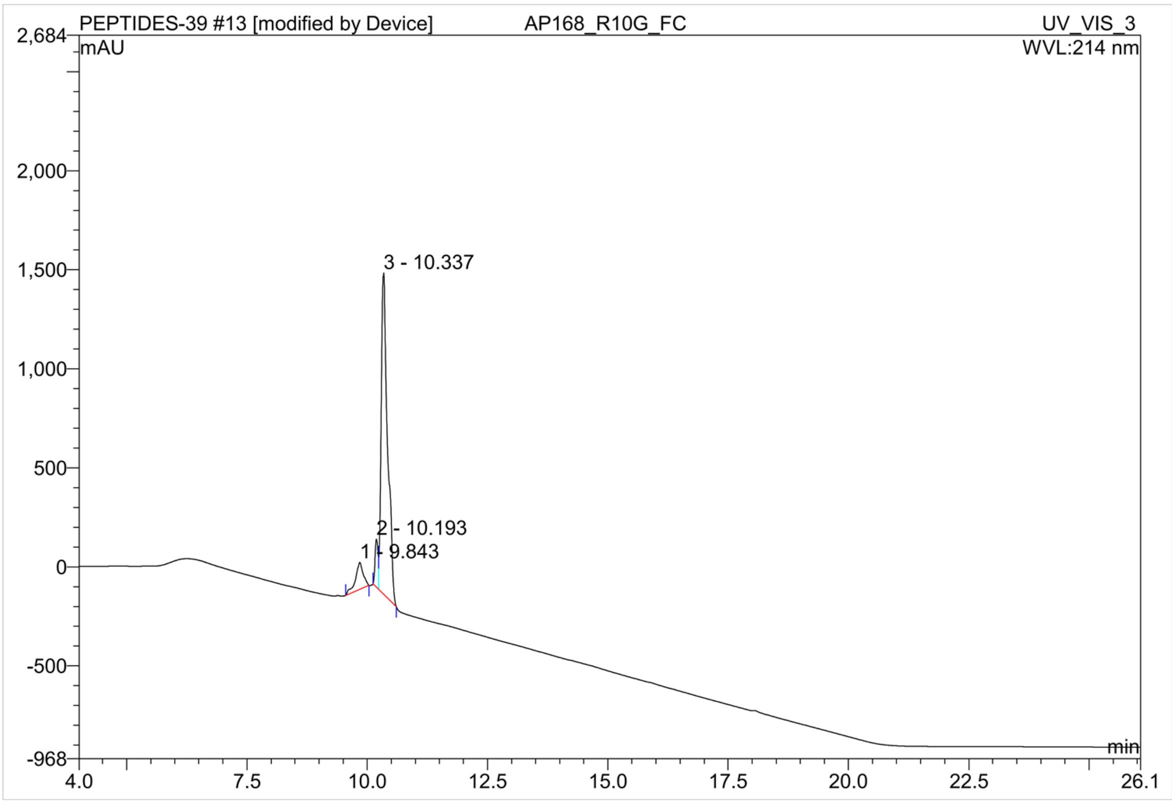

Fig. S39: HPLC trace of crude teixobactin analogue **10** t_R = 10.373 min (gradient: 5-95% ACN in 25 min using A: 0.1% HCOOH in water, B: ACN)

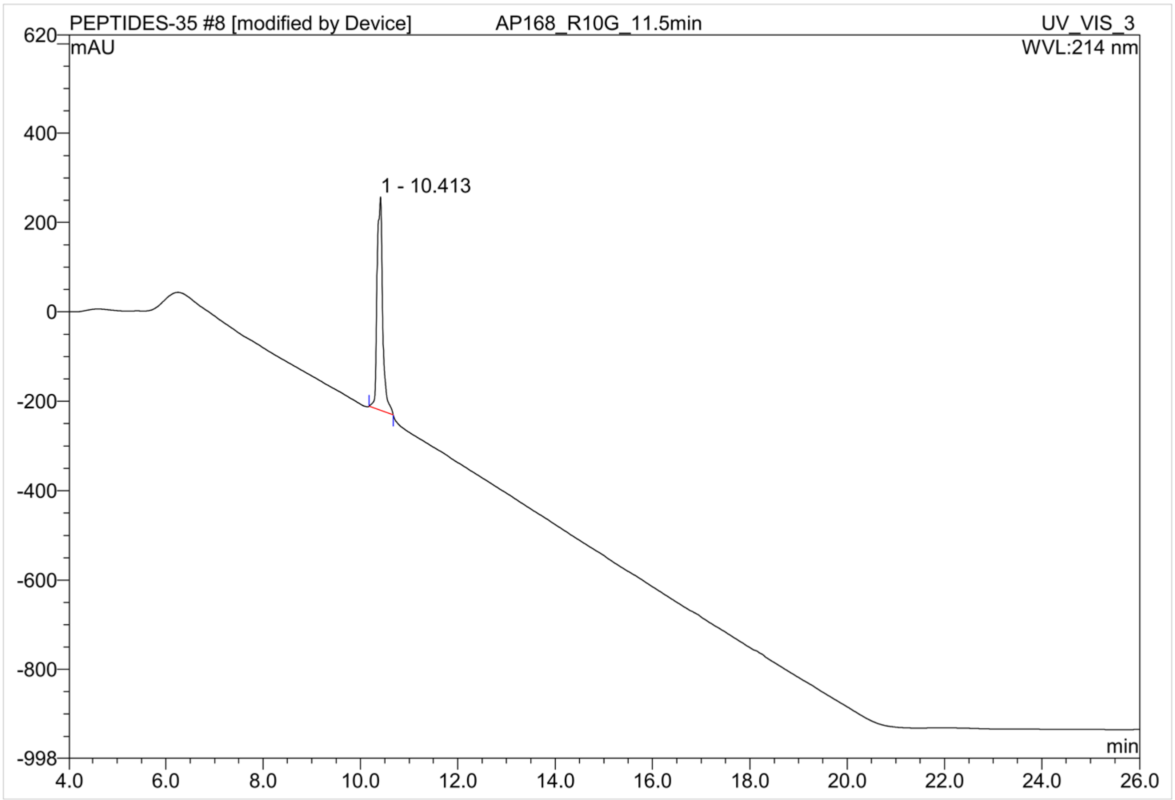

Fig. S40: HPLC trace of HPLC purified teixobactin analogue **10** (gradient: 5-95% ACN in 25 min using A: 0.1% HCOOH in water, B: ACN)

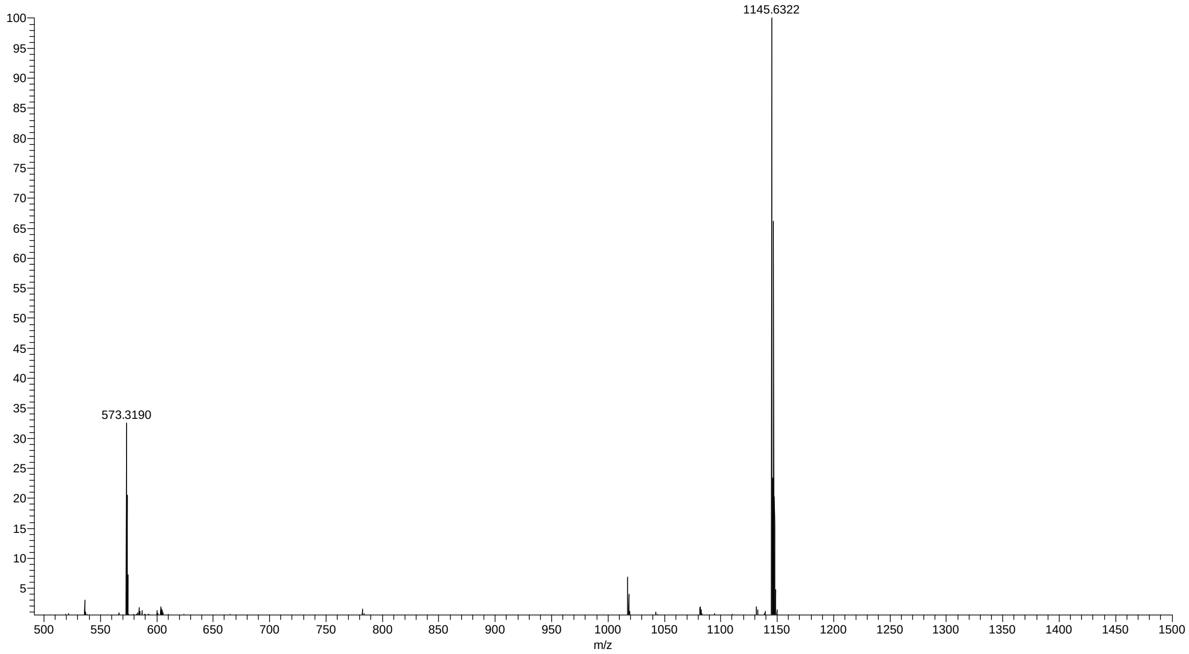

Fig. S41: HRMS of HPLC purified teixobactin analogue **10**. Exact mass calcd. for $C_{54}H_{88}N_{12}O_{15}$ = 1144.6492, found M + H^+ = 1145.6322, M/2 + H^+ = 573.3190.

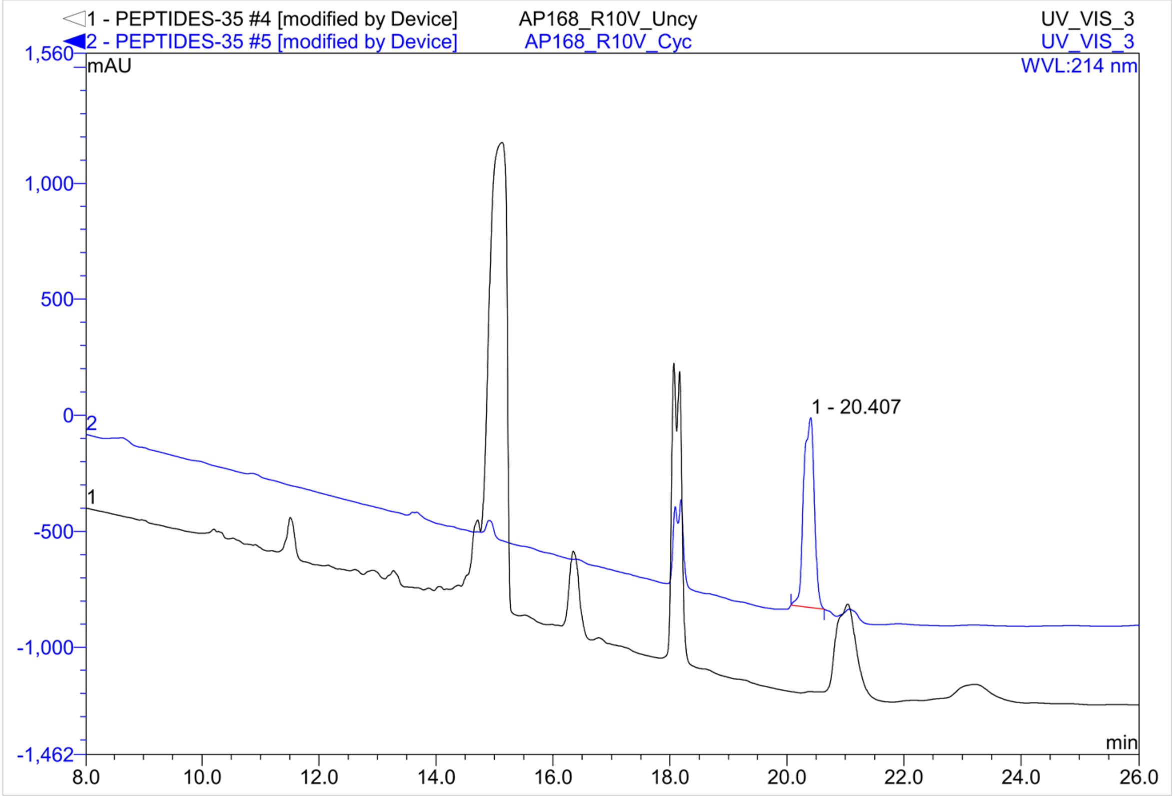

Fig. S42: HPLC trace showing the progress of cyclisation reaction for the synthesis of analogue **11** (i): conversion of the uncyclized protected teixobactin analogue t_R= 15.130 min (shown in black) to the cyclized protected teixobactin analogue t_R = 20.407 min (shown in blue) (Gradient: 5-95% in 25 min)

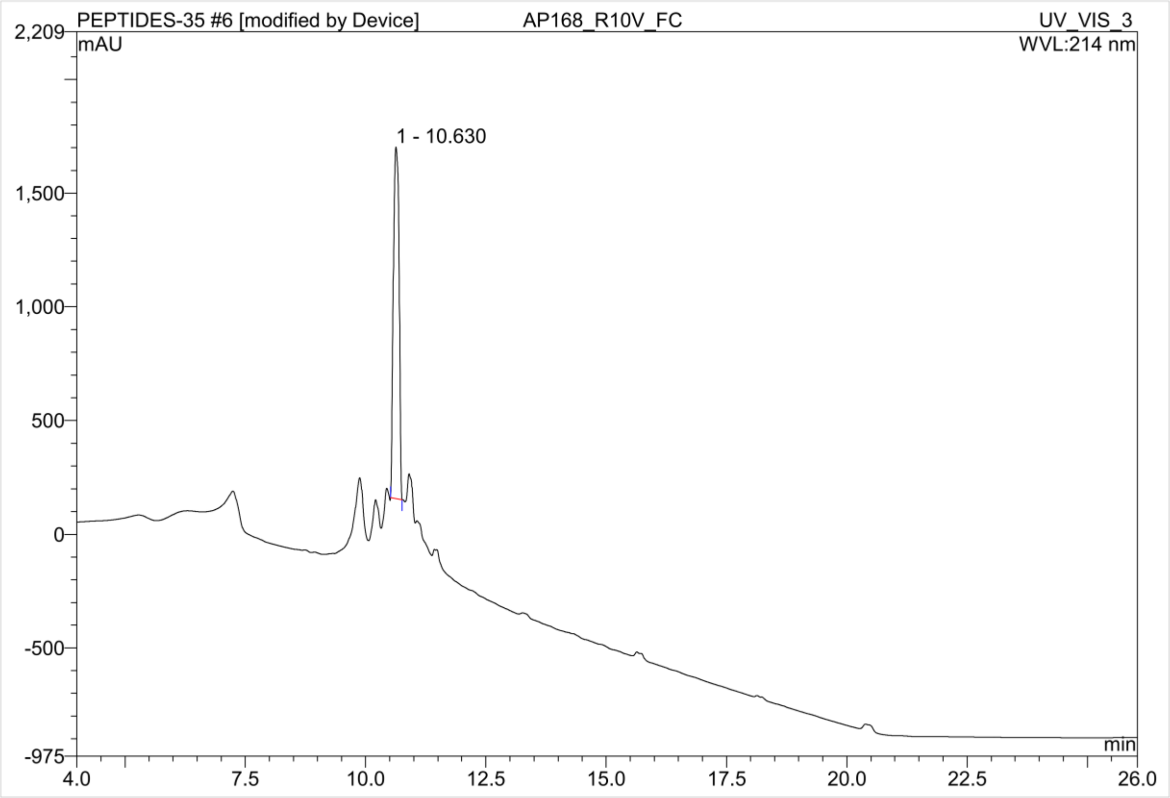

Fig. S43: HPLC trace of crude teixobactin analogue **11** t_R = 10.630 min (gradient: 5-95% ACN in 25 min using A: 0.1% HCOOH in water, B: ACN)

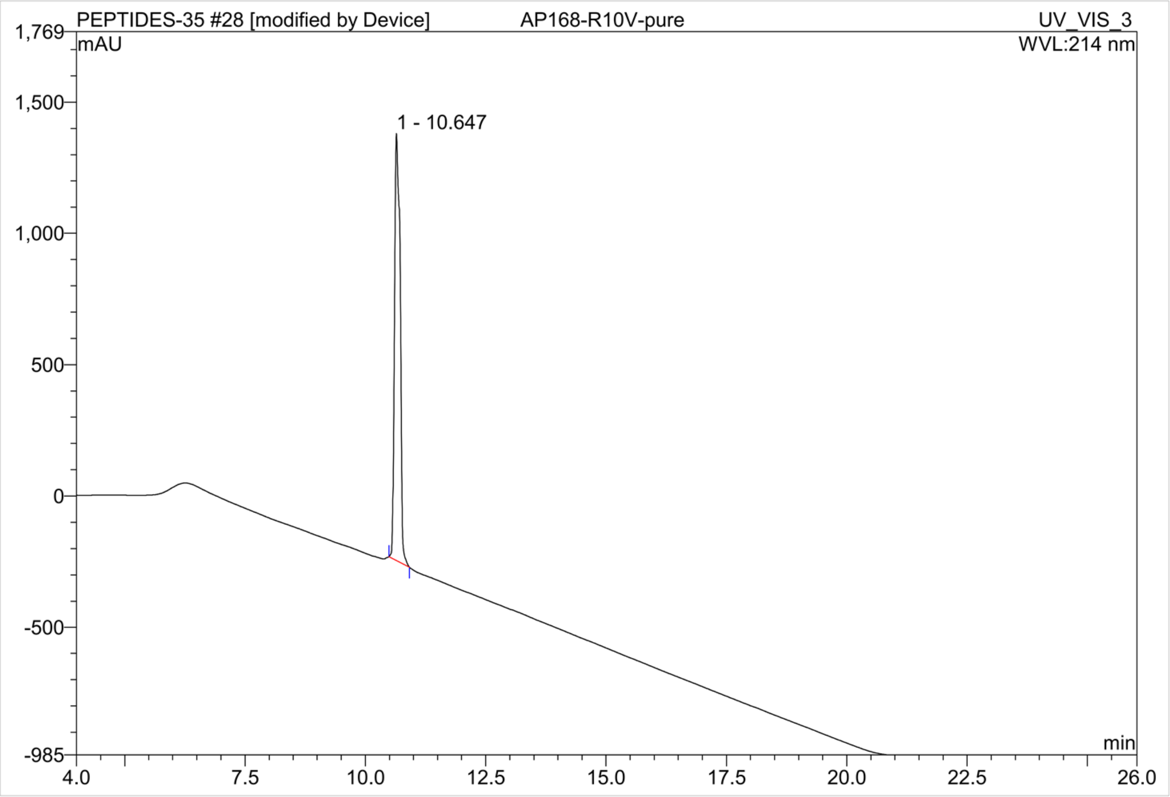

Fig. S44: HPLC trace of HPLC purified teixobactin analogue **11** (gradient: 5-95% ACN in 25 min using A: 0.1% HCOOH in water, B: ACN)

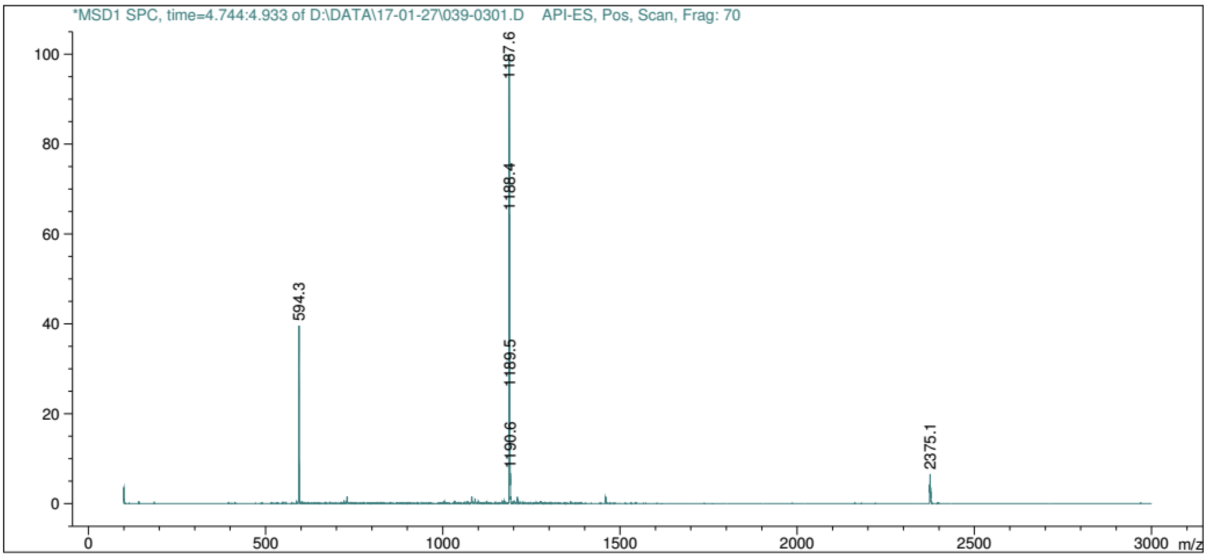

Fig. S45: ESI-MS from LC-MS of HPLC purified teixobactin analogue **11**. Exact mass calcd. for $C_{57}H_{94}N_{12}O_{15}$ = 1186.7, found M + H^+ = 1187.6, M/2 + H^+ = 594.3.

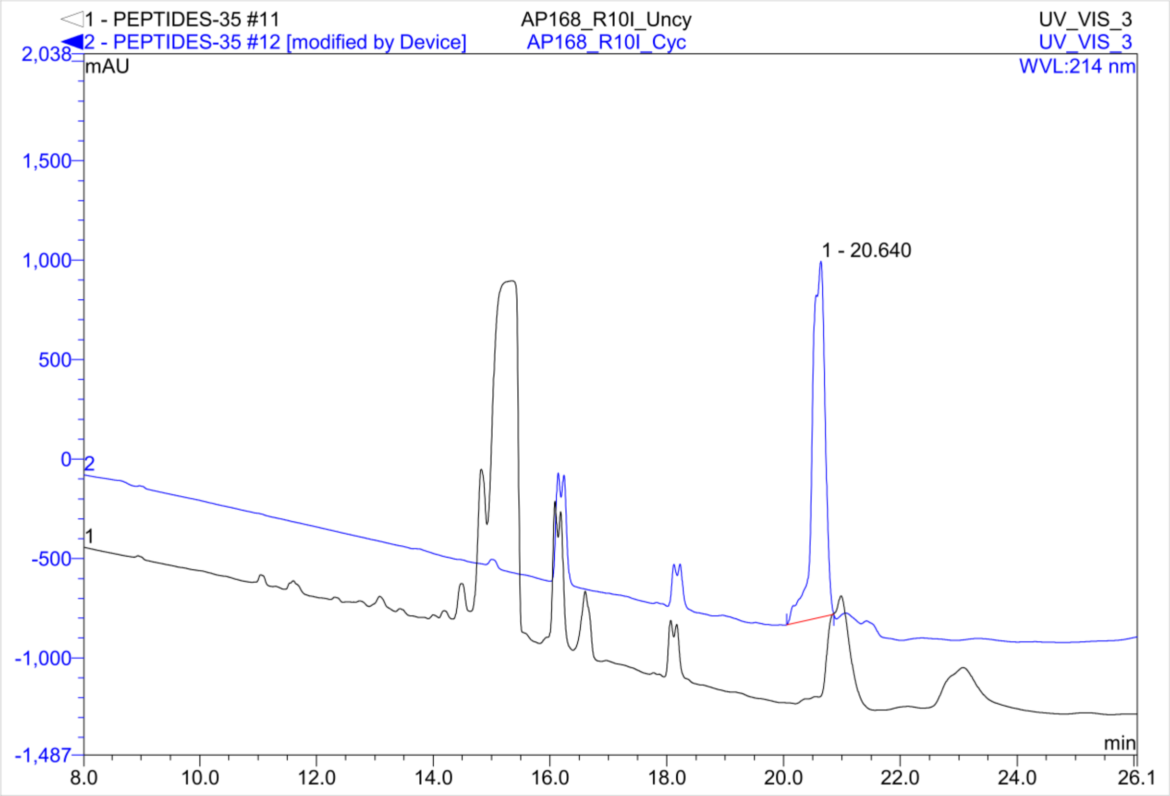

Fig. S46: HPLC trace showing the progress of cyclisation reaction for the synthesis of analogue **12** (i): conversion of the uncyclized protected teixobactin analogue t_R= 15.353 min (shown in black) to the cyclized protected teixobactin analogue t_R = 20.640 min (shown in blue) (Gradient: 5-95% in 25 min)

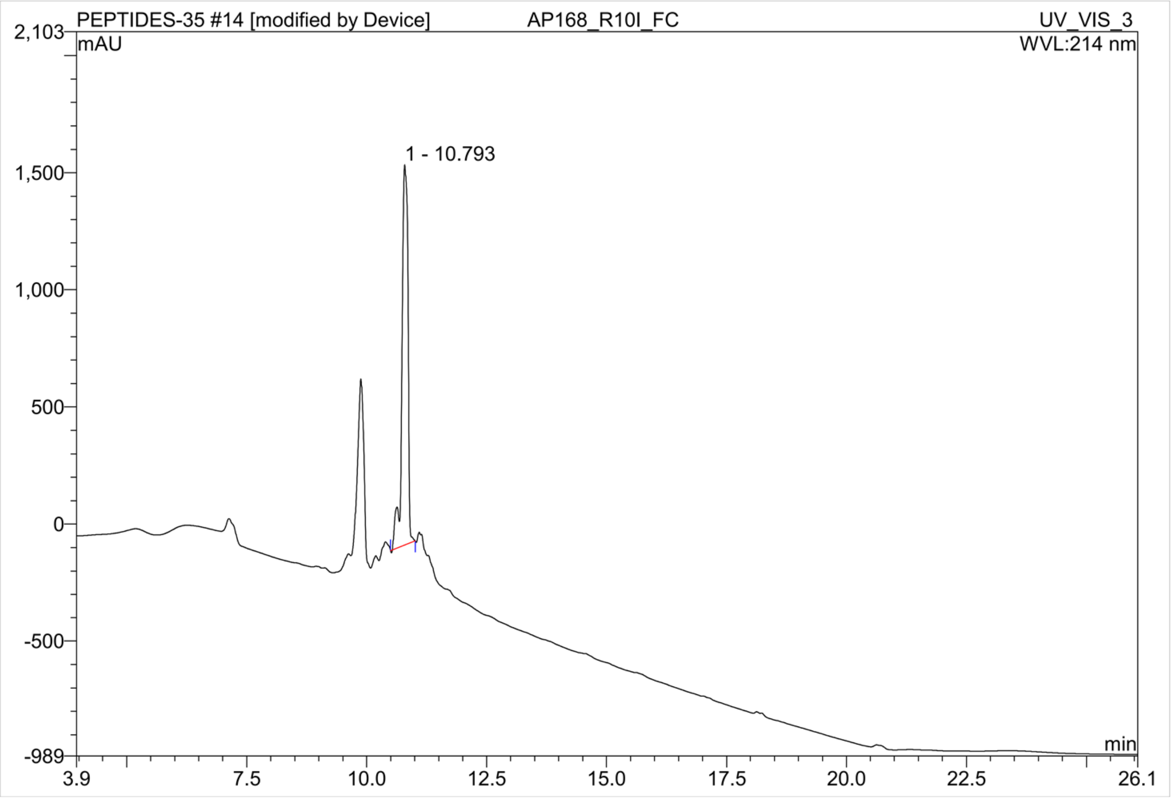

Fig. S47: HPLC trace of crude teixobactin analogue **12** t_R = 10.793 min (gradient: 5-95% ACN in 25 min using A: 0.1% HCOOH in water, B: ACN)

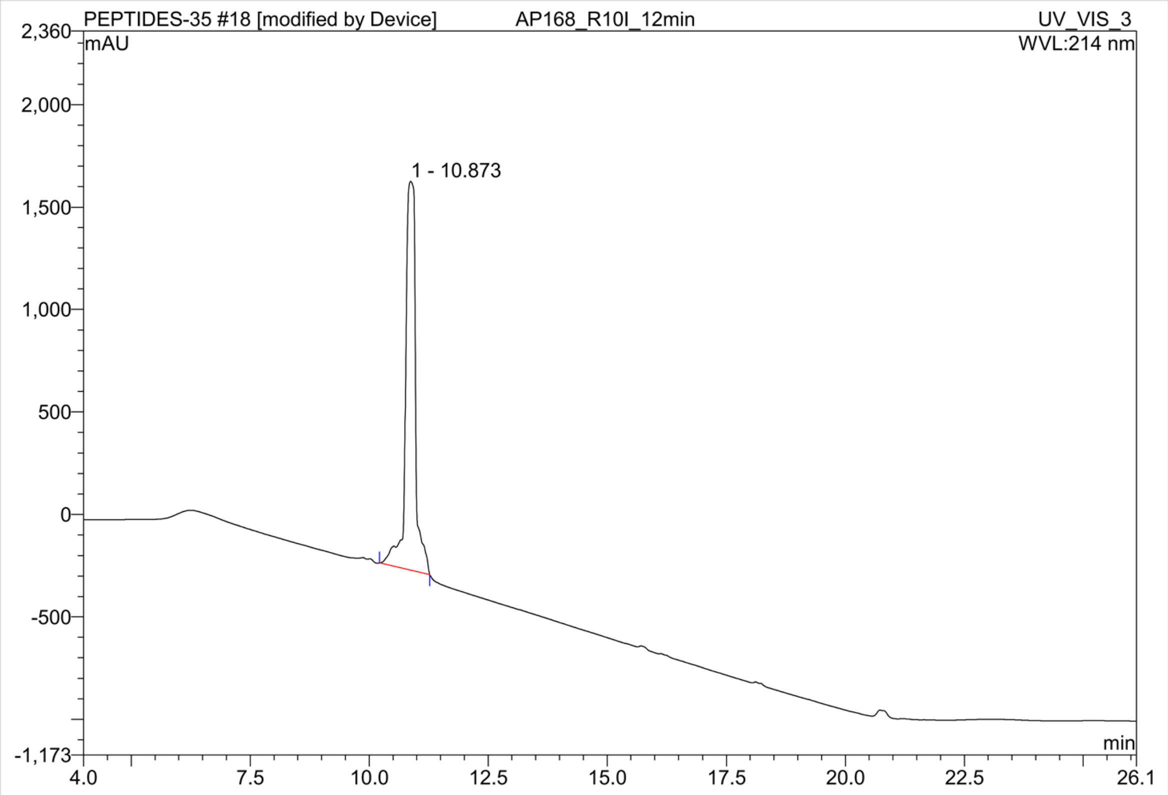

Fig. S48: HPLC trace of HPLC purified teixobactin analogue **12** (gradient: 5-95% ACN in 25 min using A: 0.1% HCOOH in water, B: ACN)

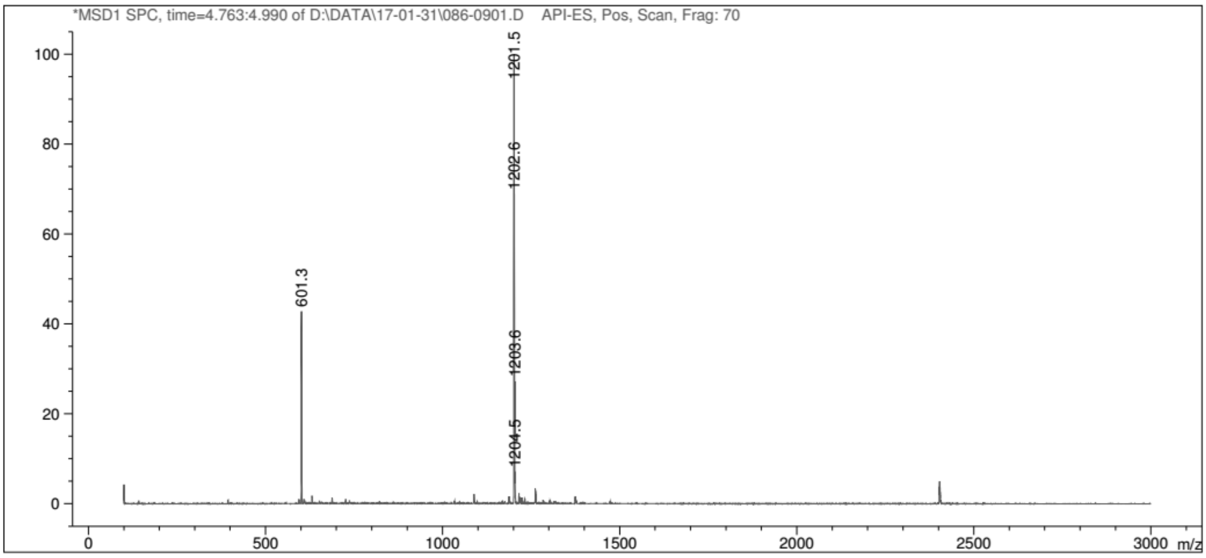

Fig. S49: ESI-MS from LC-MS of HPLC purified teixobactin analogue **12**. Exact mass calcd. for $C_{58}H_{96}N_{12}O_{15}$ = 1200.71, found M + H^+ = 1201.5, M/2 + H^+ = 601.3.

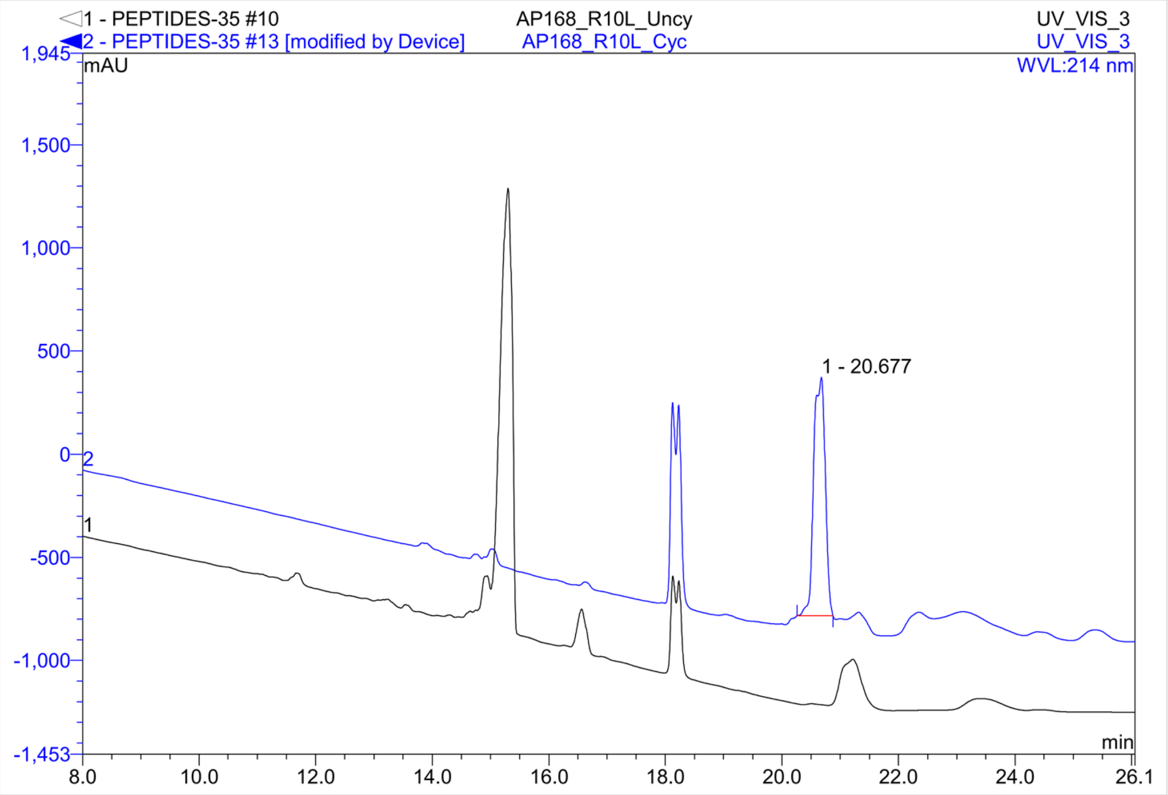

Fig. S50: HPLC trace showing the progress of cyclisation reaction for the synthesis of analogue **13** (i): conversion of the uncyclized protected teixobactin analogue t_R= 15.303 min (shown in black) to the cyclized protected teixobactin analogue t_R = 20.677 min (shown in blue) (Gradient: 5-95% in 25 min)

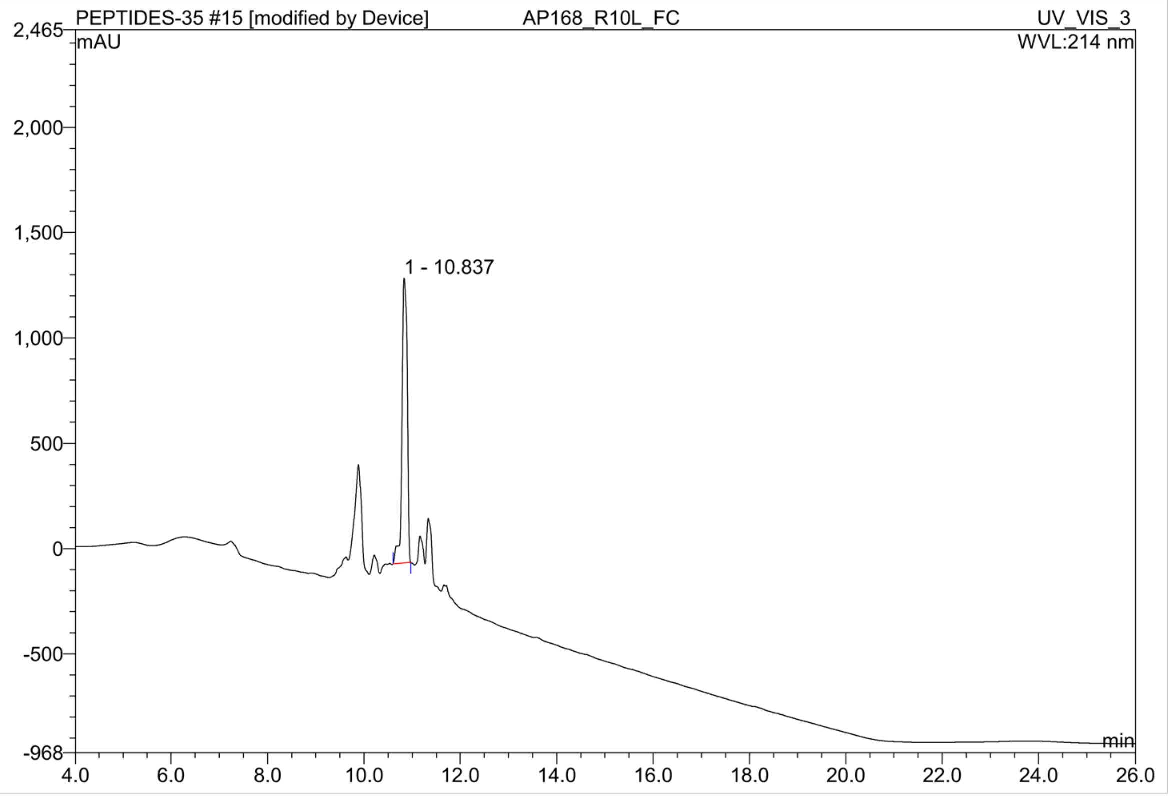

Fig. S51: HPLC trace of crude teixobactin analogue **13** t_R = 10.837 min (gradient: 5-95% ACN in 25 min using A: 0.1% HCOOH in water, B: ACN)

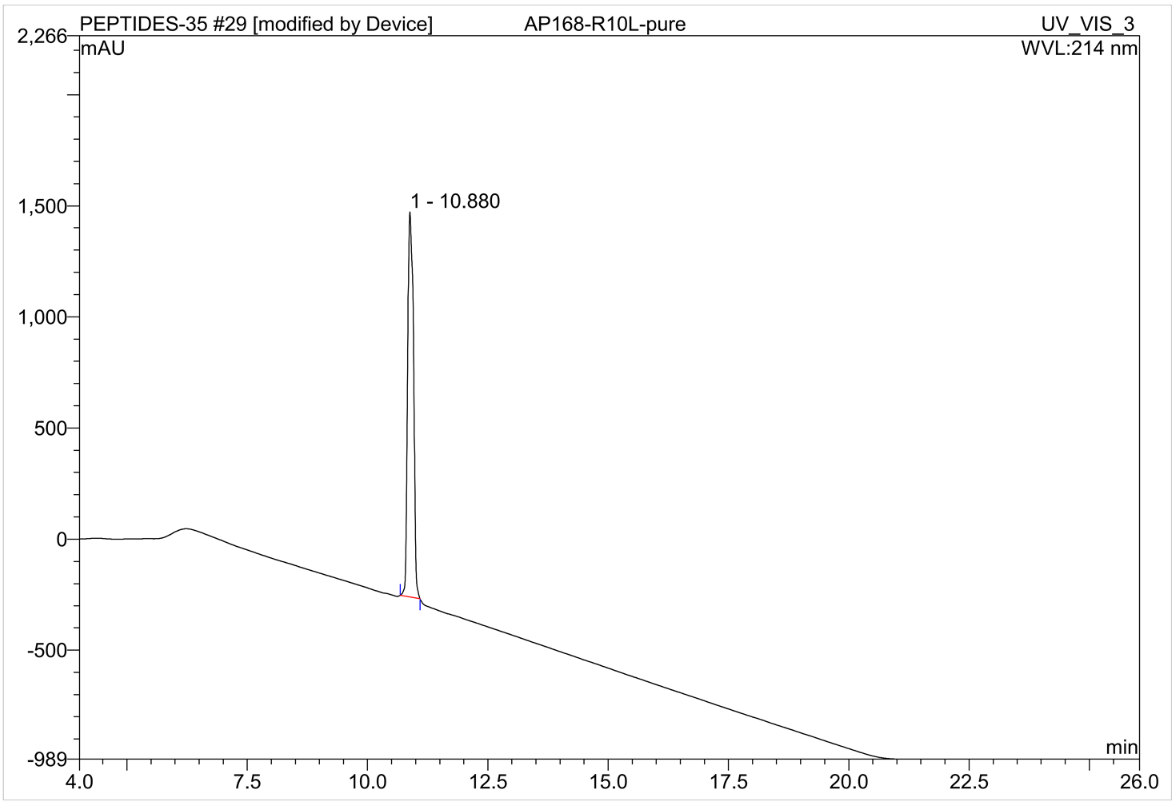

Fig. S52: HPLC trace of HPLC purified teixobactin analogue **13** (gradient: 5-95% ACN in 25 min using A: 0.1% HCOOH in water, B: ACN)

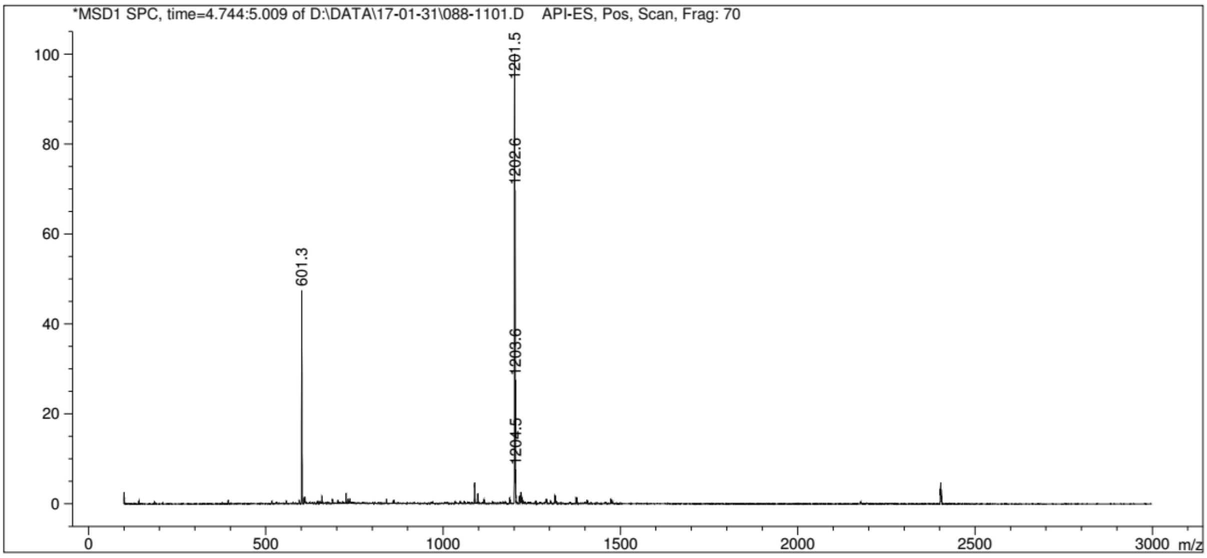

Fig. S53: ESI-MS from LC-MS of HPLC purified teixobactin analogue **13**. Exact mass calcd. for $C_{58}H_{96}N_{12}O_{15}$ = 1200.71, found M + H^+ = 1201.5, M/2 + H^+ = 601.3.

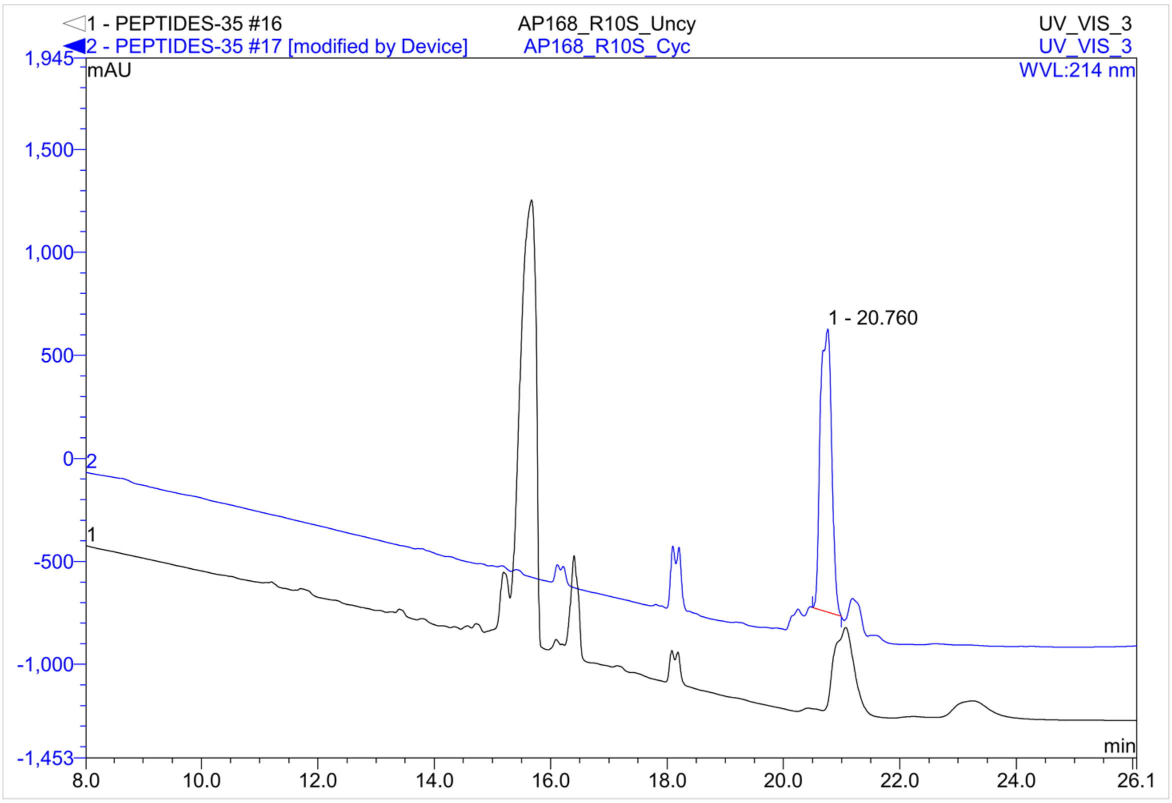

Fig. S54: HPLC trace showing the progress of cyclisation reaction for the synthesis of analogue **14** (i): conversion of the uncyclized protected teixobactin analogue t_R= 15.673 min (shown in black) to the cyclized protected teixobactin analogue t_R = 20.760 min (shown in blue) (Gradient: 5-95% in 25 min)

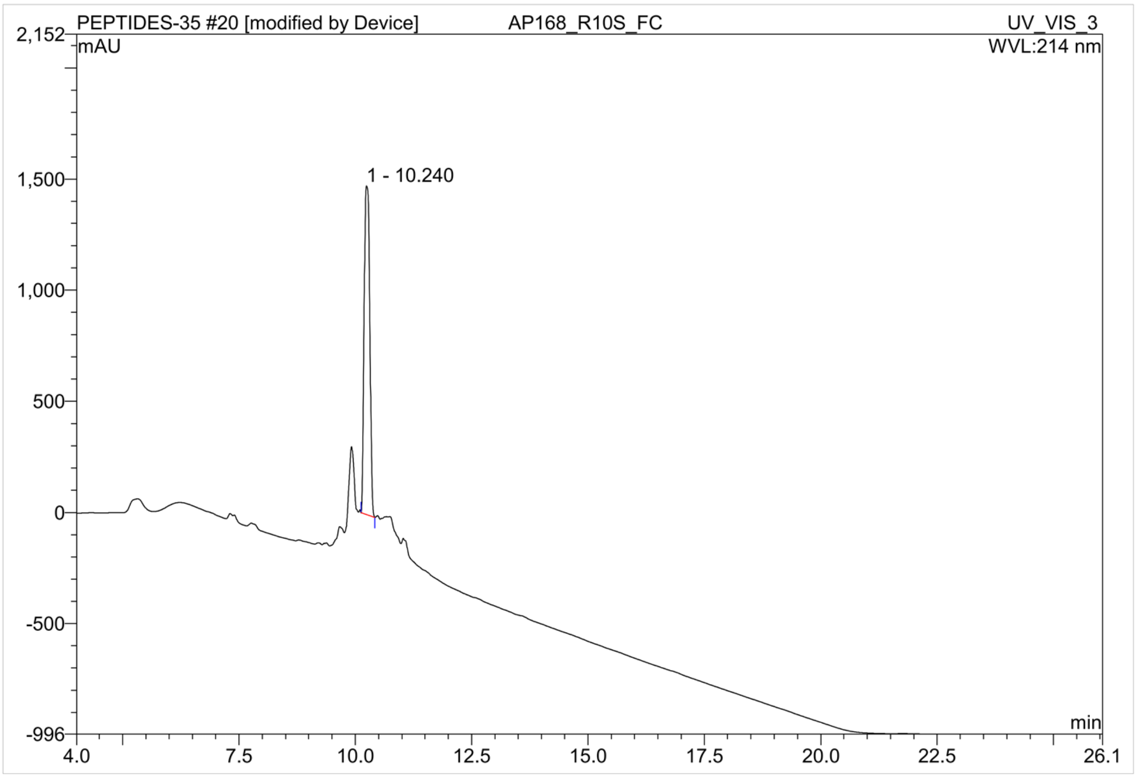

Fig. S55: HPLC trace of crude teixobactin analogue **14** t_R = 10.630 min (gradient: 5-95% ACN in 25 min using A: 0.1% HCOOH in water, B: ACN)

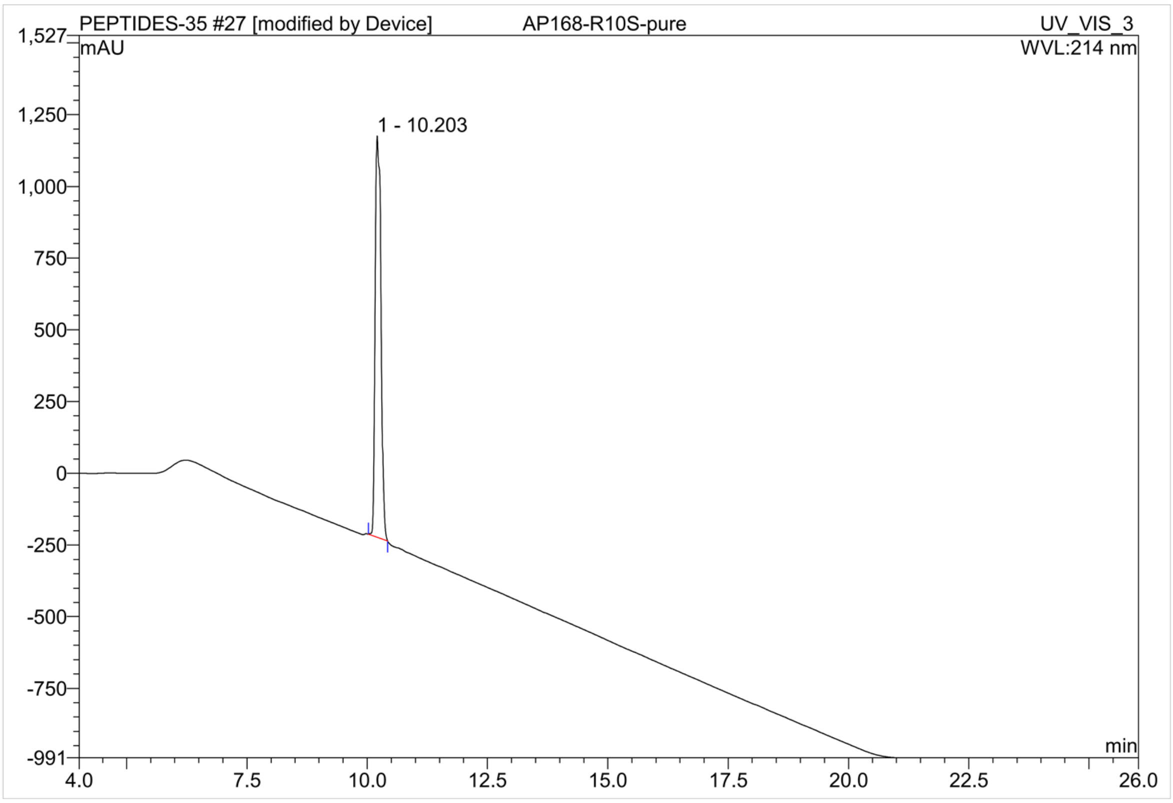

Fig. S56: HPLC trace of HPLC purified teixobactin analogue **14** (gradient: 5-95% ACN in 25 min using A: 0.1% HCOOH in water, B: ACN)

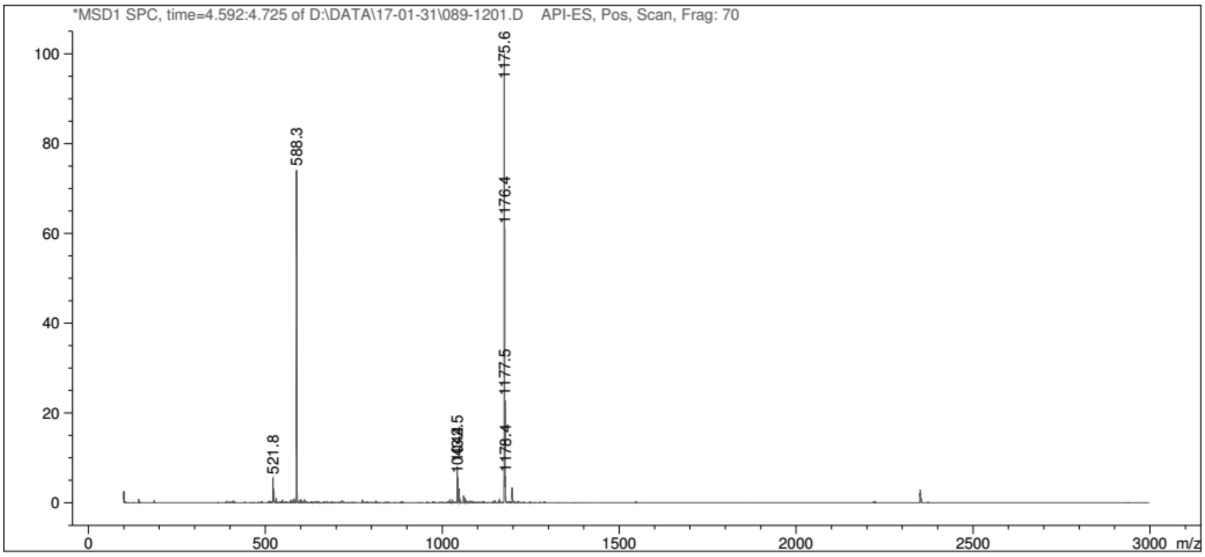

Fig. S57: ESI-MS from LC-MS of HPLC purified teixobactin analogue **14**. Exact mass calcd. for $C_{55}H_{90}N_{12}O_{16}$ = 1174.66, found M + H$^+$ = 1175.6, M/2 + H$^+$ = 588.3.

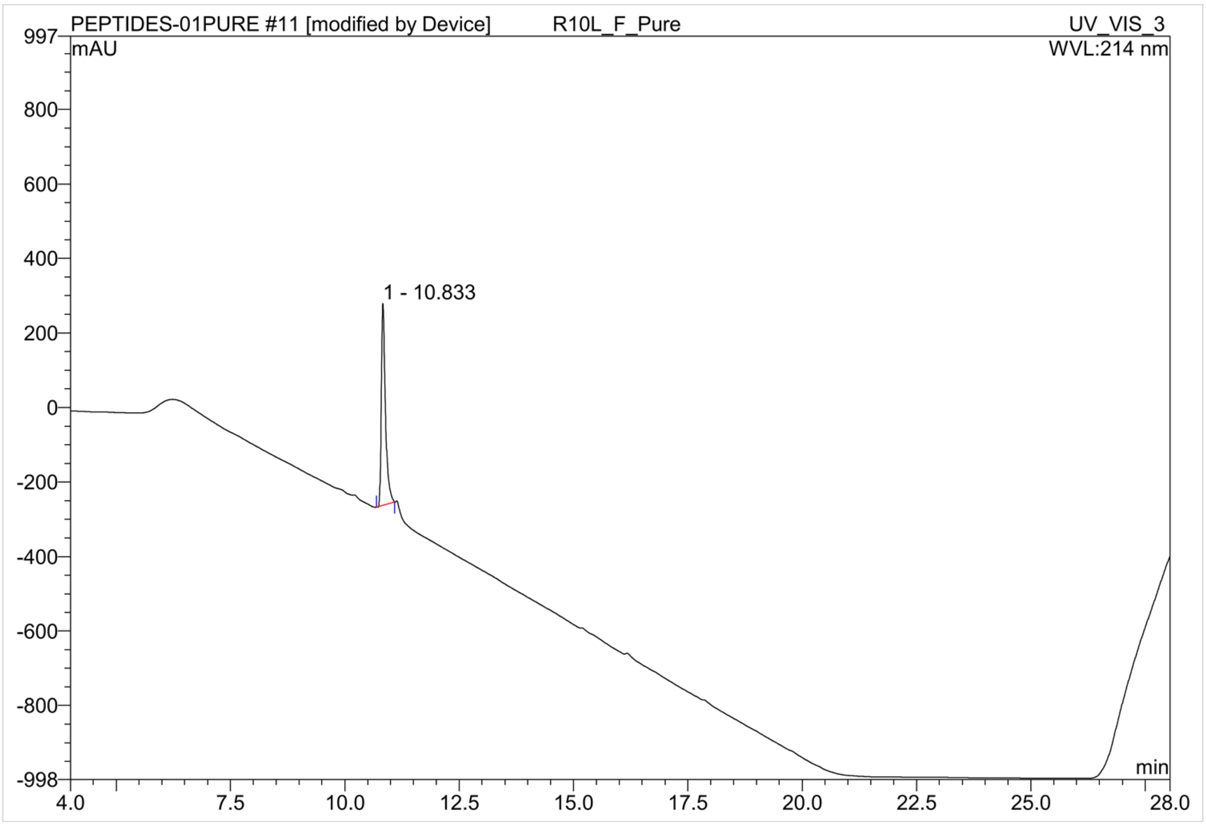

Fig. S58: HPLC trace of HPLC purified teixobactin analogue **15** (gradient: 5-95% ACN in 25 min using A: 0.1% HCOOH in water, B: ACN)

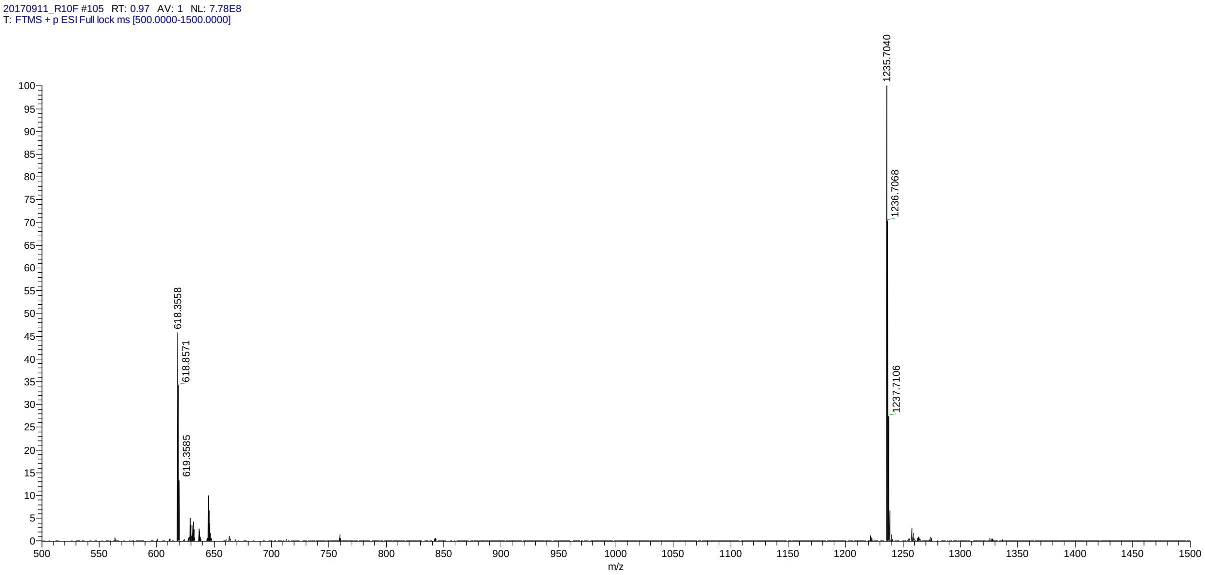

Fig. S59: HRMS of HPLC purified teixobactin analogue **15**. Exact mass calcd. for $C_{61}H_{94}N_{12}O_{15}$ = 1235.6962, found M = 1235.7040, M/2 + H^+ = 618.3558.

V. NMR Analysis

All NMR was carried out in DMSO-d_6 at 27°C on a Bruker Avance III HD 500 MHz spectrometer equipped with a room-temperature broadband probe. The following spectra were utilised in the assignment of 1 mM solutions of the teixobactin mutants: ^{1}H (128k points, 16 scans); $^{13}C\{1H\}$ (64k points, 1024 scans); ^{1}H- ^{13}C HSQC (2k and 256 points in the direct and indirect dimensions, 4 scans); ^{1}H- ^{13}C HMBC (2k and 512 points, 8 scans); ^{1}H- ^{1}H TOCSY (2k and 192 points; 32 scans); and ^{1}H- ^{1}H NOESY (2k and 192 points, 48 scans). Spectral analysis was carried out using CCPNMR Analysis.[2] NOE-derived distance restraints obtained from the NOESY spectra were used in structural calculations using Cyana 2.1[3] prior to energy minimisation using Gromacs 5.1[4] and RSFF2 forcefield.[5] Geranyl pyrophosphate titrations were carried out using 0.5 mM teixobactin mutants and the following molar equivalents of geranyl pyrophosphate: 0.0, 0.1, 0.2, 0.4, 0.6, 0.8, 1.0, 1.25 and 1.5. Geranyl pyrophosphate was freeze-dried and dissolved in MeOH:D_2O (7:3, 1 mM concentration) for the titration experiments. ^{1}H- ^{1}H TOCSY spectra were acquired and assigned at each titration point to yield accurate chemical shift perturbations (CSPs). Residue-specific binding isotherms obtained from Hα CSPs were fit using the Hill equation with a Hill coefficient of 1.0 or > 1.0 in the case of sigmoidal curves. (Hα resonances were chosen for CSP analysis because they are common to each residue and because they were resolved for the majority of residues.) Full spectrum analysis of the titration was carried out using TREND[6], which uses principal component analysis (PCA) to give an overall binding isotherm free from the influence of intermediate exchange.

			**1**	**2**	**3**	**4**	**5**	**6**	**7**	**10**
1	D-NmPhe	HNm								2.165
1	D-NmPhe	H	8.103							
1	D-NmPhe	Hα	4.339	3.184	3.244	3.259	3.258	3.262	3.264	3.278
1	D-NmPhe	Hβ	**1.190**	2.680<br>2.805	2.693<br>2.775	2.690<br>2.782	2.690<br>2.786	2.692<br>2.791	2.690<br>2.789	2.711<br>2.782
1	D-NmPhe	Hδ*			7.190	7.180				7.184
1	D-NmPhe	Hε*								7.240
1	D-NmPhe	Hζ								7.175
2	Ile	H	8.045	8.046	7.898	7.943	7.933	7.946	7.944	7.950
2	Ile	Hα	4.215	4.313	4.146	4.215	4.185	4.199	4.197	4.198
2	Ile	Hβ	1.798	**1.123**	1.648	1.686	1.675	1.679	1.679	1.664
2	Ile	Hγ1	1.109<br>1.390		0.901<br>1.226	0.913<br>1.237	0.902<br>1.243	0.907<br>1.250	0.906<br>1.250	0.907<br>1.234
2	Ile	Hγ2*	0.832		0.735	0.745	0.739	0.734	0.736	0.733
2	Ile	Hδ1*	0.832		0.735	0.745	0.739	0.734	0.736	0.733
3	Ser	H	7.968	7.964	8.017	7.971	7.974	7.946	7.949	7.918
3	Ser	Hα	4.286	4.276	4.309	4.284	4.293	4.305	4.299	4.316
3	Ser	Hβ	3.588<br>3.862	3.566<br>3.614	**1.218**	3.534<br>3.604	3.542<br>3.627	3.534<br>3.608	3.534<br>3.606	3.544<br>3.598
3	Ser	Hγ	5.003	5.022		4.987	5.020	4.999	5.039	4.958
4	D-Gln	H	7.820	8.025	7.989	7.884	7.938	7.922	7.966	7.958
4	D-Gln	Hα	4.287	4.290	4.313	4.332	4.270	4.292	4.299	4.306
4	D-Gln	Hβ	2.050<br>2.119	2.093<br>2.093	2.072<br>2.072	**1.182**	2.087<br>2.087	2.068<br>2.068	2.086<br>2.086	2.087<br>2.094
4	D-Gln	Hγ	1.712<br>1.888	1.734<br>1.898	1.694<br>1.871		1.699<br>1.898	1.688<br>1.858	1.687<br>1.869	1.704<br>1.872
4	D-Gln	Hε2	6.782	6.783	6.760		6.760	6.747	6.798	6.789

			7.214	7.220	7.260		7.219	7.204	7.235	7.247
5	D-Ile	H	7.710	7.680	7.749	7.766	8.015	7.801	7.824	7.712
5	D-Ile	Hα	4.379	4.400	4.380	4.265	4.326	4.193	4.244	4.367
5	D-Ile	Hβ	1.779	1.788	1.783	1.728	**1.209**	1.690	1.721	1.772
5	D-Ile	Hγ1	1.062<br>1.327	1.074<br>1.295	1.073<br>1.299	1.051<br>1.396		1.037<br>1.404	1.045<br>1.409	1.074<br>1.317
5	D-Ile	Hγ2*	0.774	0.765	0.775	0.806		0.787	0.804	0.782
5	D-Ile	Hδ1*	0.774	0.765	0.775	0.806		0.787	0.804	0.784
6	Ile	H	7.957	7.929	7.918	7.889	7.807	8.074	7.992	7.970
6	Ile	Hα	4.269	4.286	4.292	4.280	4.314	4.405	4.191	4.160
6	Ile	Hβ	1.780	1.776	1.776	1.759	1.747	**1.232**	1.778	1.757
6	Ile	Hγ1	1.104<br>1.418	1.090<br>1.408	1.092<br>1.411	1.087<br>1.408	1.072<br>1.402		1.135<br>1.414	1.150<br>1.416
6	Ile	Hγ2*	0.821	0.816	0.824	0.815	0.810		0.814	0.822
6	Ile	Hδ1*	0.821	0.816	0.819	0.815	0.810		0.814	0.822
7	Ser	H	9.087	9.207	9.188	9.149	9.282	9.565	8.521	8.607
7	Ser	Hα	4.385	4.404	4.402	4.394	4.413	4.279	4.336	4.233
7	Ser	Hβ	3.706<br>3.768	3.703<br>3.775	3.701<br>3.775	3.694<br>3.767	3.711<br>3.773	3.783<br>3.820	**1.335**	3.693<br>3.694
7	Ser	Hγ	5.652	5.671	5.674	5.665	5.646	5.718		
8	D-Thr	H	8.717	8.856	8.865	8.897	8.847	8.679	8.905	7.987
8	D-Thr	Hα	4.639	4.655	4.650	4.642	4.649	4.647	4.554	4.568
8	D-Thr	Hβ	5.363	5.366	5.362	5.360	5.363	5.351	5.330	5.355
8	D-Thr	Hγ2*	1.105	1.097	1.097	1.094	1.097	1.059	1.112	1.136
9	Ala	H	8.217	8.203	8.210	8.210	8.193	8.103	8.204	8.101
9	Ala	Ha	3.932	3.931	3.933	3.933	3.931	3.908	3.992	3.950
9	Ala	Hβ*	1.294	1.280	1.283	1.282	1.286	1.248	1.351	1.340
10	Arg	H	8.156	8.219	8.235	8.233	8.206	8.349	8.274	7.687
10	Arg	Hα	4.275	4.274	4.280	4.273	4.285	4.271	4.265	4.395
10	Arg	Hβ	1.633<br>1.767	1.640<br>1.762	1.641<br>1.798	1.647<br>1.765	1.623<br>1.766	1.585<br>1.740	1.722<br>1.795	**1.336**
10	Arg	Hγ	1.429<br>1.493	1.418<br>1.487	1.420<br>1.488	1.416<br>1.490	1.420<br>1.484	1.406<br>1.479	1.438<br>1.500	
10	Arg	Hδ	3.124<br>3.124	3.115<br>3.115	3.127<br>3.127	3.109<br>3.109	3.119<br>3.119	3.112<br>3.112	3.117<br>3.117	
10	Arg	Hε	7.693	7.733	7.735	7.750	7.745	7.634	7.911	
10	Arg	Hη1			7.115<br>7.115	7.125<br>7.125				
10	Arg	Hη2			7.248<br>7.248	7.125<br>7.125				
11	Ile	H	8.390	8.499	8.502	8.495	8.48	8.495	8.104	7.562
11	Ile	Hα	4.033	4.030	4.033	4.033	4.033	4.036	4.054	4.027
11	Ile	Hβ	1.686	1.681	1.675	1.668	1.682	1.609	1.712	1.680
11	Ile	Hγ1	1.092<br>1.434	1.086<br>1.423	1.093<br>1.414	1.091<br>1.422	1.103<br>1.432	1.071<br>1.406	1.113<br>1.416	1.100<br>1.448
11	Ile	Hγ2*	0.811	0.798	0.806	0.814	0.800	0.763	0.814	0.829
11	Ile	Hδ1*	0.811	0.798	0.806	0.814	0.800	0.763	0.814	0.830

Table S2. Proton chemical shifts obtained from the mutants used in this study. The residue replaced by alanine is shown with a grey background, with the introduced methyl group shown in bold.

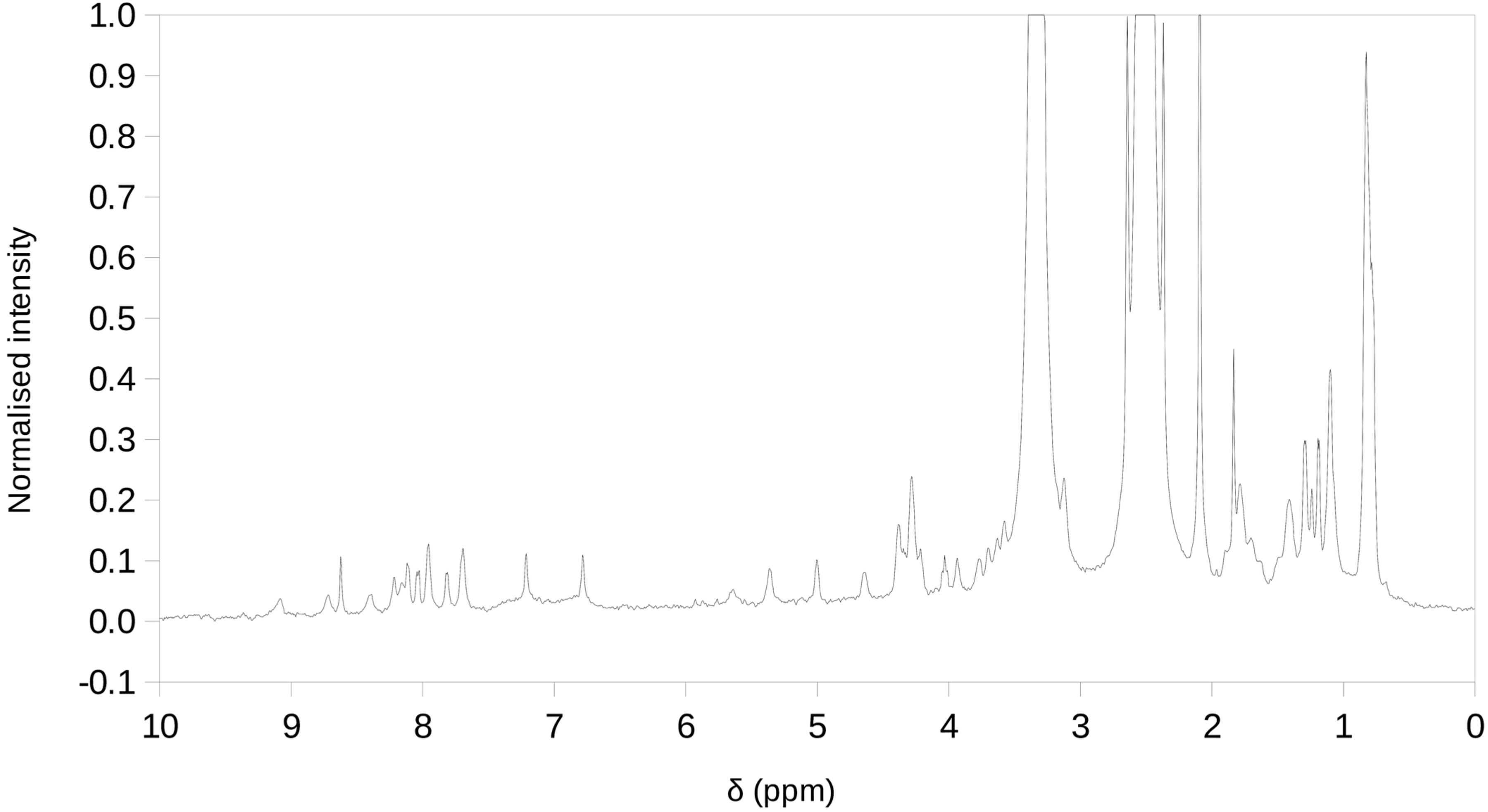

Fig. S60 ^{1}H NMR spectra obtained from compound **1**

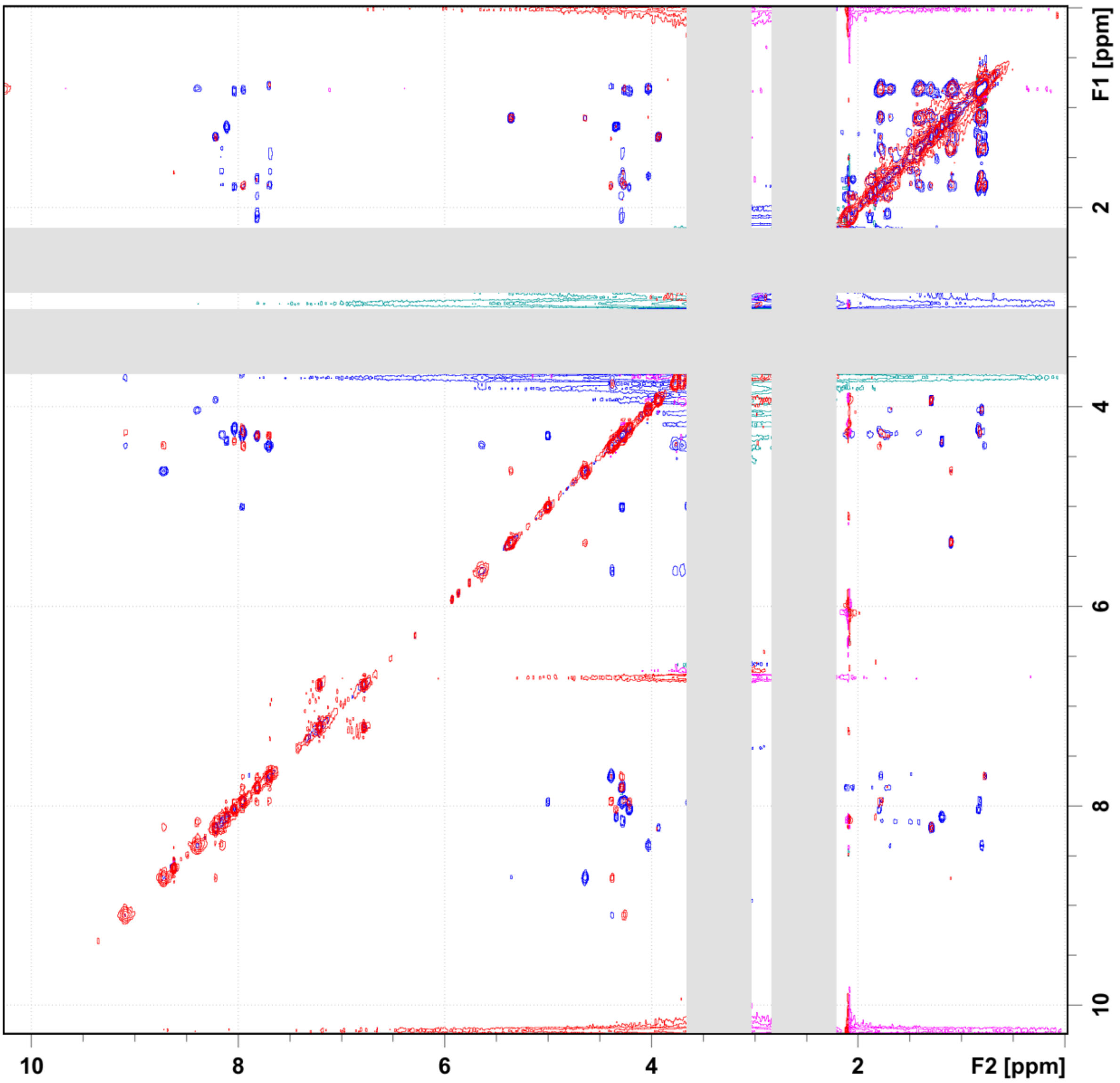

Fig. S61 ^{1}H-^{1}H TOCSY (blue) and ^{1}H-^{1}H NOESY (red) spectra obtained from compound **1**. For clarity, grey boxes obscure solvent signals.

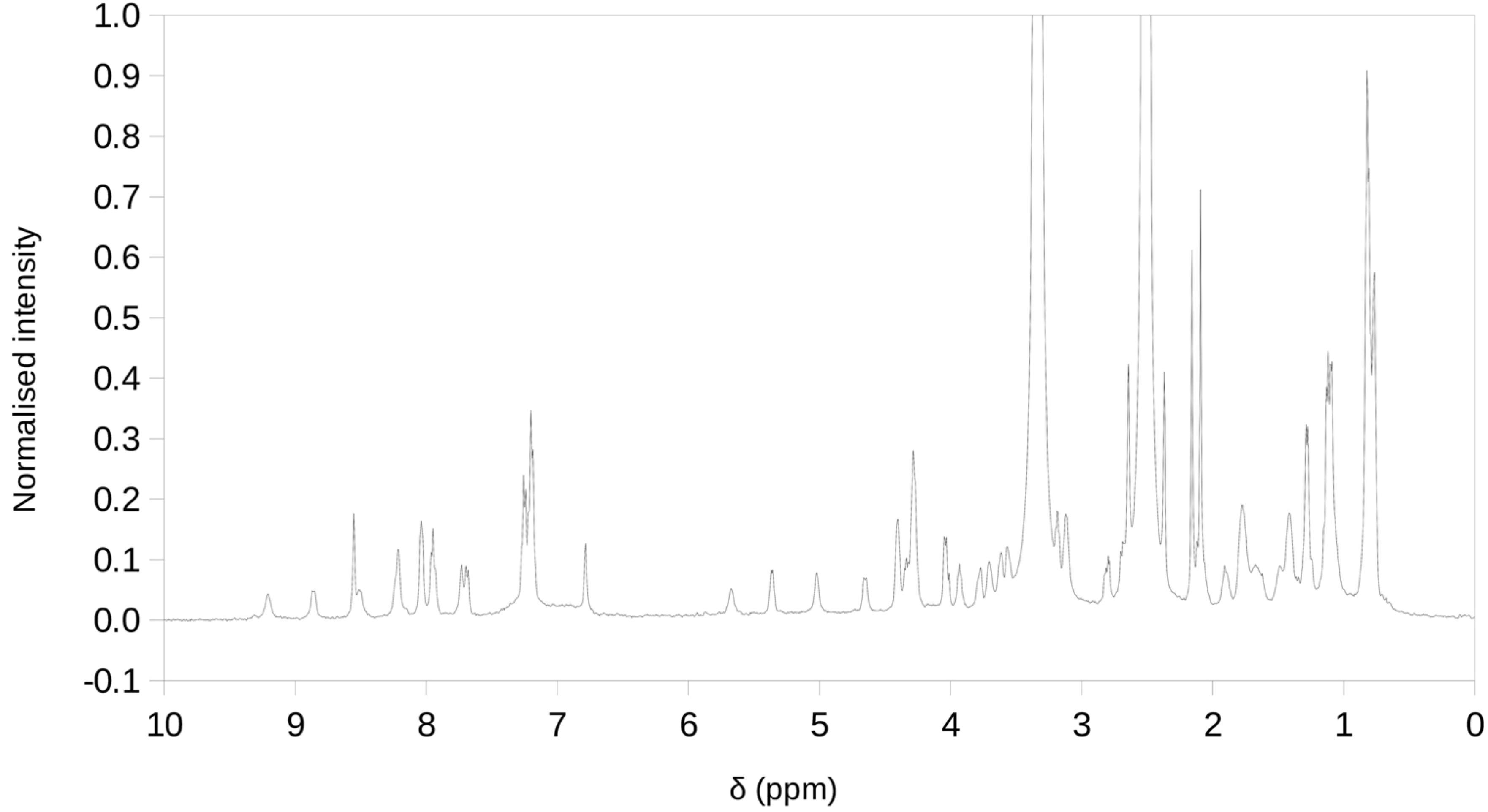

Fig. S62 ^{1}H NMR spectra obtained from compound **2**

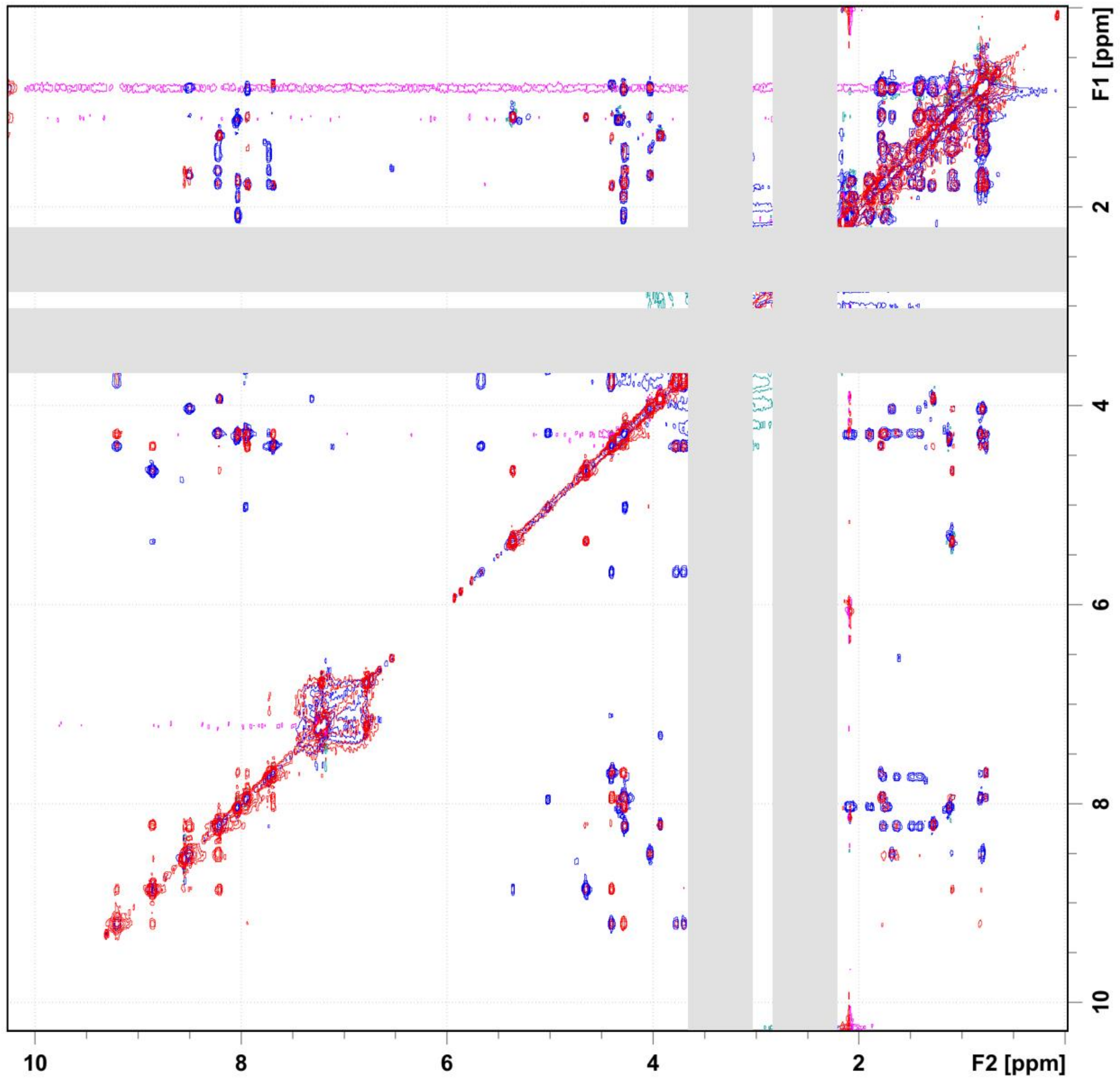

Fig. S63 ^{1}H-^{1}H TOCSY (blue) and ^{1}H-^{1}H NOESY (red) spectra obtained from compound **2**. For clarity, grey boxes obscure solvent signals.

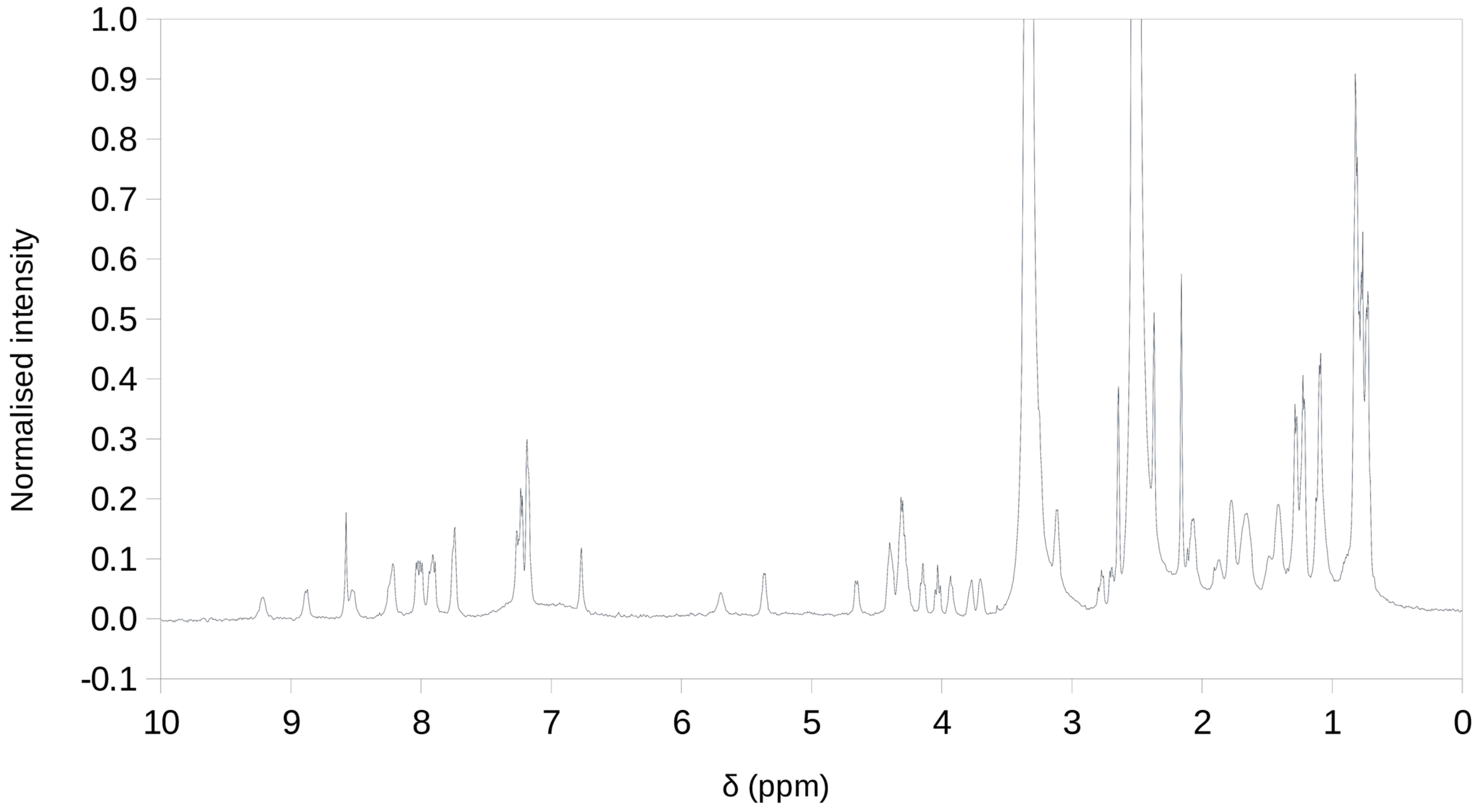

Fig. 64 ^{1}H NMR spectra obtained from compound **3**

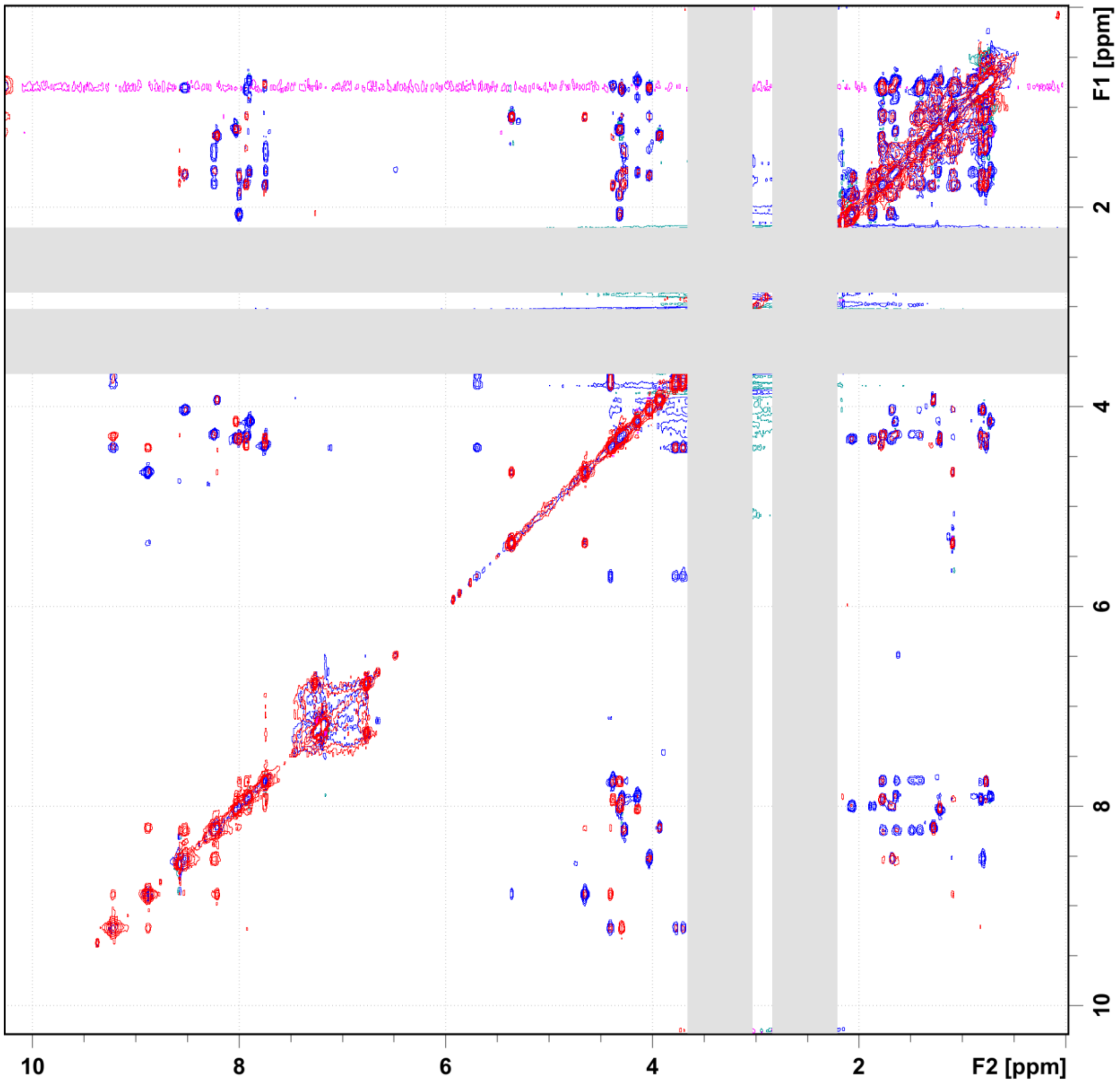

Fig. S65 ^{1}H-^{1}H TOCSY (blue) and ^{1}H-^{1}H NOESY (red) spectra obtained from compound **3**. For clarity, grey boxes obscure solvent signals.

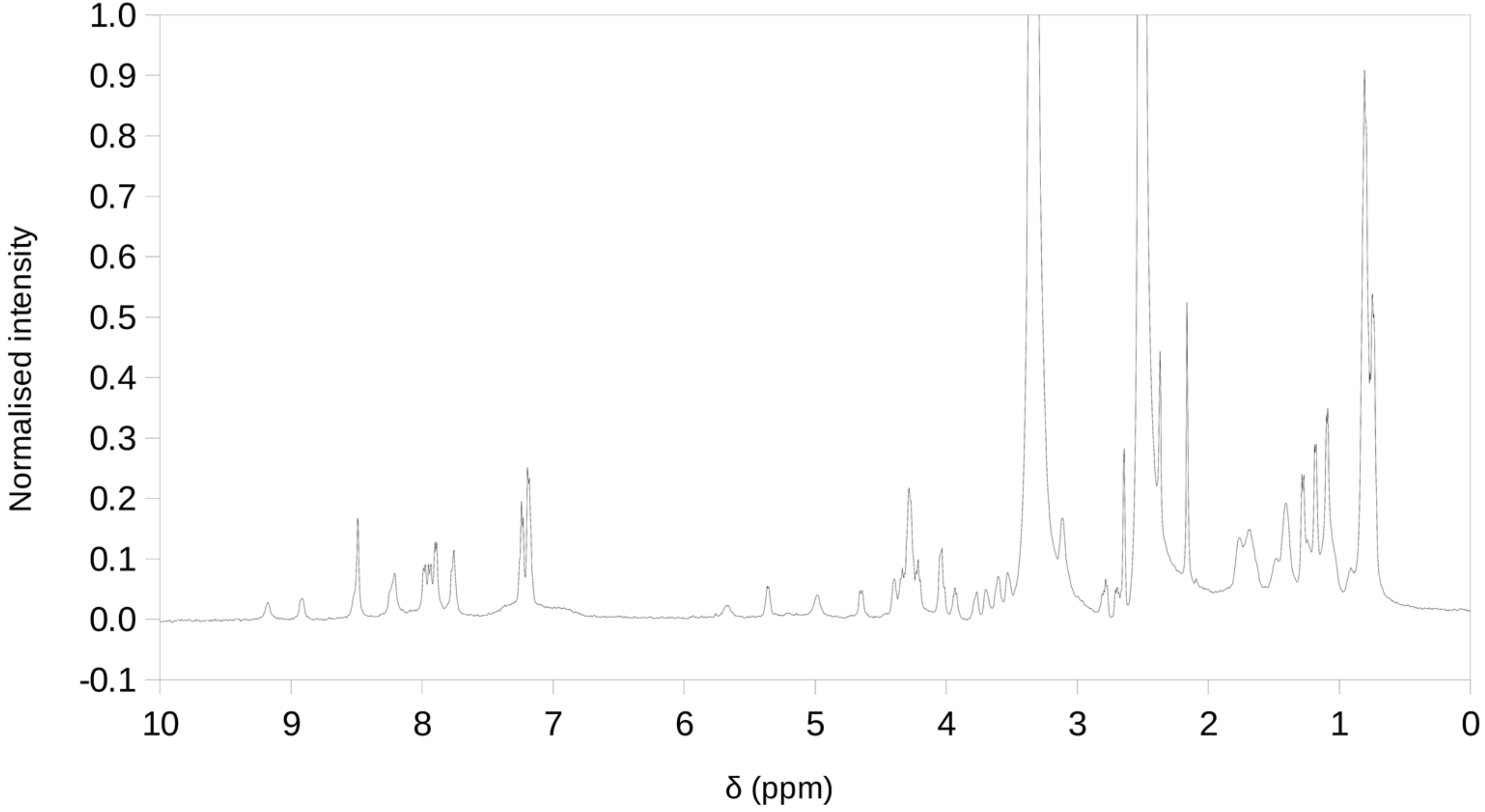

Fig. 66 ^{1}H NMR spectra obtained from compound **4**

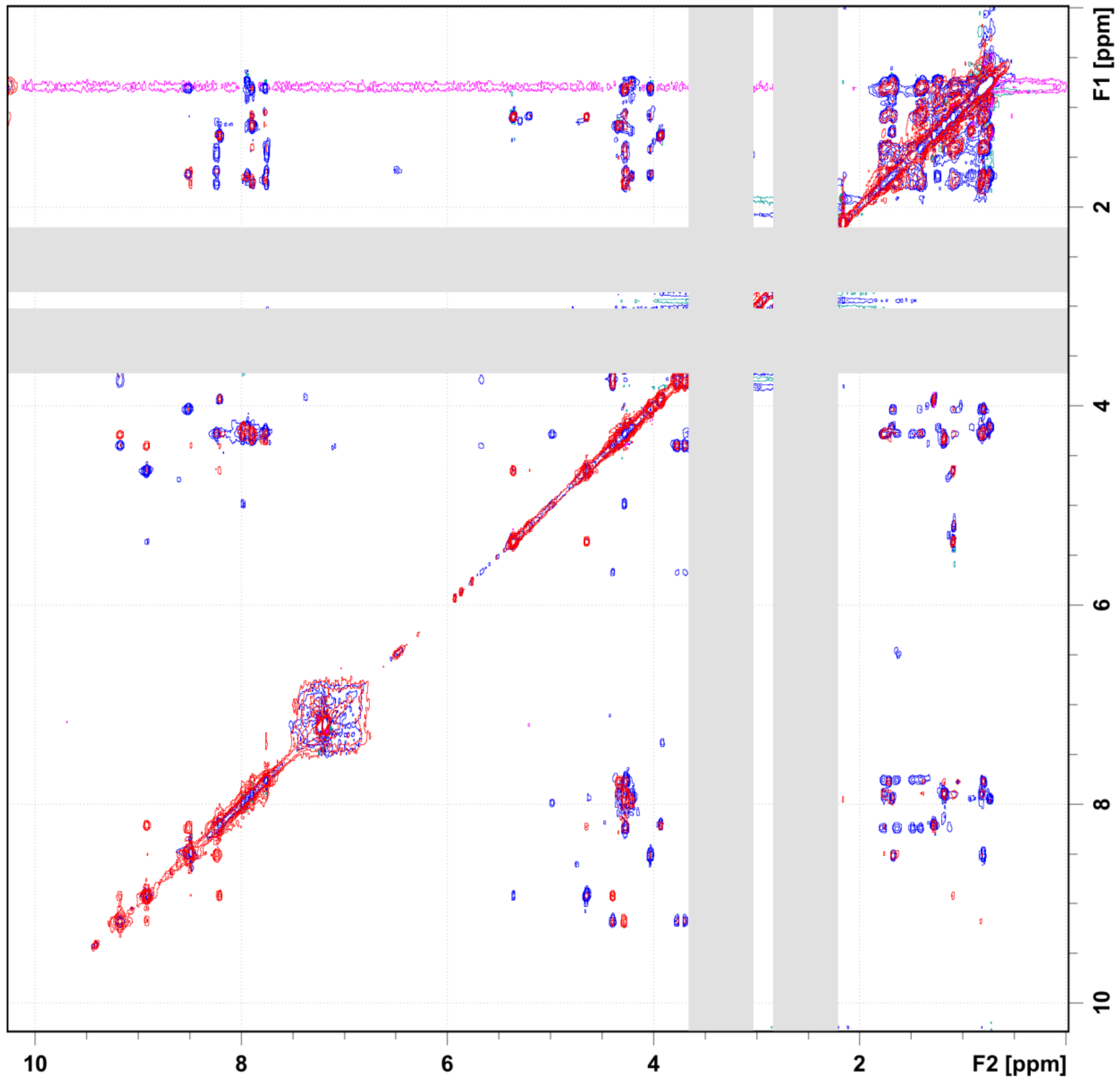

Fig. S67 ^{1}H-^{1}H TOCSY (blue) and ^{1}H-^{1}H NOESY (red) spectra obtained from compound **4**. For clarity, grey boxes obscure solvent signals.

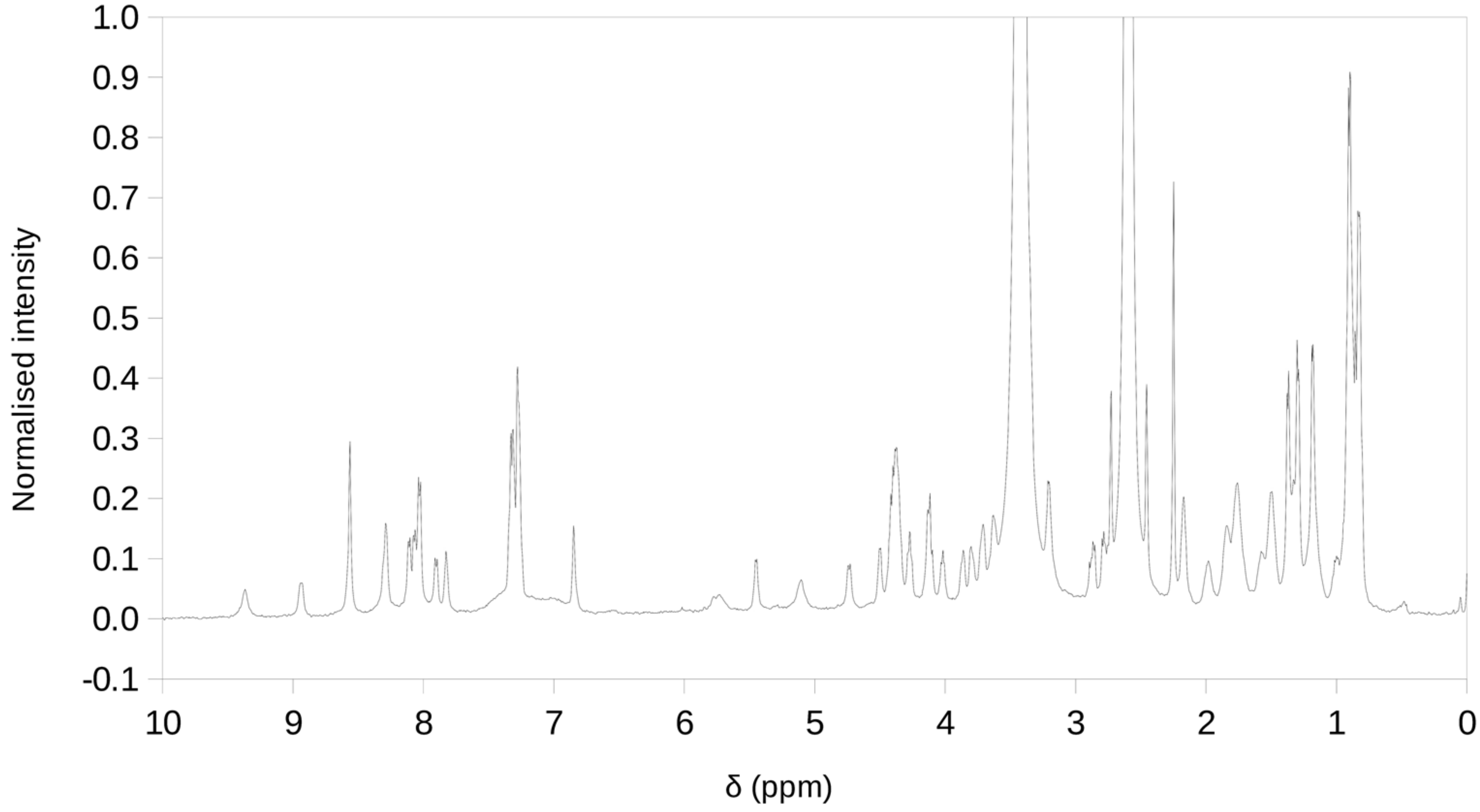

Fig. 68 ^{1}H NMR spectra obtained from compound **5**

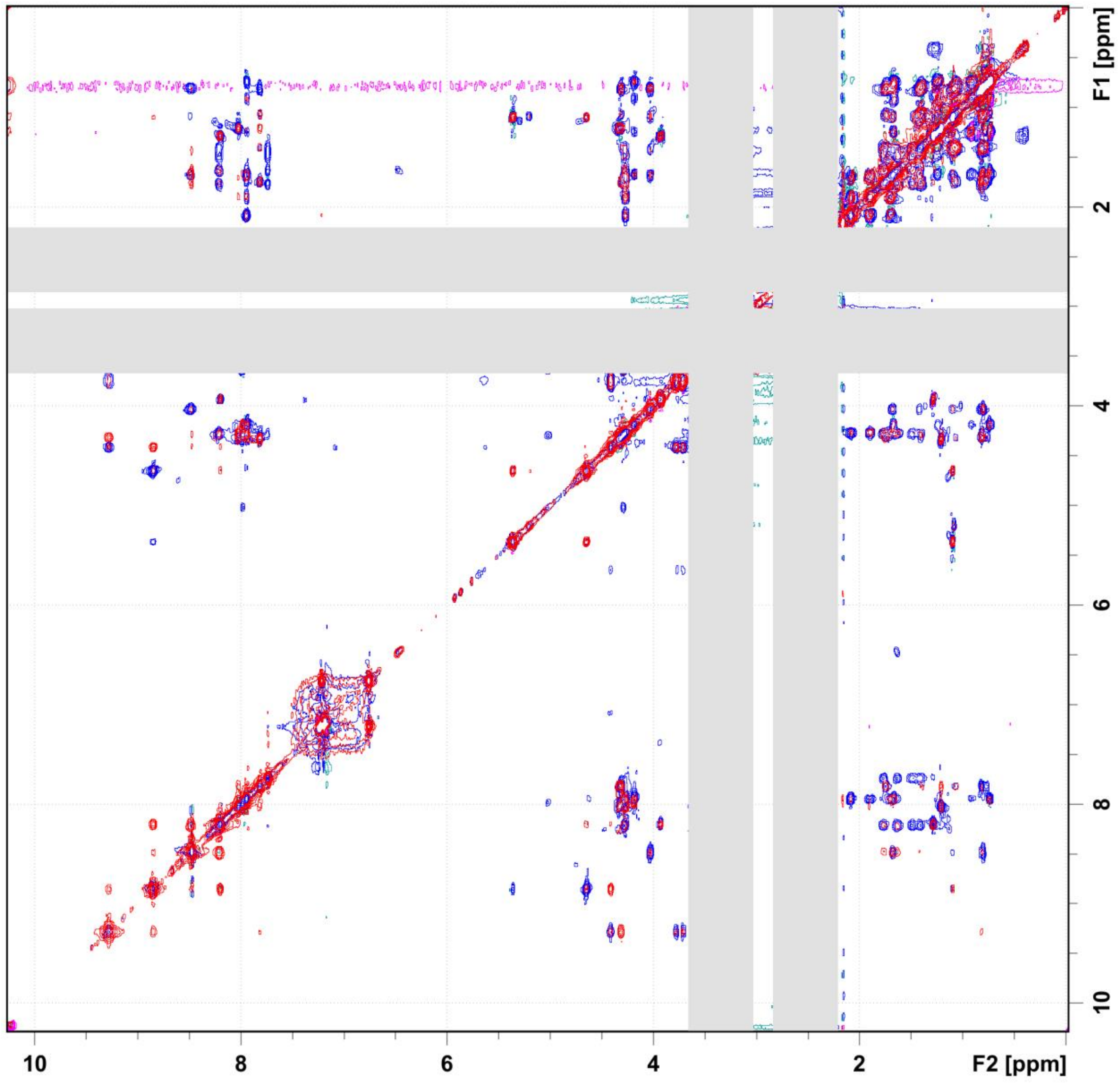

Fig. S69. ^{1}H-^{1}H TOCSY (blue) and ^{1}H-^{1}H NOESY (red) spectra obtained from compound **5**. For clarity, grey boxes obscure solvent signals.

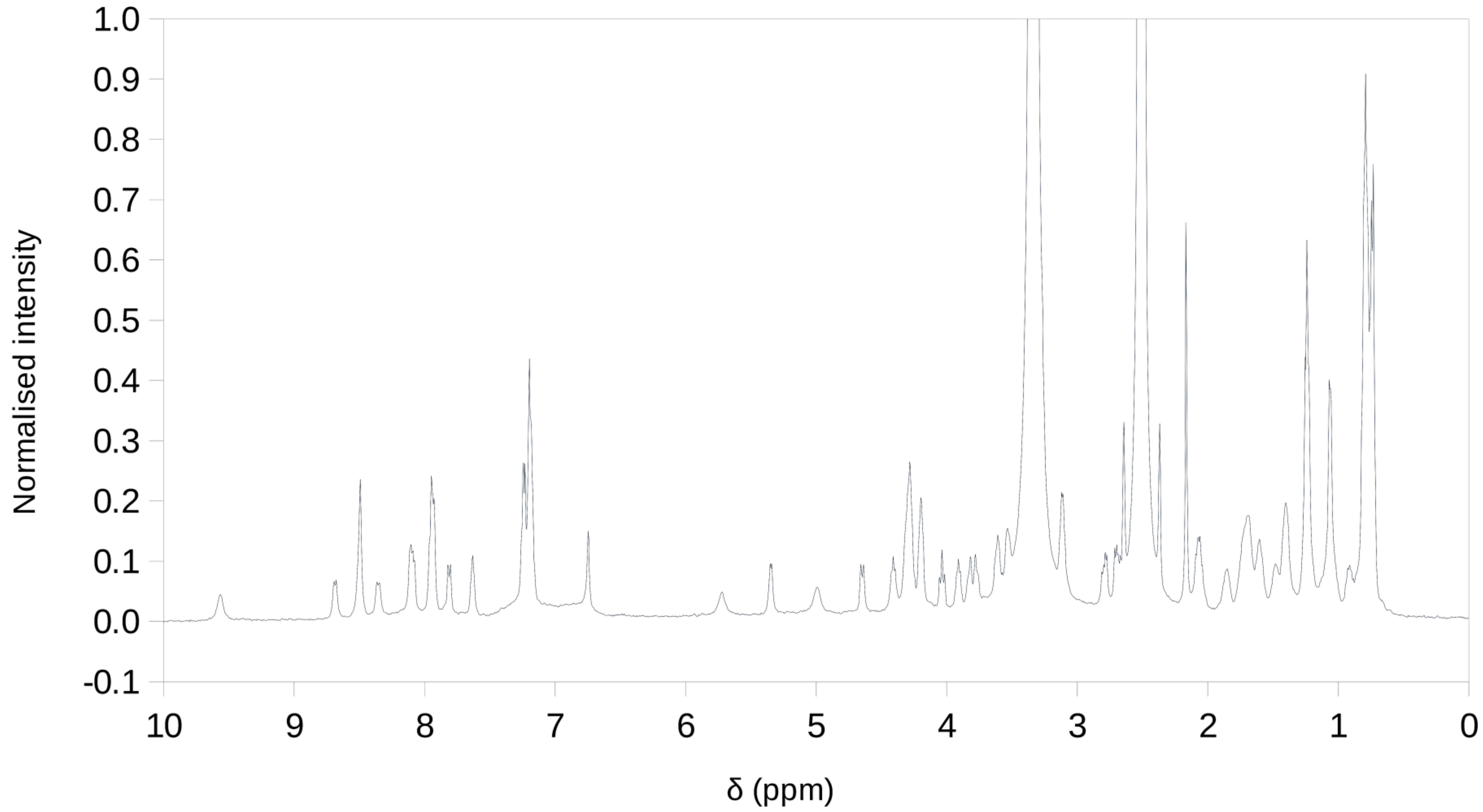

Fig. 70 ^{1}H NMR spectra obtained from compound **6**

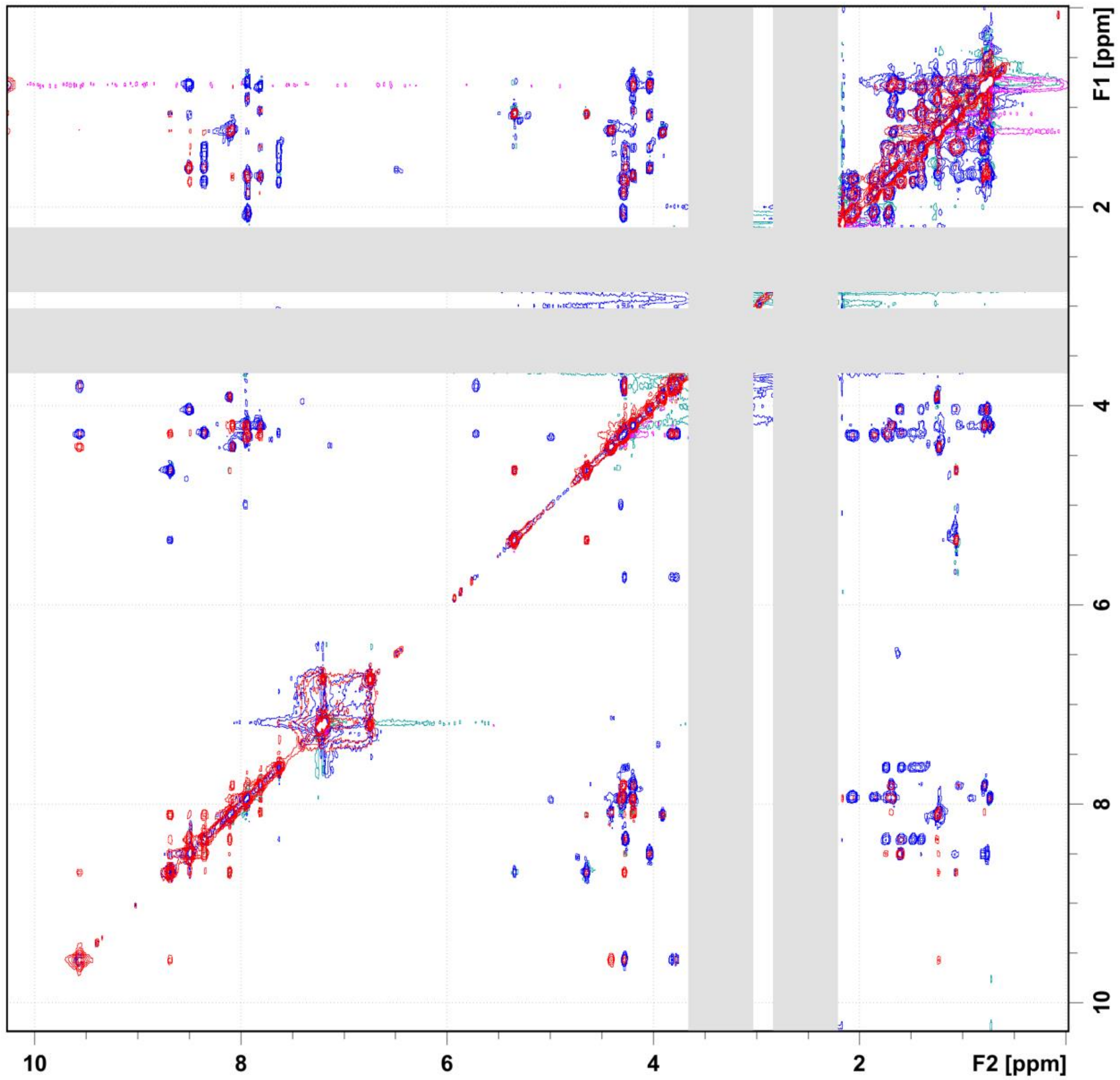

Fig. S71 ^1H-^1H TOCSY (blue) and ^1H-^1H NOESY (red) spectra obtained from compound **6**. For clarity, grey boxes obscure solvent signals.

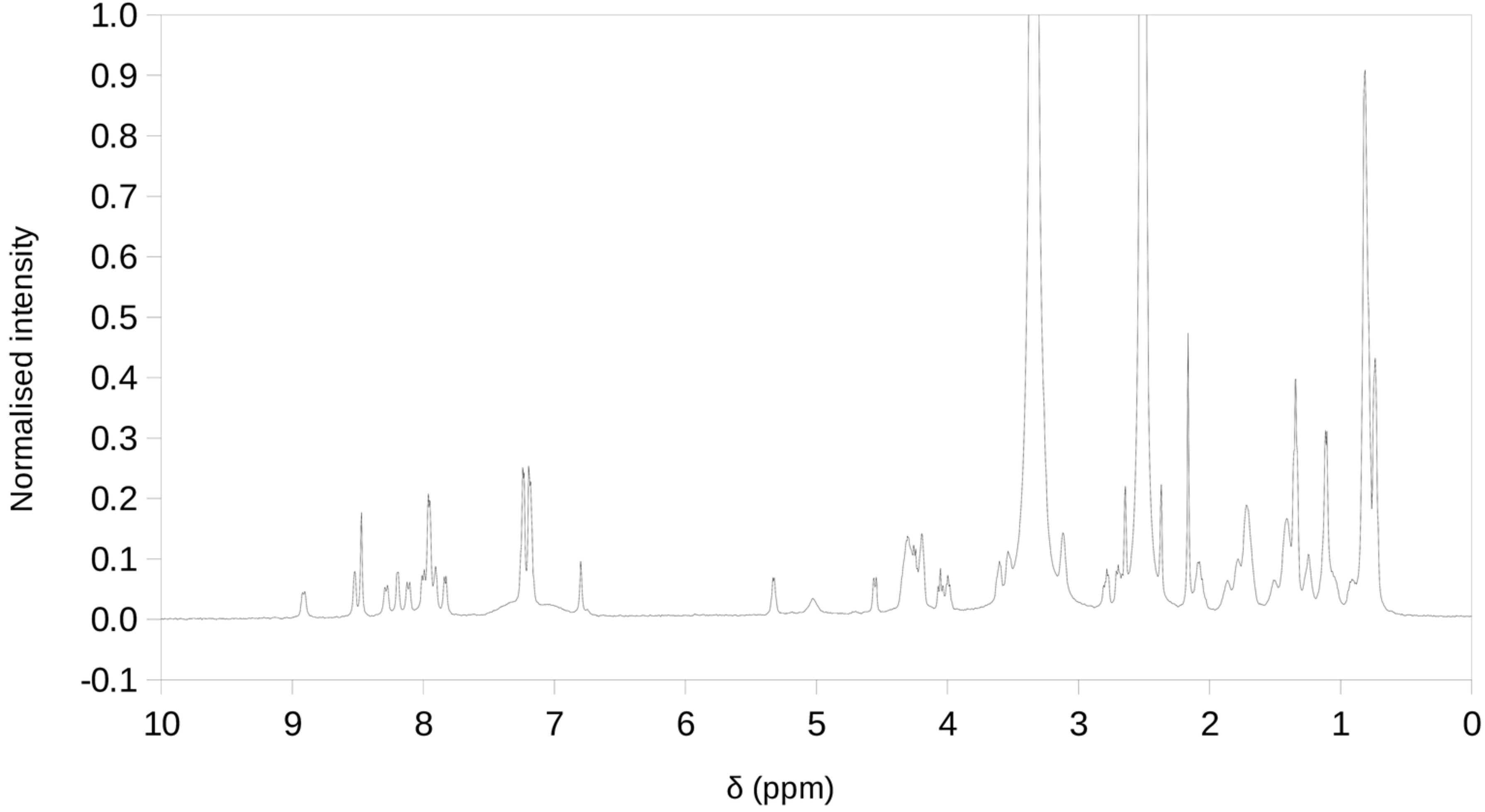

Fig. 72 ^{1}H NMR spectra obtained from compound **7**

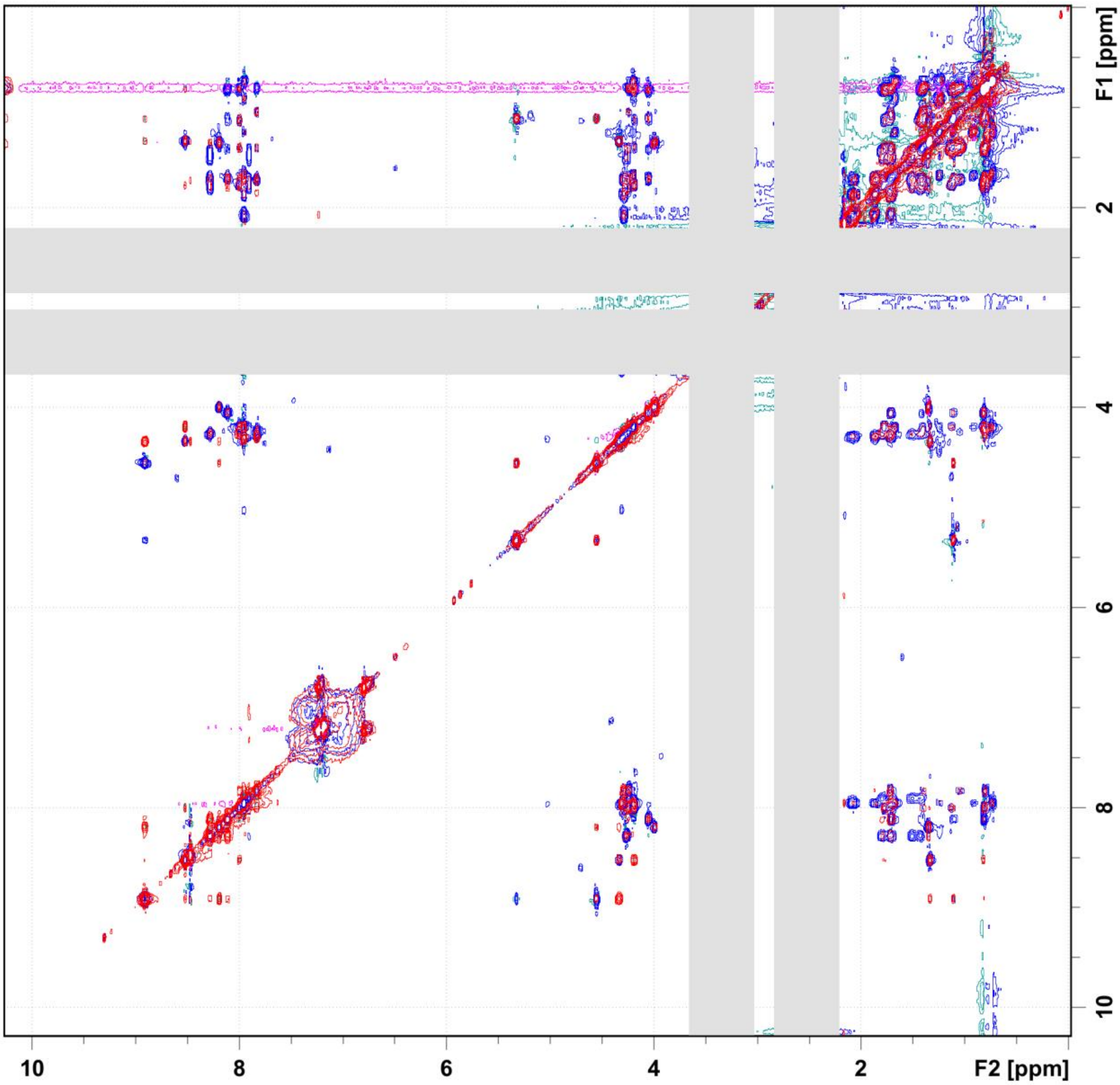

Fig. S73. ^{1}H-^{1}H TOCSY (blue) and ^{1}H-^{1}H NOESY (red) spectra obtained from compound **7**. For clarity, grey boxes obscure solvent signals.

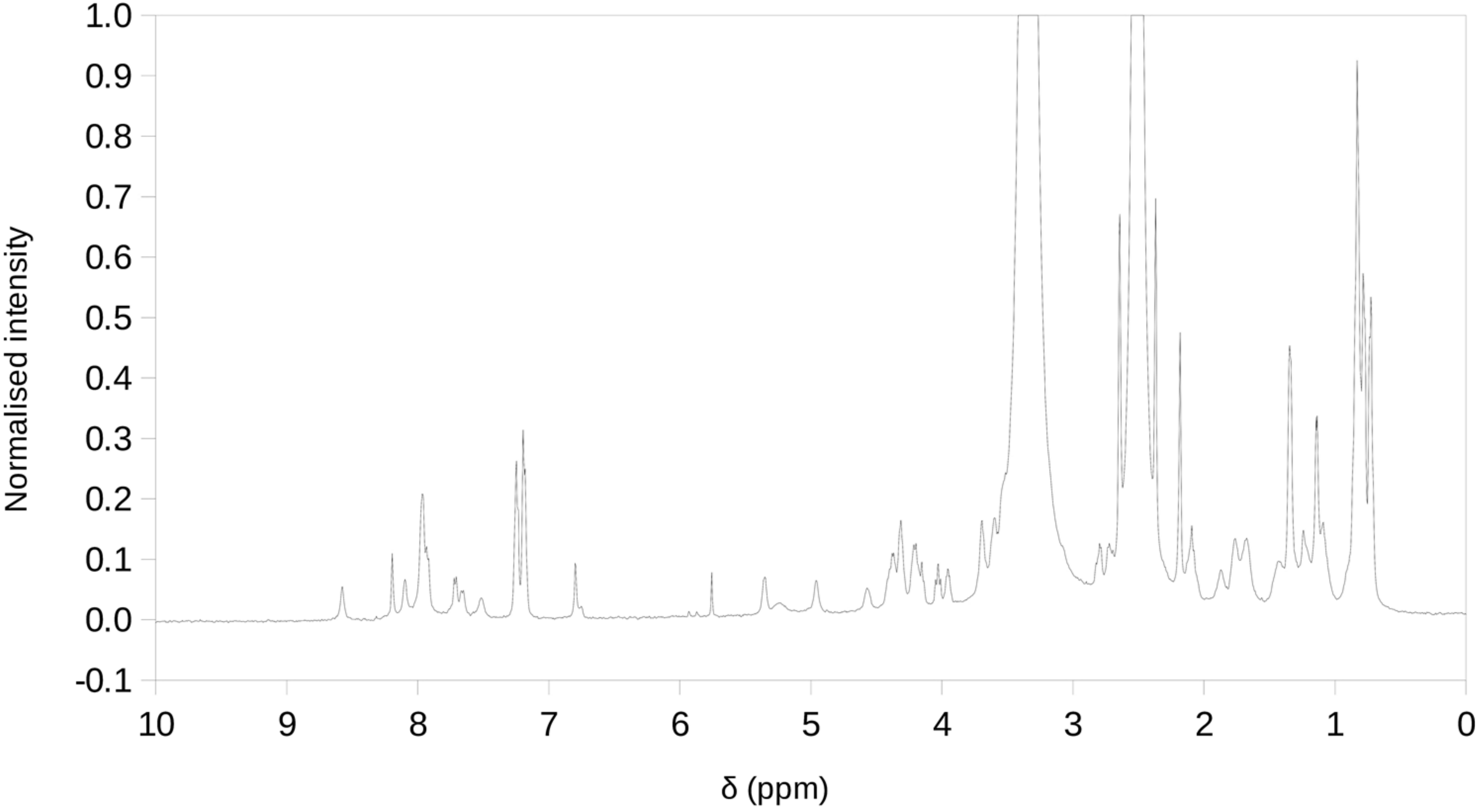

Fig. 74 ^{1}H NMR spectra obtained from compound **8**

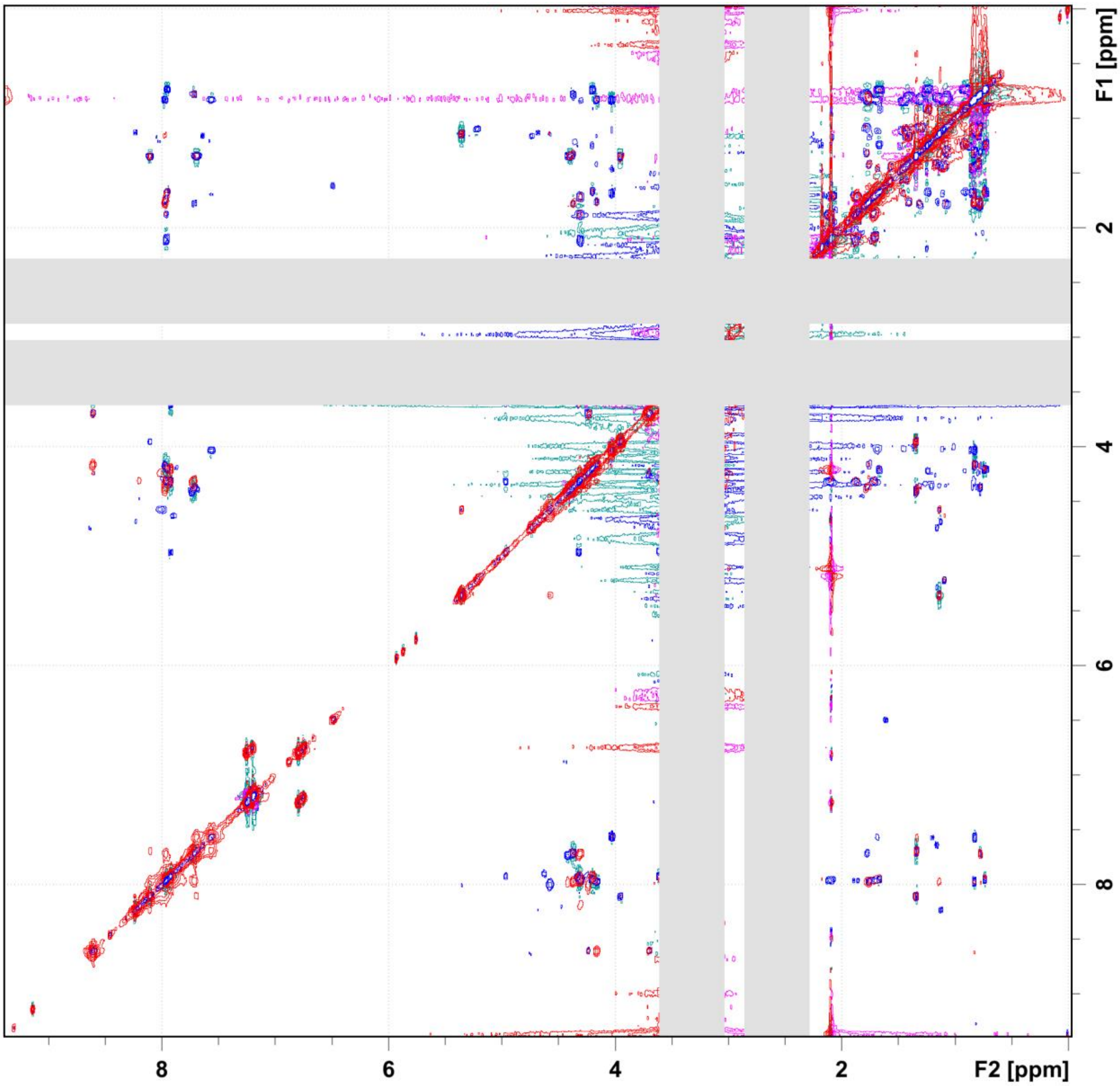

Fig. S75. ^{1}H-^{1}H TOCSY (blue) and ^{1}H-^{1}H NOESY (red) spectra obtained from compound **8**. For clarity, grey boxes obscure solvent signals.

^{1}H NMR spectra were from 1mM compounds 1-8 dissolved in DMSO-d_6. 128K complex points acquired at 300°C with 16 scans.

^{1}H-^{1}H TOCSY (blue) and ^{1}H-^{1}H NOESY (red) spectra were acquired from 1 mM compounds **1-8** dissolved in DMSO-d_6 at 300°C on a Bruker Avance III HD 500 MHz equipped with a room-temperature broadband probe. Spectra were 2048 and 192 complex points in the direct and indirect dimensions, respectively. For clarity, grey boxes obscure solvent signals.

VI. MIC testing (screening)

For MIC assays all peptides were dissolved in DMSO containing 0.002% polysorbate 80[7]. All bacteria were grown in Mueller Hinton broth (Oxoid) in triplicate. All incubations were at 37°C. Dilutions were carried out in triplicate. 100 µl of autoclaved Mueller Hinton broth was added to wells 2-12 in a 96-well plate. 200 µl of the peptide was added to well one at a concentration of 256 µg/mL. 100µl of peptide in well one was taken up and pipetted into well two. The mixture was then mixed via pipetting before 100µl was taken up and pipetted into well three. This process was repeated up to well 11. Once peptide was added to well 11 100 µl was taken up and then discarded ensuring the well 12 had no peptide present. Thus, the concentrations (in µg/mL) were: 256, 128, 64, 32, 16, 8, 4, 2, 1, 0.5, 0.25 and no peptide present. Each well was then inoculated with 100µl of bacteria that had been diluted to an OD600nm of 0.1. This was repeated three times. The 96-well plates were then incubated at 37°C for 24 hours. The MIC was determined to be the lowest concentration at which there was no growth visible.

For all the compounds in which the MIC lower than 1 µg/ml for the initial test, the above procedure was repeated at an altered initial concentration of 64 µg/ml. Therefore, the new concentrations for MIC were: 64, 32, 16, 8, 4, 2, 1, 0.5, 0.25, 0.125, 0.0625 and no peptide present. Vancomycin was used as a control.

To determine the MIC of *M. smegmatis* ATCC 607 an inoculum was shaken at 140rpm, 37°C in 5ml Middlebrook 7H9 broth (SIGMA) supplemented to 5% Middlebrook ADC (SIGMA) growth supplement for 3-4 days and harvested mid-late exponential phase (OD ~0.6). The harvested cells were washed once in fresh media and diluted 10-fold from the original volume. Then plated out in a 96-well plate as previously described, incubated at 37°C 140RPM with MIC readings taken after 72 hours.

In order to determine the effect of serum on antibacterial activity, the MIC of compounds **12** and **13** were measured in presence of 10% human serum using the above protocols. Both the compounds were pre-treated with 10% human serum (Sigma, H4522) for 30 mins and 2 hours. These pre-treated samples were used for MIC determination using Mueller Hinton Broth supplemented with 10% human serum.

VII. Antagonization assay

An antagonization assay was performed using Leu$_{10}$-teixobactin (**13**) and Lipid II as reported in literature.[7] MIC was tested using the protocols described in section VI.

Precursor	Molar ratio of precursor to Leu$_{10}$-teixobactin				
	0x	0.5x	1x	2x	5x
Lipid II	-	+	+	+	+

Table S3: Leu$_{10}$-teixobactin at 8x MIC exposed to increasing concentrations of lipid II. MIC was tested against the strain reported in ref 7. *S. aureus ATCC 29213.* Experiments were performed in triplicate.

VIII. MIC testing (extended panel)

Bacterial cultures were grown overnight in Mueller-Hinton Agar (MHA) plates and adjusted to a final concentration of 10^5 – 10^6 CFU/ml. 100 µl of inoculum in Meuller-Hinton broth (MHB) was mixed with equal volume of peptides (dissolved in MHB) at 2x their concentration in a 96 well plate. In parallel experiments, MIC values were determined in the media containing polysorbate 80 (0.002%, v/v) to

prevent non-specific adsorption of the peptides to plastic surfaces. The final peptides concentrations ranged from 0.0625 – 32 µg/ml. Positive and negative controls contained 200 µl of inoculum without any peptide dissolved in broth, respectively. The 96 well plates were then incubated at 37 °C for 24 h. All the experiments were performed in two independent duplicates and the MIC was determined as the lowest concentration in which no visible growth was observed. Minimum bactericidal concentration (MBC) was determined by plating out the dilution representing the MIC and concentrations up to 16x MIC on MHA plates kept at 37 °C for 24 h. The lowest concentration in which no visible colonies could be detected was taken as the MBC.

Compound ➔ / Strain 🡻		**(8)**	**(11)**	**(12)**	**(13)**	**(16)**	Vancomycin	Daptomycin
MRSA 1	MIC (with polysorbate 80)	4	1	0.25	0.25	1	1	0.5
	MIC (without polysorbate 80)	8	4	2	2	4	2	-
	MBC	16	4	1	2	2	-	-
MRSA 2	MIC (with polysorbate 80)	1	0.5	≤ 0.0625	≤ 0.0625	0.125	1	0.5
	MIC (without polysorbate 80)	4	2	2	2	2	2	-
	MBC	4	4	≤ 0.0625	≤ 0.0625	0.5	-	-
MRSA 3	MIC (with polysorbate 80)	1	0.25	≤ 0.0625	≤ 0.0625	0.5	1	0.5
	MIC (without polysorbate 80)	4	4	2	2	4	2	-
	MBC	2	2	0.125	≤ 0.0625	1	-	-
S. aureus	MIC (with polysorbate 80)	1	0.25	≤ 0.0625	≤ 0.0625	0.25	2	0.25
	MIC (without polysorbate 80)	4	2	1	1	2	4	-
	MBC	2	1	0.125	0.125	1	-	-
VRE 1	MIC (with polysorbate 80)	4	0.5	≤ 0.0625	0.25	2	>4	0.5
	MIC (without polysorbate 80)	8	4	1	2	4	>4	-
VRE 2	MIC (with polysorbate 80)	4	0.5	≤ 0.0625	0.25	2	>4	0.5
	MIC (without polysorbate 80)	8	4	1	2	4	>4	-

Table S4: MIC and MBC (in µg/mL) of the lead teixobactin analogues **8**, **11-13**, **16** and Daptomycin control against an extended panel of Gram positive bacteria in the presence and absence of polysorbate 80. Strain information: MRSA 1: MRSA ATCC 700699, MRSA 2: MRSA DR 42412 (sputum), MRSA 3: MRSA DM21455 (eye). MRSA 2 and MRSA 3 are clinical isolates. *Staphylococcus aureus ATCC 29213, Enterococcus faecalis,* (VRE 1: VRE ATCC 700802, VRE 2: VRE ATCC 29212).

IX. Time-dependent killing of bacteria by teixobactin analogues 12 and 13

Time-kill kinetics against MRSA DM21455 strains (clinical isolates from patients) was carried out in MHB. Cultures were grown overnight in MHA plates and adjusted to a final inoculum of $10^5 – 10^6$ CFU/ml in MHB (containing 0.002% v/v, polysorbate 80) with teixobactin analogues **12** and **13** maintained at a final concentration of 0.5 µg/ml. For vancomycin, the concentration was varied from 0.5 – 16 µg/ml without polysorbate 80. The tubes were then incubated at 37 °C. 100 µl of cell suspension was withdrawn at various time points (0, 2, 4, 8, 24 h), serially diluted (10^1-10^5 fold dilutions) and plated onto a MHA plates and incubated for 24 h at 37 °C. Colonies were then enumerated using a haemocytometer. Colony counting too numerous to count (>300 colonies) was taken as 10^{10} CFU. Average values from two independent experiments are reported.

X. Complex formation of teixobactin with lipid II and geranyl pyrophosphate

Complex formation of teixobactin analogues **8** (Ala$_{10}$-teixobactin) and **16** (Arg$_{10}$-teixobactin) with lipid II and geranyl pyrophosphate was performed using TLC as described previously.[7] Binding of teixobactin to lipid II and geranyl pyrophosphate was analysed by incubating 30 µL of 2 nmol of each precursor with 2 or 4 nmoles of teixobactin in 50 mM Tris/HCl, pH 7.5, for 30 min at room temperature. Complex formation was analysed by extracting unbound precursors from the reaction mixture with 30 µL n- butanol/6M pyridine acetate (pH 4.2) (2:1; vol/vol) followed by TLC analysis of the organic layer using chloroform/methanol/water/ammonia (88:48:10:1, v/v/v/v) as the solvent and detection of lipid/phosphate containing precursors by phosphomolybdic acid staining. The TLC figures represent the results obtained through three independent experiments.

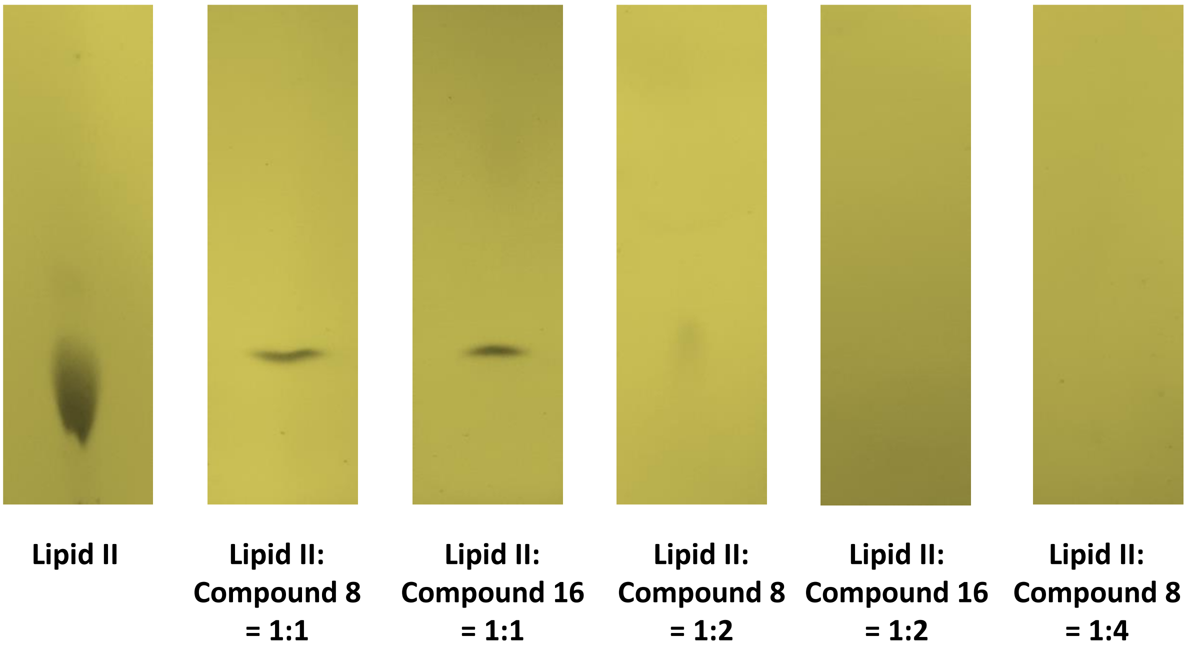

Figure S76: Binding of teixobactin analogues **8** (Ala$_{10}$-teixobactin) and **16** (Arg$_{10}$-teixobactin) with lipid II using the protocols described in literature.[7] Partial binding is observed when the ratio of lipid II to the analogue is 1:1 (indicated by lighter spots on the TLC) and complete binding is observed when the ratio of lipid II to the analogue is 1:2 in case of analogue **16** and 1:4 in case of analogue **8** (indicated by no spots on TLC).

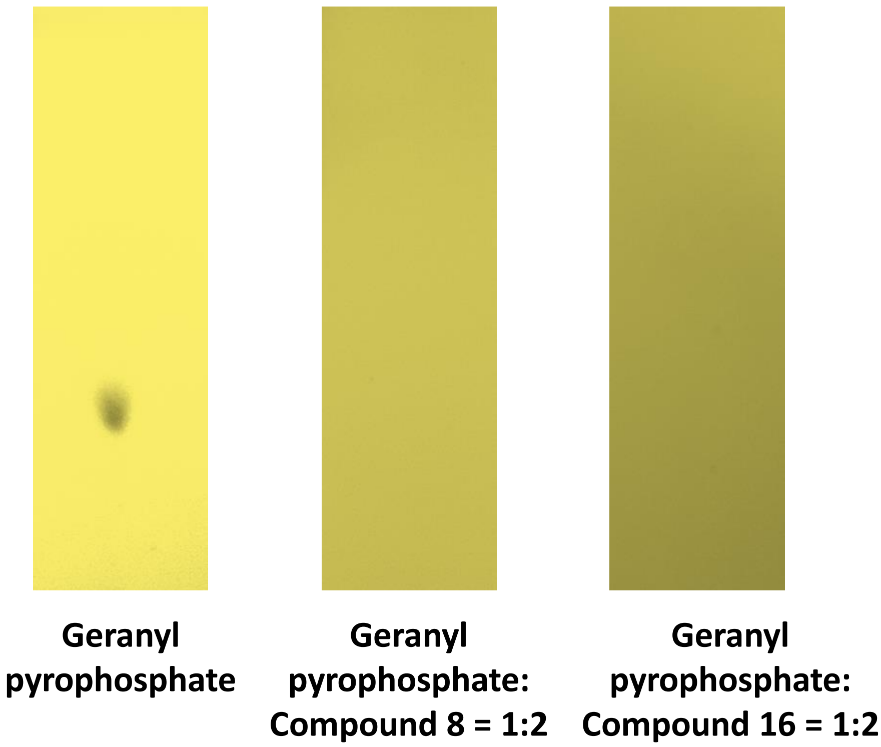

Figure S77: Binding of teixobactin analogues **8** (Ala$_{10}$-teixobactin) and **16** (Arg$_{10}$-teixobactin) with geranyl pyrophosphate using the protocols described in literature.[7] complete binding is observed when the ratio of the phosphate to the analogue is 1:2 (indicated by no spots on the TLC).

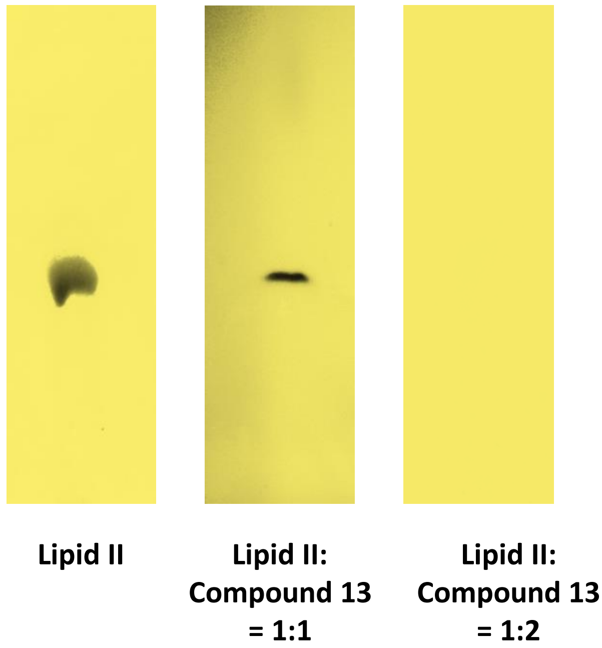

Figure S78: Binding of teixobactin analogue **13** (Leu$_{10}$-teixobactin) with lipid II using the protocols described in literature.[7] Partial binding is observed when the ratio of lipid II to the analogue is 1:1 (indicated by lighter spots on the TLC) and complete binding is observed when the ratio of lipid II to the analogue is 1:2 (indicated by no spots on TLC).

XI. Cytotoxicity assay

Cytotoxicity assay by Formazan bioreduction

HeLa cells were seeded in a 96-well plate at 10^4 cells/cm^2 density in Dulbecco's Modified Eagles Medium (DMEM) supplemented with 10% serum. The cells were repeatedly rinsed with Hank's Balanced Salt Solution (HBSS) prior to be exposed to different peptides in the range of 0.5 – 100 µM in HBSS 24 hrs post-seeding. Following 6 hrs of exposure to the teixobactin analogue, CellTiter 96 AQueous Nonradioactive Cell Proliferation Assay (Promega) was used according to the manufacturer's instructions.[8] Not ingested teixobactin analogue was removed by repeated washings with fresh medium. 20 µL of the combined MTS/PMS solution was added to 100 µL fresh medium in each well and plates were incubated for 3 hrs at 37°C. Absorbance was measured at 490 nm on Tecan Infinite M200 PRO plate reader with i-control 1.10 software (Molecular Devices).

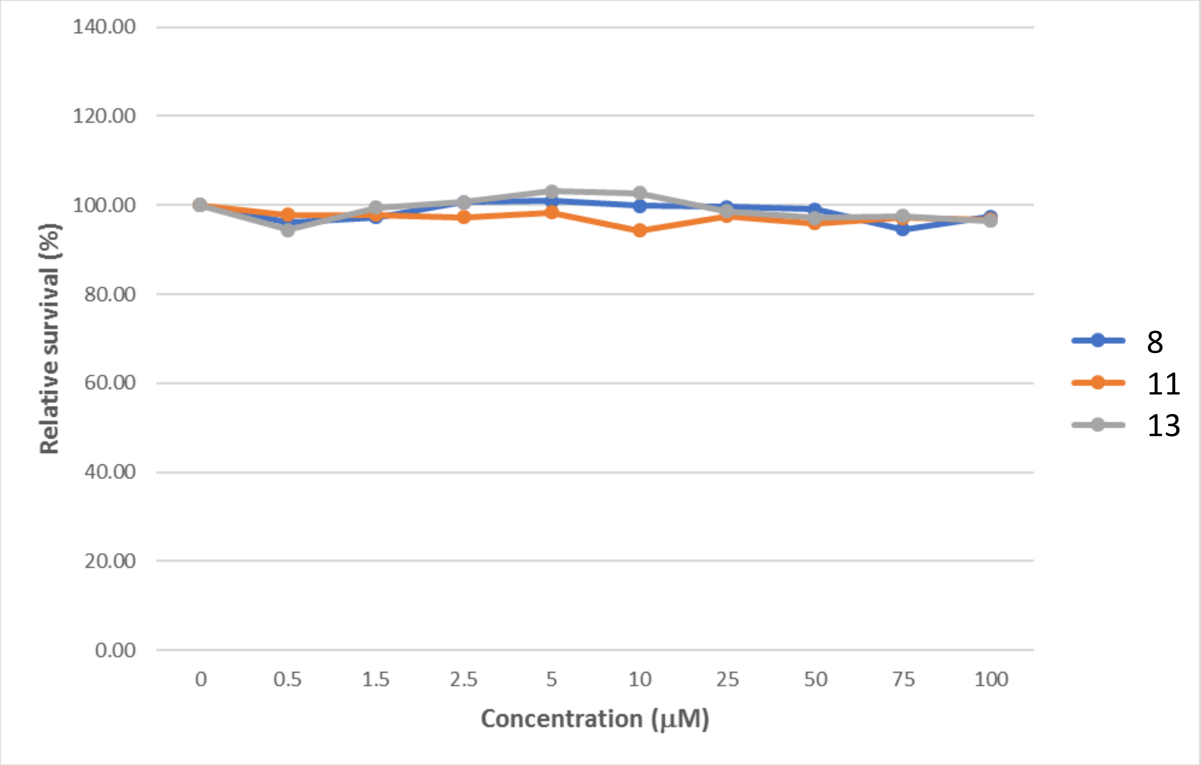

Figure S79: Toxicity results showing relative survival vs. Concentration (in µM) in HeLa cells for teixobactin analogues **8** (Ala_{10}-teixobactin), **11** (Val_{10}-teixobactin) and **13** (Leu_{10}-teixobactin).

XII. Haemolytic Assay Protocol

This assay was done at Singapore Eye Research Institute, Singapore. Hemolytic assay was performed on rabbit red blood cells (RBCs) immediately after collecting the blood samples from adult rabbits. All procedures for isolating blood from rabbits were approved by IACUC Singhealth and performed according to the standards of the Association for the Research in Vision and Ophthalmology.

Haemolytic activity of peptides was determined for rabbit red blood cells (rRBC), as reported before.[9] Rabbit erythrocytes were isolated from freshly collected blood samples and washed twice with sterile PBS. Two-fold serial dilutions of peptides (0.195 – 250 µg/ml) was mixed with rRBC (final concentration 4% v/v), incubated at 37°C for 1h and centrifuged at 3000 rpm for 5 minutes. The release

of hemoglobin in the supernatant was monitored by measuring the hemoglobin absorbance at 576nm. The readings from negative control (PBS and rRBC without any additives) and positive control (2% Triton-X100 and rRBC) were used as 0% and 100% haemolysis, respectively. Prolific pore forming and haemolytic melittin was used as comparator peptide. The data represents average value from triplicates experiments.

XIII. References:

1 A. Parmar, A. Iyer, C. S. Vincent, D. Van Lysebetten, S. H. Prior, A. Madder, E. J. Taylor and I. Singh, *Chem. Commun.*, 2016, **52**, 6060–6063.

2 W. F. Vranken, W. Boucher, T. J. Stevens, R. H. Fogh, A. Pajon, M. Llinas, E. L. Ulrich, J. L. Markley, J. Ionides and E. D. Laue, *Proteins Struct. Funct. Genet.*, 2005, **59**, 687–696.

3 T. Herrmann, P. Güntert and K. Wüthrich, *J. Mol. Biol.*, 2002, **319**, 209–27.

4 M. J. Abraham, T. Murtola, R. Schulz, S. Pall, J. C. Smith, B. Hess and E. Lindah, *SoftwareX*, 2015, **1**–**2**, 19–25.

5 S. Li and A. H. Elcock, *J. Phys. Chem. Lett.*, 2015, **13**, 2127–2133.

6 J. Xu and D. Van, *Biophys. J.*, 2017, **112**, 224–233.

7 L. L. Ling, T. Schneider, A. J. Peoples, A. L. Spoering, I. Engels, B. P. Conlon, A. Mueller, D. E. Hughes, S. Epstein, M. Jones, L. Lazarides, V. A. Steadman, D. R. Cohen, C. R. Felix, K. A. Fetterman, W. P. Millett, A. G. Nitti, A. M. Zullo, C. Chen and K. Lewis, *Nature*, 2015, **517**, 455–459.

8 A. H. Cory, T. C. Owen, J. A. Barltrop and J. G. Cory, *Cancer Commun.*, 1991, **3**, 207–12.

9 R. Lakshminarayanan, S. Liu, J. Li, M. Nandhakumar, T. T. Aung, E. Goh, J. Y. T. Chang, P. Saraswathi, C. Tang, S. R. B. Safie, L. Y. Lin, H. Riezman, Z. Lei, C. S. Verma and R. W. Beuerman, *PLoS One*, 2014, **9**, e87730.
